# Correction: Effect and cost-effectiveness of human-centred design-based approaches to increase adolescent uptake of modern contraceptives in Nigeria, Ethiopia and Tanzania: Population-based, quasi-experimental studies

**DOI:** 10.1371/journal.pgph.0003401

**Published:** 2024-06-13

**Authors:** The PLOS Global Public Health Staff

The S1 Text, S2, S3 and S4 Tables with comments are incorrectly uploaded. Please view the correct S1 Text, S2, S3 and S4 Tables below.

The publisher apologizes for the error.

## Supporting information

S1 Text (DOCX)

S2 Table (DOCX)

S3 Table (DOCX)

S4 Table (DOCX)
