# Supplementary material for: Effect and cost-effectiveness of human-centred design-based approaches to increase adolescent uptake of modern contraceptives in Nigeria, Ethiopia and Tanzania: Population-based, quasi-experimental studies
Source: PLOS Glob Public Health. 2023 Oct 18;3(10):e0002347. doi: 10.1371/journal.pgph.0002347 (PMC10584105; doi:10.1371/journal.pgph.0002347)
Supplement: S1 Text — includes: a series of result figures; a detailed description of the methodology of the Adolescents 360 outcome evaluation and of the cost-effectiveness study; methods and results for the analysis of trends in modern contraceptive use prevalence; and the analysis plan of the Adolescents 360 outcome evaluation. (DOCX) [file pgph.0002347.s001.docx]

**Effect and cost-effectiveness of human-centred design-based approaches to increase adolescent uptake of modern contraceptives in Nigeria, Ethiopia and Tanzania: population-based, quasi-experimental studies**

Catarina Krug, PhD, Melissa Neuman, ScD, James E Rosen, MA, Michelle Weinberger, MSc, Stefanie Wallach, MA, Mary Lagaay, MSc, Melanie Punton, MA, Annapoorna Prakash, MPH, Mussa Kelvin Nsanya, MS, Philip Ayieko, PhD, Saidi Kapiga, MD, Yewande P Ajayi, MPH, Emily E Crawford, MPH, Eskindir Tenaw, MSc, Mohammed Mussa, PhD, Som Kumar Shrestha, MSc, Christian Bottomley, PhD, James R Hargreaves, PhD, Aoife Margaret Doyle, PhD

Index

[List of tables 5](#_Toc139627271)

[List of figures 7](#_Toc139627272)

[Results tables and figures 9](#_Toc139627273)

[Methodology details of the Adolescents 360 Outcome evaluation 18](#_Toc139627274)

[Definition of exposure for the comparison of outcomes at endline between exposed and non-exposed 18](#_Toc139627275)

[Regression framework 19](#_Toc139627276)

[Northern Nigeria (Nasarawa) and Southern Nigeria (Ogun) 19](#_Toc139627277)

[Ethiopia and Tanzania 20](#_Toc139627278)

[Modifications made to the A360 outcome evaluation protocol 22](#_Toc139627279)

[Nigeria and Ethiopia 22](#_Toc139627280)

[Changes due to COVID-19 pandemic 22](#_Toc139627281)

[Other changes 22](#_Toc139627282)

[Tanzania 22](#_Toc139627283)

[Changes due to COVID-19 pandemic 22](#_Toc139627284)

[Other changes 23](#_Toc139627285)

[Methods and results for the analysis of trends in modern contraceptive use prevalence 24](#_Toc139627286)

[Nigeria 24](#_Toc139627287)

[Methodology 24](#_Toc139627288)

[Results 24](#_Toc139627289)

[Ethiopia 26](#_Toc139627290)

[Methodology 26](#_Toc139627291)

[Results 27](#_Toc139627292)

[Tanzania 27](#_Toc139627293)

[Methodology 27](#_Toc139627294)

[Results 27](#_Toc139627295)

[Methodology details of the Adolescents 360 costing and cost-effectiveness study 28](#_Toc139627296)

[Adolescents 360 Analysis Plan 29](#_Toc139627297)

[Investigators 29](#_Toc139627298)

[List of Abbreviations and Acronyms 29](#_Toc139627299)

[Introduction, key objectives and study design 29](#_Toc139627300)

[Research hypothesis 30](#_Toc139627301)

[Primary objectives 30](#_Toc139627302)

[Secondary objectives 30](#_Toc139627303)

[Study outline 31](#_Toc139627304)

[Study populations 31](#_Toc139627305)

[Inclusion/exclusion criteria 32](#_Toc139627306)

[Data collection tools 32](#_Toc139627307)

[Outline of statistical analysis 33](#_Toc139627308)

[Unit of statistical analysis 33](#_Toc139627309)

[Definitions and data manipulations 33](#_Toc139627310)

[Approach to Missing data 33](#_Toc139627311)

[Descriptive analysis 33](#_Toc139627312)

[Hierarchical or multilevel data 35](#_Toc139627313)

[Demographics and characteristics 35](#_Toc139627314)

[Analysis of the primary outcome 35](#_Toc139627315)

[Degree of self-reported length of time living in the community 36](#_Toc139627316)

[Analysis of secondary outcomes 36](#_Toc139627317)

[Stata commands 37](#_Toc139627318)

[Potential limitations 37](#_Toc139627319)

[General to all settings 37](#_Toc139627320)

[Analysis steps 37](#_Toc139627321)

[Methodology in Nigeria 38](#_Toc139627322)

[The A360 intervention 38](#_Toc139627323)

[Research hypothesis 38](#_Toc139627324)

[Study design 38](#_Toc139627325)

[A360 activities in Local government areas selected 40](#_Toc139627326)

[Baseline comparability between pairs of Local Government Area 43](#_Toc139627327)

[Regression framework 43](#_Toc139627328)

[Sampling weights 45](#_Toc139627329)

[Potential limitations – Nigeria specific 45](#_Toc139627330)

[Methodology in Ethiopia 45](#_Toc139627331)

[The A360 intervention 45](#_Toc139627332)

[Research hypothesis 46](#_Toc139627333)

[Study design 46](#_Toc139627334)

[A360 activities in kebeles selected 47](#_Toc139627335)

[Regression framework 48](#_Toc139627336)

[Sampling weights 49](#_Toc139627337)

[Potential limitations – Ethiopia specific 49](#_Toc139627338)

[Methodology in Tanzania 50](#_Toc139627339)

[The A360 intervention 50](#_Toc139627340)

[Research hypothesis 50](#_Toc139627341)

[Study design 50](#_Toc139627342)

[Regression framework 51](#_Toc139627343)

[Potential limitations – Tanzania specific 52](#_Toc139627344)

[Synthesis 52](#_Toc139627345)

[Appendix I: Outcomes of interest for the A360 Outcome Evaluation – Collected at baseline and endline 54](#_Toc139627346)

[Appendix II: Other outcomes of interest for the A360 Outcome Evaluation – Only collected at endline 56](#_Toc139627347)

[Appendix III: Summary of study designs in each country / site 58](#_Toc139627348)

[Appendix IV: Sample size calculations 59](#_Toc139627349)

[Nigeria 59](#_Toc139627350)

[Ethiopia 60](#_Toc139627351)

[Tanzania 61](#_Toc139627352)

[Appendix V: Modifications made to the A360 outcome evaluation protocol 62](#_Toc139627353)

[Changes due to COVID-19 pandemic 62](#_Toc139627354)

[Data collection – Population A 62](#_Toc139627355)

[Data collection – Population B 62](#_Toc139627356)

[Other changes 62](#_Toc139627357)

[Appendix VI: Socioeconomic index 64](#_Toc139627358)

[Data collection tools 64](#_Toc139627359)

[Appendix VII: STATA commands 65](#_Toc139627360)

[Stata commands – Nigeria specific 65](#_Toc139627361)

[Rural respondents 65](#_Toc139627362)

[Urban respondents 65](#_Toc139627363)

[Stata commands – Ethiopia specific 66](#_Toc139627364)

[Rural respondents 66](#_Toc139627365)

[Urban respondents 67](#_Toc139627366)

[Stata commands – Tanzania specific 67](#_Toc139627367)

[Rural respondents 68](#_Toc139627368)

[Urban respondents 68](#_Toc139627369)

[Appendix VIII: Dummy tables 70](#_Toc139627370)

[Appendix IX: Stata commands 84](#_Toc139627371)

[Declare survey design for dataset 84](#_Toc139627372)

[Descriptive tables and summaries 84](#_Toc139627373)

[Regression (fixed effects only) 84](#_Toc139627374)

[Regression accounting for clustering 84](#_Toc139627375)

[Robust standard errors 85](#_Toc139627376)

[Random effects model 85](#_Toc139627377)

[References 86](#_Toc139627378)

# List of tables

[Table S1 Defining exposure to A360 based on exposure questions in Nigeria, Ethiopia and Tanzania 18](#_Toc139627379)

[Table S2 Outcomes measured through CAPI (i.e. face-to-face), during the first section of the interview, and through CATI (i.e. phone), during the second section of the interview 22](#_Toc139627380)

[Table S3 Tanzania: Assumptions for key parameters used in sample size calculation, and final sample size calculation after accounting for design effect 23](#_Toc139627381)

[Table S4 Structure of the dataset used for investigating the effect of Adolescents 360 intervention on modern contraceptive use 35](#_Toc139627382)

[Table S5 Structure of the PSI monitoring data, used to define duration of A360 activities 36](#_Toc139627383)

[Table S6 Comparability of outcome evaluation study intervention and comparison LGAs in Ogun and Nasarawa states 39](#_Toc139627384)

[Table S7 Nigeria: Expected exposure to the A360 intervention, according to PSI team 40](#_Toc139627385)

[Table S8 Nigeria: Expected exposure to the A360 intervention, according to PSI monitoring data and PSI team info combined together 41](#_Toc139627386)

[Table S9 Nigeria: Adolescents 360 outcome evaluation baseline data 43](#_Toc139627387)

[Table S10 Definition of exposure to A360 interventions at endline in Nigeria 44](#_Toc139627388)

[Table S11 Degree of self-reported exposure to A360 (percentages) at endline, by comparison and intervention areas 44](#_Toc139627389)

[Table S12 Ethiopia: Characteristics of selected woredas 46](#_Toc139627390)

[Table S13 Ethiopia: Expected exposure to the A360 intervention, according to PSI team ^1^ 48](#_Toc139627391)

[Table S14 Ethiopia: Expected exposure to the A360 intervention, according to PSI monitoring data and PSI team info combined together 48](#_Toc139627392)

[Table S15 Definition of exposure to A360 interventions at endline in Ethiopia 49](#_Toc139627393)

[Table S16 List of administrative wards within Ilemela District, Mwanza 51](#_Toc139627394)

[Table S17 Definition of exposure to A360 interventions at endline in Tanzania 52](#_Toc139627395)

[Table S18 Number of events and girls reached per month and year in Ilemela district, Tanzania 52](#_Toc139627396)

[Table S19 Tanzania: Expected exposure to the A360 intervention 52](#_Toc139627397)

[Table S20 Adolescents 360 Theory of Change, outcomes of interest for the A360 Outcome Evaluation, and data collection tools 54](#_Toc139627398)

[Table S21 Adolescents 360 Theory of Change, outcomes of interest for the A360 Outcome Evaluation related to population A, and data collection tools 56](#_Toc139627399)

[Table S22 Adolescents 360 Theory of Change, outcomes of interest for the A360 Outcome Evaluation related to population B, and data collection tools 57](#_Toc139627400)

[Table S23 Summary of study design 58](#_Toc139627401)

[Table S24 Nigeria: revised mCPR estimates 59](#_Toc139627402)

[Table S25 Nigeria: estimated mCPR and sample size needed for sexually active 15-19 year olds 59](#_Toc139627403)

[Table S26 Nigeria: summary of endline survey design 59](#_Toc139627404)

[Table S27 Nigeria (Ogun): Details of sample size calculation 60](#_Toc139627405)

[Table S28 Nigeria (Nasarawa): Details of sample size calculation 60](#_Toc139627406)

[Table S29 Ethiopia: Table of assumptions for key parameters required for sample size calculations 60](#_Toc139627407)

[Table S30 Ethiopia: Final target sample size taking into account design effect 60](#_Toc139627408)

[Table S31 Ethiopia: sampling strategy 61](#_Toc139627409)

[Table S32 Tanzania: Table of assumptions 61](#_Toc139627410)

[Table S33 Tanzania: Final target sample size taking into account various estimates of design effect 61](#_Toc139627411)

[Table S34 Modifications made to the A360 surveys from baseline 63](#_Toc139627412)

[Table S35 Data collection tools to calculate the socioeconomic index in Nigeria, Ethiopia and Tanzania 64](#_Toc139627413)

[Table S36 Reasons for non-response and response rates at baseline and endline for girls aged 15-19 years (n, %) [51] 70](#_Toc139627414)

[Table S37 Reasons for non-response and response rates at baseline and endline for co-habiting adults (n, %) [51] 70](#_Toc139627415)

[Table S38 Percentage distribution of adolescent girl respondents by age, education, religion, socioeconomic status, access to phone and marital status (%, n) 71](#_Toc139627416)

[Table S39 Background characteristics of husbands and co-habiting adult respondents surveyed (%,n) 72](#_Toc139627417)

[Table S40 Sexuality, fertility and fertility preferences of adolescent girl respondents (Estimate, 95% Confidence Interval) 73](#_Toc139627418)

[Table S41 Family planning knowledge, attitudes and beliefs of co-habiting adult respondents’ surveyed 74](#_Toc139627419)

[Table S42 Family planning knowledge, attitudes and beliefs of adolescent girl respondents (%, 95% Confidence Interval) 75](#_Toc139627420)

[Table S43 Degree of self-reported exposure to A360 (percentages) at endline, by age, education, religion, socioeconomic status and access to phone (%,n) [52]. 77](#_Toc139627421)

[Table S44 Current use of modern contraception by age, education, religion, socioeconomic status and access to phone (%,n) 78](#_Toc139627422)

[Table S45 Percentage distribution of married and sexually active unmarried adolescent girls aged 15–19 years who currently use contraception, by method used (DHS definition) (%, 95% Confidence Interval) 79](#_Toc139627423)

[Table S46 Degree of self-reported exposure to A360 (percentages) at endline, by outcome variables [52]. 80](#_Toc139627424)

[Table S47 Percentage distribution of married and sexually active unmarried adolescent girls aged 15–19 years who ever used contraception, by method used (DHS definition) (%, 95% Confidence Interval) 81](#_Toc139627425)

[Table S48 Difference-in-difference estimates of A360 impact on mCPR, intervention and comparison sites (Nigeria only) [15]. 82](#_Toc139627426)

[Table S49 The estimates of A360 impact on mCPR (Ethiopia). 82](#_Toc139627427)

[Table S50 Degree of self-reported exposure to A360 by sexual and reproductive health outcomes [52]. 82](#_Toc139627428)

[Table S51 Declare survey design for datasets for each country. 84](#_Toc139627429)

# List of figures

[Figure S1 Pre- versus post-intervention comparison of primary and secondary outcomes and in Northern Nigeria: results of Poisson regression models 10](#_Toc139627430)

[Figure S2 Pre- versus post-intervention comparison of primary and secondary outcomes in Northern Nigeria: results of linear regression models 10](#_Toc139627431)

[Figure S5 Pre- versus post-intervention comparison of primary and secondary outcomes in Southern Nigeria: results of Poisson regression models 12](#_Toc139627432)

[Figure S4 Pre- versus post-intervention comparison of primary and secondary outcomes in Southern Nigeria: results of linear regression models 12](#_Toc139627433)

[Figure S5 Pre- versus post-intervention comparison of primary and secondary outcomes in Ethiopia: results of linear regression models 13](#_Toc139627434)

[Figure S6 Pre- versus post-intervention comparison of primary and secondary outcomes in Tanzania: results of linear regression models 13](#_Toc139627435)

[Figure S7 Exposed versus non-exponsed comparison of outcomes at endline in Northern Nigeria: results of Poisson regression models 14](#_Toc139627436)

[Figure S8 Exposed versus non-exponsed comparison of outcomes at endline in Northern Nigeria: results of linear regression models 14](#_Toc139627437)

[Figure S9 Exposed versus non-exponsed comparison of outcomes at endline in Southern Nigeria: results of Poisson regression models 15](#_Toc139627438)

[Figure S10 Exposed versus non-exponsed comparison of outcomes at endline in Southern Nigeria: results of linear regression models 15](#_Toc139627439)

[Figure S11 Exposed versus non-exponsed comparison of and outcomes at endline in Ethiopia: results of logistic regression models 16](#_Toc139627440)

[Figure S12 Exposed versus non-exponsed comparison of and outcomes at endline in Ethiopia: results of linear regression models 16](#_Toc139627441)

[Figure S13 Exposed versus non-exponsed comparison of outcomes at endline in Tanzania: results of logistic regression models 17](#_Toc139627442)

[Figure S14 Exposed versus non-exponsed comparison of outcomes at endline in Tanzania: results of linear regression models 17](#_Toc139627443)

[Figure S15 Secondary dataset, HMIS: Female clients aged 15–49 years using modern contraceptives at health facilities at the A360 outcome evaluation areas, between early 2016 and mid-2020 in Nasarawa State, Northern Nigeria (49 Wards) 25](#_Toc139627444)

[Figure S16 Secondary dataset, HMIS: Female clients aged 15–49 years using modern contraceptives at health facilities at the LGA, between early 2016 and mid-2020 in Ogun State (31 Wards) 26](#_Toc139627445)

[Figure S17 Secondary dataset, HMIS: Female clients aged 15–49 years using modern contraceptives at health facilities, between early 2016 and mid-2020 in Ogun and Nasarawa States (left) and in all States in Nigeria (right) 26](#_Toc139627446)

[Figure S18 Secondary dataset (PMA2020) – Modern contraceptive use among married and unmarried women aged 15-49 years in Ethiopia between 2015-2018 using A360 outcome evaluation definition 27](#_Toc139627447)

[Figure S19 Secondary dataset (Tanzania National Health Portal Data) – Modern contraceptive use among married and unmarried women aged 15-49 years in Tanzania between 2016-2020 using Tanzania National Health definition 28](#_Toc139627448)

[Figure S20 Hierarchical levels, by A360 outcome evaluation setting 31](#_Toc139627449)

[Figure S21 Participant flow diagram 34](#_Toc139627450)

[Figure S22 Number of girls reached per month and year in Ado-Odo/Ota LGA, Ogun State 40](#_Toc139627451)

[Figure S23 Number of girls reached per month and year in Doma LGA (left image) and Karu LGA (right image), Nasarawa State 40](#_Toc139627452)

[Figure S24 Map of Ogun State showing LGAs by their boundaries. Intervention LGAs in blue and comparison LGAs in red. 42](#_Toc139627453)

[Figure S25 Maps of Nasarawa State showing LGAs by their boundaries. Intervention LGAs in blue and comparison LGAs in red. 42](#_Toc139627454)

[Figure S26 A360 logo in Ethiopia 46](#_Toc139627455)

[Figure S27 Number of girls reached per month and year in Fentale woreda, Oromia Region 47](#_Toc139627456)

[Figure S28 Number of girls reached per month and year in Were Jarso woreda, Oromia Region 47](#_Toc139627457)

[Figure S29 Number of girls reached per month and year in Ada'a woreda, Oromia Region 47](#_Toc139627458)

[Figure S30 Number of girls reached per month and year in Lume woreda, Oromia Region 47](#_Toc139627459)

[Figure S31 A360 logo in Tanzania 50](#_Toc139627460)


# Results figures


| Figure S1 Pre- versus post-intervention comparison of primary and secondary outcomes and in Northern Nigeria: results of Poisson regression models | Figure S2 Pre- versus post-intervention comparison of primary and secondary outcomes in Northern Nigeria: results of linear regression models |
| --- | --- |
| 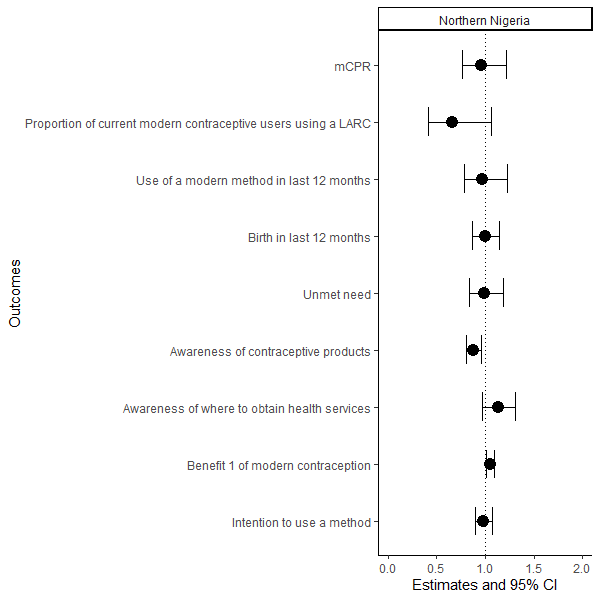 | 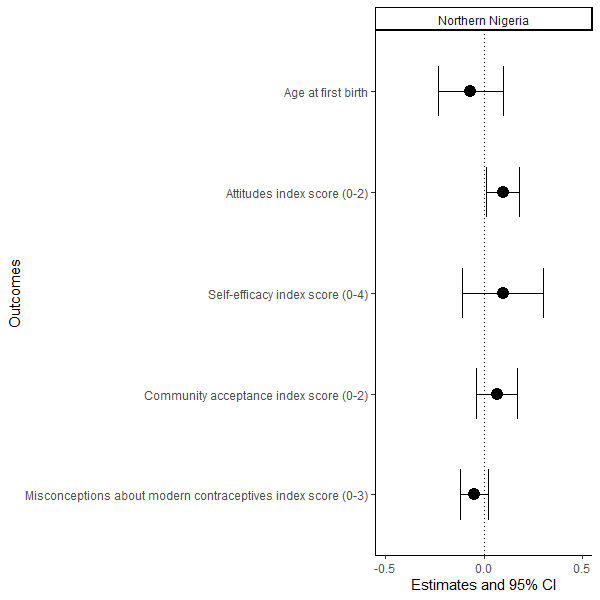 |

| Figure S5 Pre- versus post-intervention comparison of primary and secondary outcomes in Southern Nigeria: results of Poisson regression models | Figure S4 Pre- versus post-intervention comparison of primary and secondary outcomes in Southern Nigeria: results of linear regression models |
| --- | --- |
| 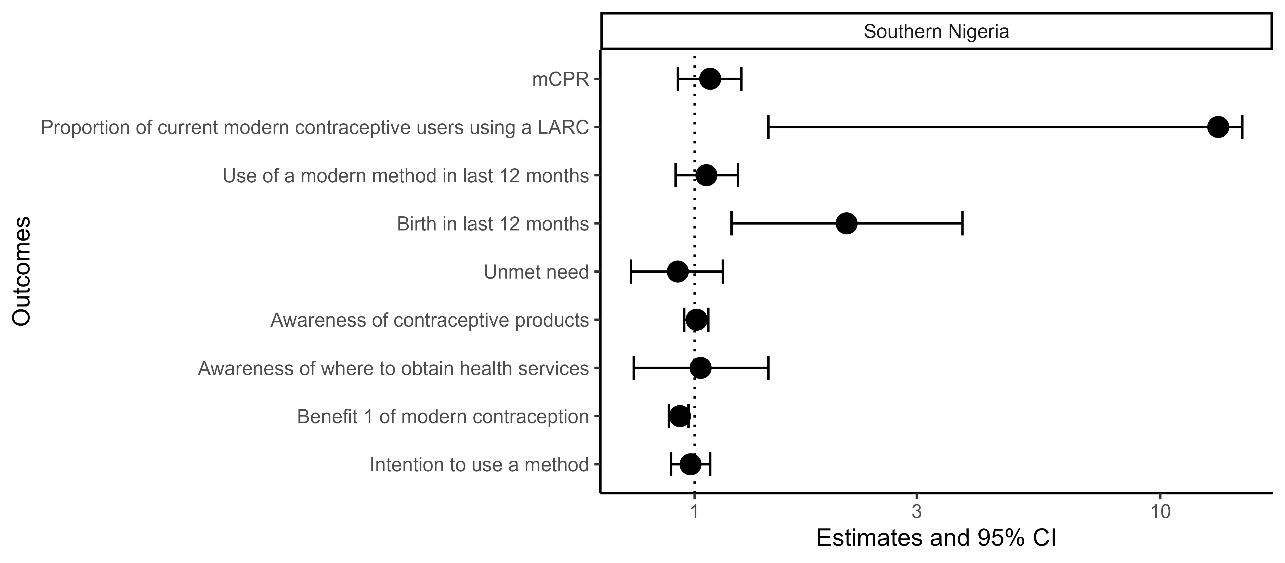  *X-axis is on the log scale | 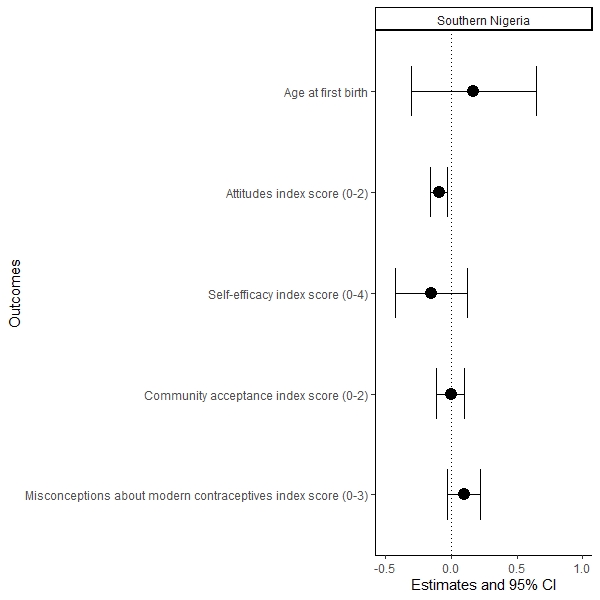 |

| Figure S5 Pre- versus post-intervention comparison of primary and secondary outcomes in Ethiopia: results of linear regression models | Figure S6 Pre- versus post-intervention comparison of primary and secondary outcomes in Tanzania: results of linear regression models |  |
| --- | --- | --- |
| 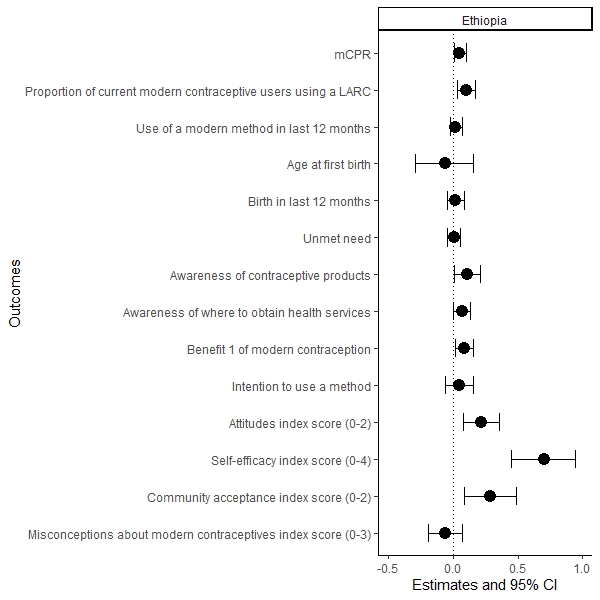 | 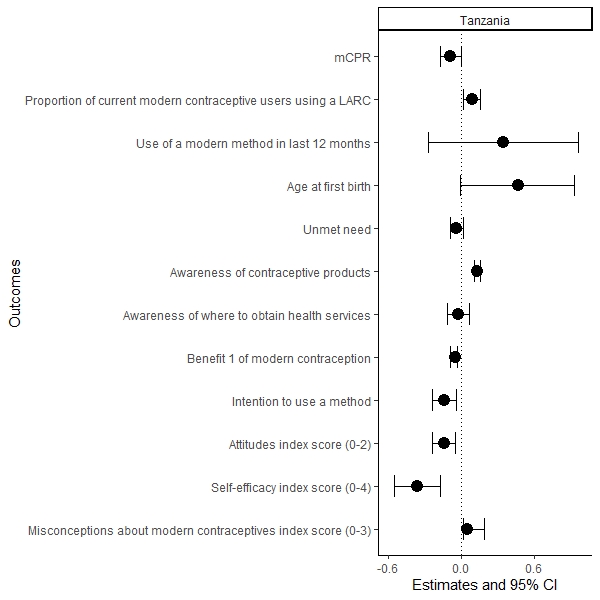 |  |


| Figure S7 Exposed versus non-exponsed comparison of outcomes at endline in Northern Nigeria: results of Poisson regression models | Figure S8 Exposed versus non-exponsed comparison of outcomes at endline in Northern Nigeria: results of linear regression models |
| --- | --- |
| 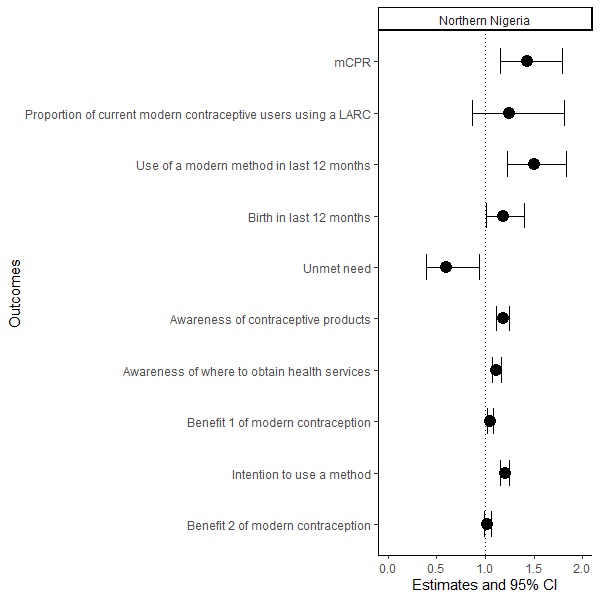 | 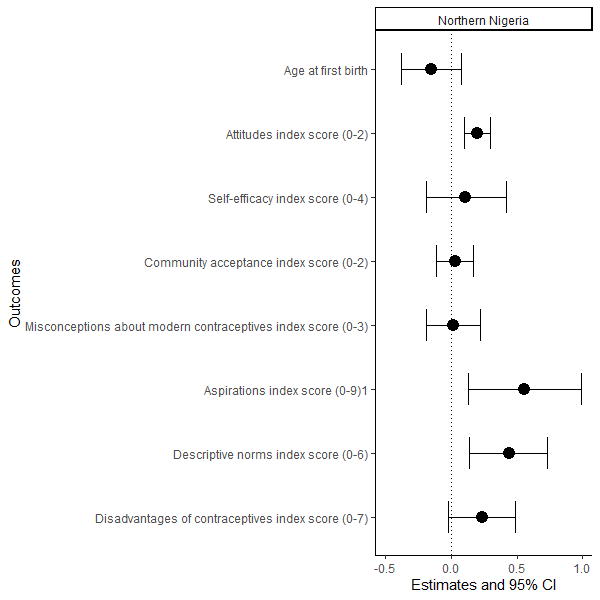 |

| Figure S9 Exposed versus non-exponsed comparison of outcomes at endline in Southern Nigeria: results of Poisson regression models | Figure S10 Exposed versus non-exponsed comparison of outcomes at endline in Southern Nigeria: results of linear regression models |
| --- | --- |
| 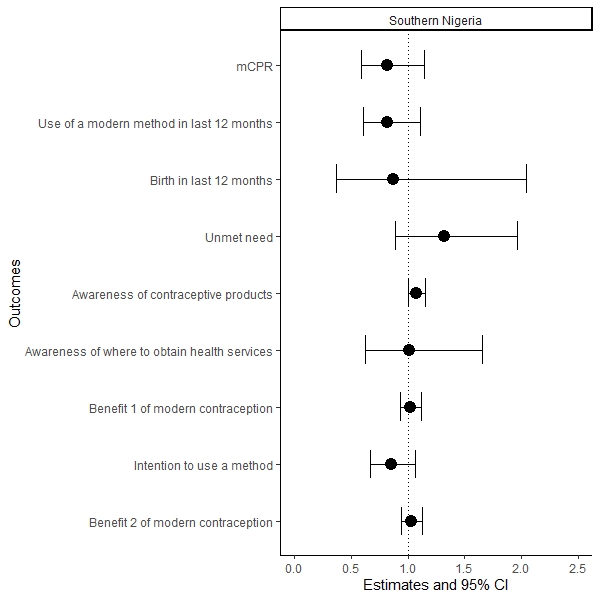 | 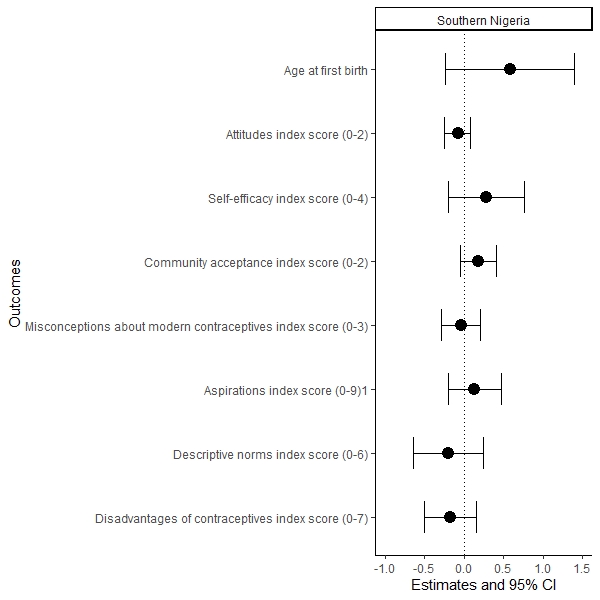 |

| Figure S11 Exposed versus non-exponsed comparison of and outcomes at endline in Ethiopia: results of logistic regression models | Figure S12 Exposed versus non-exponsed comparison of and outcomes at endline in Ethiopia: results of linear regression models |
| --- | --- |
| 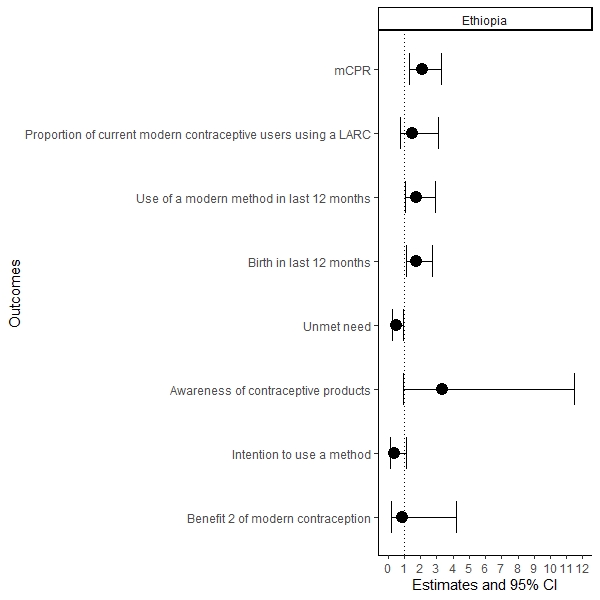 | 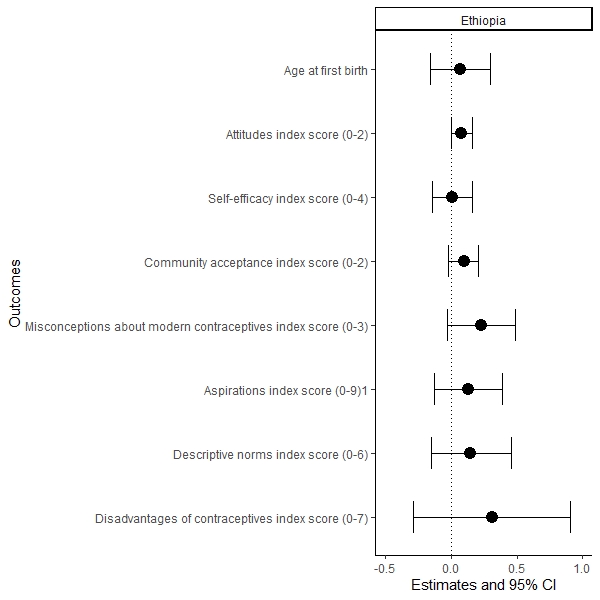 |

| Figure S13 Exposed versus non-exponsed comparison of outcomes at endline in Tanzania: results of logistic regression models | Figure S14 Exposed versus non-exponsed comparison of outcomes at endline in Tanzania: results of linear regression models |
| --- | --- |
| 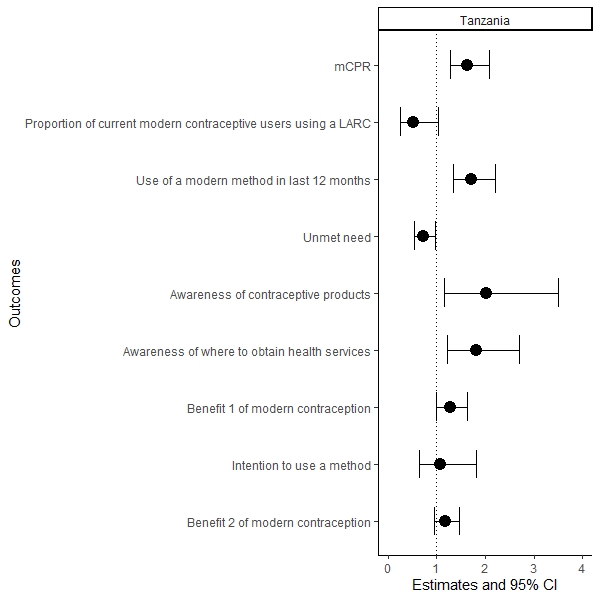 | 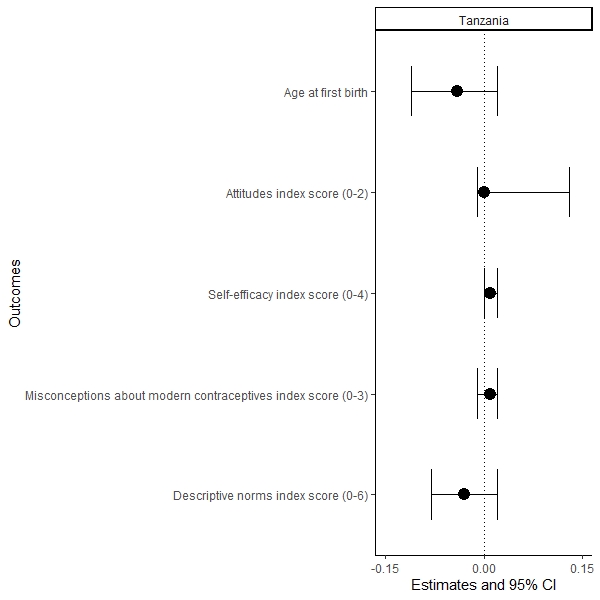 |

# Methodology details of the Adolescents 360 Outcome evaluation


## Definition of exposure for the comparison of outcomes at endline between exposed and non-exposed

We used a series of questions to rank individuals by their level of engagement with the A360 interventions that are available in the place where they live. Exposure questions used in endline surveys in Nigeria were defined by the LSHTM OE team members with Itad Process evaluation members as well as with Population Services International (**PSI**) members in January (Ethiopia and Tanzania) and February 2020 (Ethiopia). The final definition of exposed and non-exposed girls according to endline exposure questions are presented in **Table S1**.

Table S1 Defining exposure to A360 based on exposure questions in Nigeria, Ethiopia and Tanzania

| **Question** | **Exposure Questions** | **Exposed girl** | **Girl not exposed** |
| --- | --- | --- | --- |
| **1 Nasarawa** |  |  |  |
| 1.1 | Have you heard about a program called Matasa Matan Arewa (MMA)? | Answers “Yes” to Q1.1 and “Yes” to Q1.3 or Q1.4 | Answers “Yes” to Q1.1 but not to Q1.4 or Q1.3  Answers “No”, “Don’t know” or does not respond to Q1.1 |
| 1.2 | Do you recognize this logo?  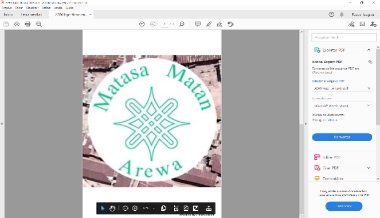 | N/A^1^ | As above |
| 1.3 | Have you participated in Life, Family, Health (LFH) sessions? | Answers “Yes” to Q1.3 and “Yes” to Q1.1 | As above |
| 1.4 | Did you have a one-on-one counselling with a trained provider? | Answers “Yes” to Q1.4 and “Yes” to Q1.1 | As above |
| **2 Ogun** |  |  |  |
| 2.1 | Have you heard about a program called 9JA girls? | Answers “Yes” to Q2.1 and “Yes” to Q2.2 or Q2.4 | Answers “Yes” to Q2.1 but not to Q2.4 or Q2.2  Answers “No”, “Don’t know” or does not respond to Q2.1 |
| 2.2 | Do you recognize this logo?  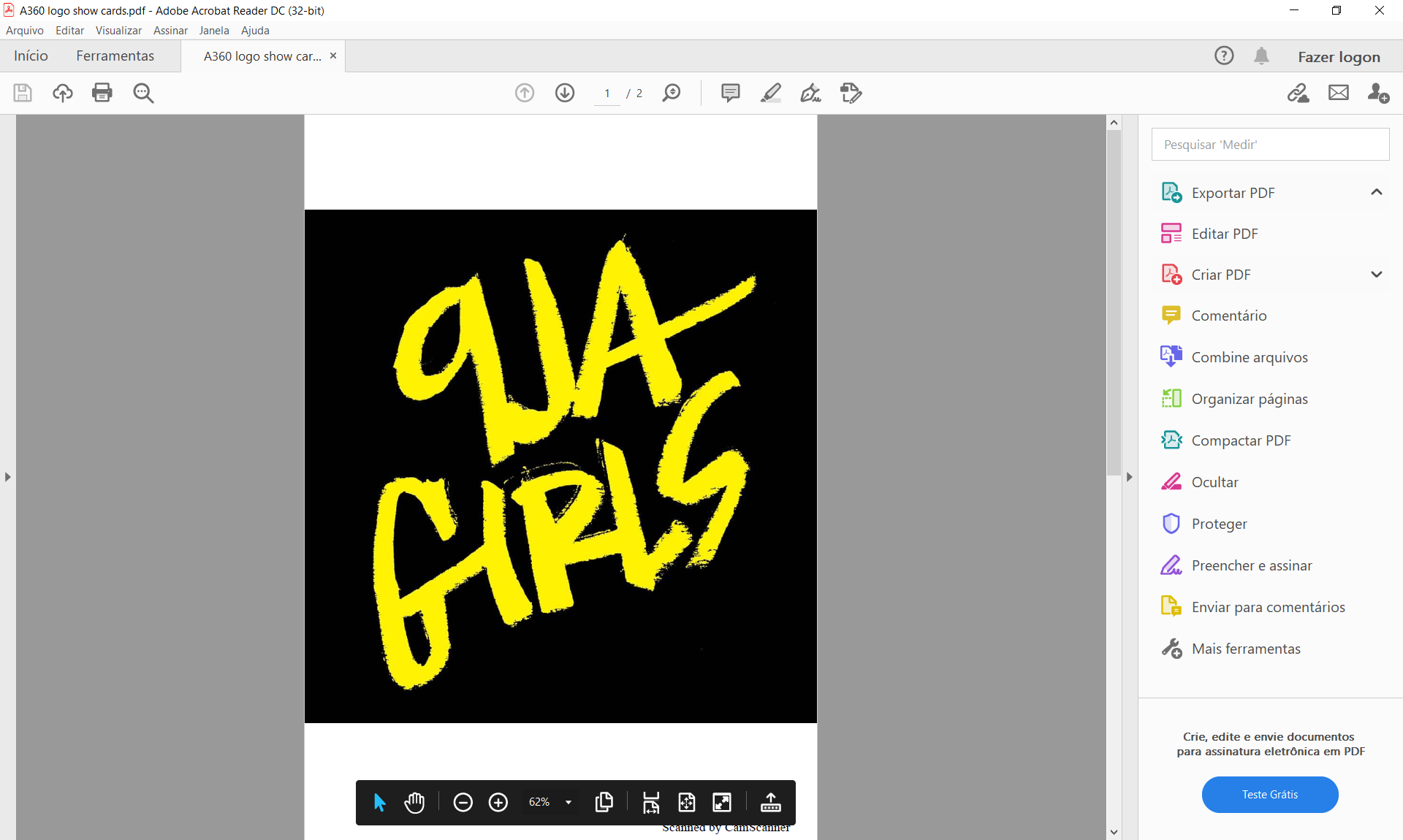 | Answers “Yes” to Q2.2 and “Yes” to Q2.1 | As above |
| 2.3 | Have you participated in Life, Love & Health (LLH) classes? | N/A^2^ | As above |
| 2.4 | Did you receive one-on-one counselling from a trained provider? | Answers “Yes” to Q2.4 and “Yes” to Q2.1 | As above |
| **3 Oromia** |  |  |  |
| 3.1 | Have you ever heard of ‘Smart Start’? | Answers “Yes” to Q3.1 and “Yes” to Q3.2/ Q3.3/ Q3.4/ Q3.5 | Answers “Yes” to Q1 but not to Q3.2/ Q3.3/ Q3.4/ Q3.5 or  Answers “No”, “Don’t know” or does not respond to Q3.1 |
| 3.2 | Have you ever seen any of these images?   | Answers “Yes” to Q3.2 and “Yes” to Q3.1 | As above |
| 3.3 | Have you ever seen any of these images?   | Answers “Yes” to Q3.3 and “Yes” to Q3.1 | As above |
| 3.4a | Have you heard about or seen a ‘goal card’? | Answers “Yes” to Q3.4a, disagrees with Q3.4b and answers “Yes” to Q3.1 | As above |
| 3.4b | Please tell me if you ‘agree’ or ‘disagree’ with the following statement "I heard about the ‘goal card’ but I do not know what it is" |  | As above |
| 3.5 | Do you have a ‘goal card’? | Answers “Yes” to Q3.5 and “Yes” to Q3.1 | As above |
| **4 Mwanza** |  |  |  |
| 4.1 | Heard about health project with pineapple as a symbol (no/yes) | Answers positively to Q4.1 and to Q4.3 or Q4.4 (Recognises at least one sentence) | Answers positively to Q4.1 but not to Q4.3 or Q4.4 (Does not recognise sentences) |
| 4.2 | Heard about Kuwa Mjanja (no/yes) | Answers positively to Q4.2 and to Q4.3 or Q4.4 | Answers positively to Q4.2 but not to Q4.3 or Q4.4 |
| 4.3 | Attended a meeting, event or workshop where Kuwa Mjanja was mentioned or pineapple displayed (no/yes)  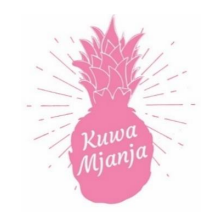 | Answers positively to Q4.3 and to Q4.1 or Q4.2 | Answers positively to Q4.3 but not Q4.1 or Q4.2 |
| 4.4 | Heard sentences from the nanasi story (1 to 4 sentences) | Answers positively to Q4.4 and to Q4.1 or Q4.2 | Answers positively to Q4.4 but not Q4.1 or Q4.2 |

^1^Due to the similarity between MMA symbol and Arewa symbol, only Q1.1 and Q1.3 as well as Q1.1 and Q1.4 were used to determine exposure in Nasarawa State.

^2^Due to the similarity in reported exposure to this question among those who answered “Yes” to Q2.1 in comparison (17.5%) and intervention sites (19.6%), only Q2.1 and Q2.2 as well as Q2.1 and Q2.4 were used to determine exposure in Ogun State.

## Regression framework

### Northern Nigeria (Nasarawa) and Southern Nigeria (Ogun)

#### Analysis of main outcome

To evaluate the impact of A360 on these outcomes, two types of analysis were conducted. The main analysis measured the impact of the A360 program from baseline to endline on each outcome. In this analysis regression models were fitted with three explanatory variables – time (0 baseline and 1 endline), A360 (0 comparison and 1 intervention area) and an interaction term between time and A360. The secondary analysis, measured the impact of self-reported exposure to the A360 program at endline, having therefore one explanatory variable – exposure (0 not exposed and 1 exposed), and contained only data from intervention areas at endline. These are described below.

#### The impact of the A360 program from baseline to endline

Two datasets were used per State (Ogun and Nasarawa), one with baseline data from the intervention and comparison sites, and the other with endline data from the two sites. Datasets were appended, and a dummy variable (i.e. time) identified whether the survey was conducted at baseline or at endline. Another dummy variable identified whether respondents were interviewed in an A360 site intervention or comparison site (i.e. A360).

Following [11], we used modified Poisson regression models with robust standard errors (at the EA level):

log(λ_i_) = β_0_ + β_1_Time_i_ + β_2_A360_i_ + β_3_Time_i_ × A360_i_

- λ_i_ = risk of outcome for individual i
- exp(β_0_) is the risk at baseline (time ‘0’) in a comparison area (A360 ‘0’);
- exp(β_1_) is the risk ratio comparing contraceptive use between endline (time ‘1’) and baseline (time ‘0’) in a comparison area;
- exp(β_2_) is the risk ratio comparing contraceptive use in the intervention area (A360 ‘1’) or in the comparison area (A360 ‘0’) at baseline (time ‘0’);
- and exp(β_3_) is the effect of A360 beyond the time effect [12].

Comparison and intervention LGA were selected in pairs, therefore the (descriptive and regression) analysis were conducted separately for each matched pair. The pairs were Ado-Odo/Ota (Ix) and Shagamu LGAs (Cx) in Ogun state, Doma (Ix) and Toto (Cx), as well as Karu (Ix) and Nasarawa LGAs (Cx) in Nasarawa state. For Nasarawa state, the main result was the effect of time (endline, time ‘1’, compared to baseline, time ‘0’) on mCPR over all intervention (A360 ‘1’) and comparison LGAs (A360 ‘0’), i.e. the four LGAs were analysed together in one model. The reason for this was that the study was powered to detect an impact overall for all four LGAs. A variable indicating pair (Toto and Doma ‘1’ and Nasarawa and Karu ‘2’) was added to the model to identify the paired comparison and intervention sites.

We adjusted for the following demographic variables, which are associated with contraceptive use according into the literature: age, education level, living children, religion and wealth quintile [13]. Age ranges from 15–19 years; Wealth Quintile^[[1]](#footnote-6)^ ranges from poorest (1^st^ and 2^nd^ quintiles) to richest (4^th^ and 5^th^ quintiles); education was categorised into ‘1’ secondary or higher education and ‘0’ qur'anic only, primary, and no education; living children was categorised into ‘1’ respondents with at least one child, and ‘0’ for no living children; religion was categorised into ‘1’ Catholic or Protestant/Other Christian and ‘0’ Muslim, Traditional, No religion, or others.

#### The impact of the self-reported exposure to the A360 program at endline

We first described modern contraceptive use among girls who reported being exposed to A360 and those who reported not being exposed at endline. We then used Poisson regression models with robust standard errors (at EA level) to assess the strength of association between self-reported exposure (exposure ‘0’, some exposure ‘1’) and the use of modern contraception (outcome):

log(λ_i_) = β_0_ + β_1_Exposure_i_

We adjusted for the following demographic variables: age, education level, living children, religion and wealth quintile [13]. The analysis was restricted to endline data, and to intervention areas only.

We used similar models to the one above to assess the effect of exposure on use within last 12 months and on proportion of LARC users.

#### Analysis of secondary outcomes

Binary secondary outcomes were evaluated as described for the main outcome:

1. The impact of the A360 program from baseline to endline.
2. The impact of the A360 program at endline among girls that reported exposure to the program.

For continuous outcomes we used linear regression models:

Y_i_ = β_0_ + β_1_Predictor

where Y_i_ is the predicted outcome for the i^th^ girl; β_0_ is the predicted value when β_1_=0 and β_1_ is the change in outcome associated with a one-unit increase in the predictor. As with the Poisson regression models, we used robust standard errors to adjust for clustering at the level of the EA.

#### Sampling weights

The probability of selection was the same for all households because the same number of households was selected from each EA, and each EA has approximately 100 households. Therefore, since all eligible girls were selected per household there was no need to use sampling weights.

### Ethiopia and Tanzania

#### The association between time (baseline versus endline) and primary and secondary outcomes

Two datasets were used, one with baseline data, and the other with endline data. Datasets were appended, and a dummy variable (e.g. time) identified whether the survey was conducted at baseline (time ‘0’) or at endline (time ‘1’).

We calculated mCPR at the PSU level, which was Kebele in Ethiopia and Street in Tanzania and then used linear regression models with mCPR (at the PSU level) as the outcome, and Time and PSU as predictors, as follows:

Y_it_ ~ Normal($\mu_{it}, \sigma^{2}$)

$\mu_{it}$ = $\alpha$ + βTime + $\sum_{i} \gamma_{i} Indicator(PSU = i)$

where $\mu_{it}$ is the predicted mCPR for the i^th^ PSU at time t (baseline or endline); $\alpha$ is the intercept; β represents the effect of Time (endline, Time ‘1’, vs baseline, Time ‘0’); and $\gamma_{i}$is the effect of PSU, which is included to account for variation in mCPR between PSUs and therefore increase precision.

The rational for doing a PSU-level analysis was that i) it is simple/robust – multilevel models sometimes do not converge ii) it generate risk difference estimates which are easier to interpret than odds ratios, particularly when the outcome is common as in this analysis. We adjusted for the following demographic variables, which are associated with contraceptive use according to the literature: age, education level, parity, religion and wealth quintile [13]. These were added to the model at the PSU level (e.g. average age per PSU).

#### The association between the A360 program and self-reported exposure to the program, at endline

We first described modern contraceptive use among girls who reported being exposed to A360 and those who reported not being exposed at endline. We then used logistic regression models to assess the strength of association between self-reported exposure (exposure ‘0’, some exposure ‘1’) and the use of modern contraception (outcome):

Y_i_ ~ Bernoulli(p_i_)

logit(p_i_) = $\alpha$ + β Exposure_i_ +$\sum_{j} \gamma_{j} Indicator(PSU = j)$

where p_i_ is the probability of modern contraceptive use for the i^th^ girl, $\alpha$is the intercept; β is the regression coefficient for Exposure and corresponds to the natural logarithm of the odds ratio between exposed and not exposed girls; and $\gamma_{j}$ is the PSU effect included to account for clustering of observations. We adjusted for the same demographic variables that were included in the primary analysis. We also used logistic regression models to assess the effect of exposure on other binary outcomes such as use within last 12 months and proportion of LARC users.

To assess the effect of exposure on continuous outcomes such as age at first birth and aspirations index score, we first calculated the mean (95%CI) among girls who reported being exposed to A360 and among those who reported not being exposed at endline. We then used linear regression models with exposure as the predictor, as follows:

Y_i_ ~ Normal($\mu_{i}, \sigma^{2}$)

$\mu_{i}$ = $\alpha$ + βExposure+ $\sum_{j} \gamma_{j} Indicator(PSU = j)$

where $\mu_{i}$ is the predicted outcome for the i^th^ girl ; $\alpha$ is the intercept; β reflects the effect of Exposure (exposed ‘1’, vs not exposed ‘0’); $\gamma_{j}$ is the PSU fixed effect included to account for clustering within PSUs. We adjusted for the same demographic variables mentioned for the model above.

#### Sampling weights

Ethiopia data was analyzed accounting for sampling weights as follows:

$$w_{j}=\frac{M}{m_{j}\times n}$$

where,

w_j_ = Sampling weight for girls in household j

M = Total number of households in the sampling frame

m_j_ = Total number of households in Kebele *j*

n = Number of Kebeles sampled

Sampling weights were calculated by the data collection team at baseline and endline.

#### Analysis of secondary outcomes

All secondary outcomes measured at both baseline and endline were evaluated as described for the main outcome:

- The association between time (baseline versus endline) and primary and secondary outcomes;
- The association between self-reported exposure to the program and primary and secondary outcomes, at endline.

For those outcomes which were only measured at endline, we only analysed them as described in option (B) above, which only uses endline data.

# Modifications made to the A360 outcome evaluation protocol

## Nigeria and Ethiopia

### Changes due to COVID-19 pandemic

The endline surveys for the A360 outcome evaluation were to be administered through Computer Assisted Personal Interviewing (CAPI; i.e. face-to-face interview) in 2020, as was done during baseline surveys in 2017. Due to the COVID-19 pandemic, we had to ensure that field implementation at endline was carried out with appropriate safeguards in place. Therefore, at endline, CAPI was used for the first part of the survey, and Computer Assisted Telephone Interviewing (CATI; i.e. phone survey) was used for the second part of the survey. Each section took a maximum of 20 minutes duration. Participants had the option to consent only to the first section (i.e. CAPI); in Nigeria, 20% took this offer, and 3% in Ethiopia. The second part of the survey occurred immediately after the first. The phone survey was conducted immediately after the face-to-face interview, to ensure the identity of participants (interviewer will ideally see the girl in the distance). Answers to sensitive questions were provided in a non-disclosive categorical format (i.e. 1,2,3; or a,b,c). The endline questionnaire was reviewed in detail at a meeting held on 4 March 2020, where all the evaluation teams, as well as PSI and donors had opportunity to input into revisions. **Table S2** shows which outcomes were collected in CAPI or in CATI. Note that primary outcomes related to mCPR use were all asked in CAPI, so there are no instrumentation differences between baseline and endline.

### Other changes

At endline, the questionnaire included questions on the exposure to the intervention and on aspirations as linking contraception use to girls’ life goals was a major feature of the intervention.

Table S2 Outcomes measured through CAPI (i.e. face-to-face), during the first section of the interview, and through CATI (i.e. phone), during the second section of the interview

| **Outcomes collected in full CAPI survey *(original pre-COVID questionnaire)*** | **Outcomes collected in CAPI followed by CATI survey (reduced questionnaire due to COVID-19 restrictions)** | **Section** | **Notes** |
| --- | --- | --- | --- |
| mCPR | Yes | 1^st^ CAPI |  |
| % of LARC users among current users | Yes | 1^st^ CAPI | New outcome at endline but can be calculated using baseline data |
| Use in last 12 months | Yes | 1^st^ CAPI | New outcome at endline but can be calculated using baseline data |
| Unmet need | Yes | 1^st^ CAPI |  |
| Age specific fertility rates | Yes (partial; see note) | 1^st^ CAPI | Reduced number of questions compared to full CAPI |
| Age at first birth | Yes | 1^st^ CAPI |  |
| Aspirations | Yes (partial; see note) | 2^nd^ CATI | New outcome at endline – no baseline data; Reduced number of questions compared to full CAPI |
| Community acceptance | Yes (partial; see note) | 2^nd^ CATI | Reduced number of questions compared to full CAPI |
| Agency/ Self-efficacy | Yes (partial; see note) | 2^nd^ CATI |  |
| Attitudes | Yes (partial; see note) | 2^nd^ CATI |  |
| Benefits | Yes | 1^st^ CAPI |  |
| Access to contraceptive services and products | Yes | 2^nd^ CATI |  |

## Tanzania

### Changes due to COVID-19 pandemic

Following the confirmation of the first case of COVID-19 in Tanzania on the 16th of March 2020, all public gatherings were banned. This led to the complete halt of A360 activities for the next three months in the region. In the meantime, a revised model of intervention was proposed and piloted by PSI, to meet the girls’ need safely, this revised model included in-clinic events only since they attracted only small crowds, in which case enforcement of social distancing is possible. Instead of mobilization through schools and public announcements, the Kuwa Mjanja Queens along with the Community Health Workers resolved to conduct door-to-door visits. The in-clinic events were modified to ensure that girls spent no more than 30 minutes in facilities to minimize risk, including a short ‘inspirational talk’ which contains a shorter version of the ‘know your body’ and ‘know your path’ messaging to encourage girls to think about their life goals.

The mode of data collection in baseline and endline surveys differed somewhat because of modifications to reduce the risk of COVID-19 transmission. At endline, the second section of the questionnaire was administered by telephone, and personal protective equipment (PPE) such as face masks were used during the first, in-person, section of the questionnaire.

### Other changes

The mCPR estimates used in our original sample size calculations were obtained from PSI and were based on projections of mCPR using the most recent DHS estimates. They are presented in the analysis plan on pages 68 and 69 of this supplementary material. Effect estimates are based on an analysis conducted by MW. The assumptions used are outlined in **Table S3**.

In Ilemela district, among sexually active 15–19-year-olds, we have assumed that because of A360, mCPR will increase from 27% to 33% between 2017 and 2019. This represents an absolute increase of 6% and a relative increase of 22% between 2017 and 2019 in A360-exposed girls. A sample size of 1,217 sexually active girls aged 15–19 years would give us 90% power to detect this difference based on the assumptions in **Table S3**.

Taking into account the sampling design, estimated non-response and the fact that not all adolescent girls will be currently sexually active, the final target sample size is 4,980 girls aged 15–19 years**.** In this scenario, we have estimated that the design effect will be 1·5.

We set out to equally divide across 30 ‘streets’ in 15 wards the sample target of 4,980 female participants aged 15–19 years. Based on assumptions derived from local census data we estimated that 34% of households have a female aged 15–19 years so we would need to enumerate 485 households per street to find approximately 166 adolescent girls per street. Our sampling strategy assumed we would identify 10 households per GPS point and an average of three eligible adolescent girls (one of whom is sexually active) per household cluster.

Following sampling in the first two wards, we found these assumptions largely overestimated the number of eligible girls identified per street using our planned strategy. In order to achieve the desired sample size, we revised our sampling strategy as described below.

Based on interim analysis of data from four wards, mCPR was estimated to be higher (49%) than our original estimate (27%). We revised our sample size calculations accordingly (**Table S3**). In Ilemela district, among sexually active 15–19-year-olds, we have assumed that because of A360, mCPR will increase from 49% to 60% between 2017 and 2019. This represents an absolute increase of 11% and a relative increase of 22% between 2017 and 2019 in A360-exposed girls. A sample size of 519 sexually active girls aged 15–19 years would give us 90% power to detect this difference based on the assumptions. Taking into account the sampling design, estimated non-response and the fact that not all adolescent girls will be currently sexually active, the final target sample size is 3,269 girls aged 15–19 years. In this scenario, we have estimated that the design effect will be 1·5. Following revision of sample size calculations based on interim baseline survey results, we estimated that sampling two streets from each of the remaining 13 wards (26 streets) and visiting all households to identify eligible girls would be sufficient to reach our revised target sample size.

However, for the endline survey, due to logistic reasons one of the wards in baseline had to be dropped at endline leading to the survey area spanning over 14 wards and 30 streets.

Table S3 Tanzania: Assumptions for key parameters used in sample size calculation, and final sample size calculation after accounting for design effect

| **Scenario** | **Original**  **90% power to detect 22% increase in mCPR (26·7% to 32·7%)** | **Revised (final)**  **90% power to detect 22% increase in mCPR (48·9% to 59·7%)** |
| --- | --- | --- |
| Proportion of 15–19-year-old females who are married (or living together with partner) | 21·7% (PSI data) | 5·9%^1^ |
| Proportion of 15-19-year-old females who are unmarried (not currently married) | 78·3 % (PSI data) | 94·1%^1^ |
| Proportion of married 15–19-year- old females who report sexual activity in past year | 97% (TDHS 2015-16 – all ages married) | 91·5%^1^ |
| Proportion of unmarried 15–19-year-old females who report sexual activity in past year | 24·8% (PSI data) | 22·2%^1^ |
| Proportion of 15–19-year- old females who report sexual activity in past year | 40·4% (PSI data) | 26·2%^1^ |
| Proportion of sexually active girls who are married | 51·8% (PSI data) | 20·5%^1^ |
| Proportion of sexually active girls who are unmarried | 48·2% (PSI data) | 79·5% |
| Target sample of sexually active 15–19-year-old girls | 1,217 | 519 |
| Total sample size of 15–19-year-old girls  Effective sample size  Includes estimated non-response (10%)  Includes non-sexually active girls | 3,314 | 2,179 |
| Design effect | 1·5 | 1·5 |
| Sample size (effective sample size * design effect) | 4,971 | 3,269 |

^1^ Estimated from interim baseline survey

# Methods and results for the analysis of trends in modern contraceptive use prevalence

## Nigeria

### Methodology

We used a difference in differences analysis in our impact evaluation, and the validity of the impact estimate depends on the mCPR time trend being the same in both intervention and comparison LGAs [15]. To assess whether this assumption was true, we compared trends over time in study LGAs, using secondary datasets. This comparison was made visually, using graphs.^[[2]](#footnote-7)^

The only dataset available to estimate trends in contraceptive use at the LGA level was Health Management Information System (HMIS) data from female clients aged 15–49 years using modern contraceptives at health facilities between early 2016 and mid-2020. We obtained three datasets: the first referring to Nasarawa State (146 Wards included), the second referring to Ogun State (238 Wards included), and the third referring to the whole country (37 States). Data was aggregated by Ward and year for state-level data and aggregated by state and year for national-level data.^[[3]](#footnote-8)^

HMIS aims to provide data for continued monitoring of the health system’s performance, existing since the 1960s in Nigeria [16]. HMIS collects data on over 100 indicators. In family planning, there are three indicators and around 15 data elements [17, 18]. Data is collected at the primary level of health service delivery (i.e. primary health care units), and then it is forwarded to the district and zonal levels.

HMIS has the advantage of providing continuous estimates for various health indicators across all levels of a country. However, it has several flaws in terms of completeness, timeliness and accuracy [19-22], which may explain the differences of its estimates of contraceptive use compared to DHS estimates over time [17, 23]. These differences may also be caused by differences in the indicator definition. Finally, HMIS data may be more representative of the population of married women of reproductive age than of the population of unmarried women, as married women are more likely to obtain their methods of modern contraception at the health facility compared to unmarried women. This means that the HMIS data is more likely to represent the true trend data for the population of interest in Nasarawa State, where married women were targeted, than for Ogun State, where unmarried women were targeted. Despite this limitation, we still believe these data can be used to help understand trends over time and contextualize the outcome evaluation findings.

### Results

The only data available to estimate trends in contraceptive use at the LGA level was HMIS data from female clients aged 15–49 years using modern contraceptives at health facilities, which is presented in **Figure S15** for Nasarawa State and in **Figure S16** for Ogun State, for each of the LGAs selected for the outcome evaluation. **Figure S17** shows national trends as well as trends in Nasarawa and Ogun States more generally.

There were secular increases in mCPR across Nigeria and the study regions, and within the study LGAs. Pair 1 LGAs had similar levels and trends in numbers of contraceptive users aged 15–49 between 2016 and 2017. A gap in number of contraceptive users then emerged between pair 1 LGAs, which seemed to widen around the start of A360 activities in Doma (I) in June 2019. On the other hand, in pair 2, Nasarawa (C) had a much lower level of contraceptive use when compared to Karu (I). Trends in terms of numbers of contraceptive users aged 15–49 years between 2016 and 2017 appeared to go in opposite directions, with numbers increasing in Nasarawa (C) and decreasing in Karu (I). A360 activities started in Karu (I) in April 2018, but number of contraceptive users in Nasarawa (C) appeared to increase more than Karu (I; **Figure S1**5), following national trends (**Figure S17**).

In Ogun State, LGAs had different trends in numbers of contraceptive users aged 15–49 between 2016 and 2017 with a sharp rise in users in Ado-Odo/Ota (I) and no change in users in Shagamu (C). The trend of increasing users continued in Ado-Odo/Ota (I) up until 2019 with only a small increase in users in Shagamu (C). A360 activities started in Ado-Odo/Ota in December 2017, however, the increase in modern contraceptive users appeared to start earlier than that (**Figure S16**), following national trends (**Figure S17**).

Figure S15 Secondary dataset, HMIS: Female clients aged 15–49 years using modern contraceptives at health facilities at the A360 outcome evaluation areas, between early 2016 and mid-2020 in Nasarawa State, Northern Nigeria (49 Wards)


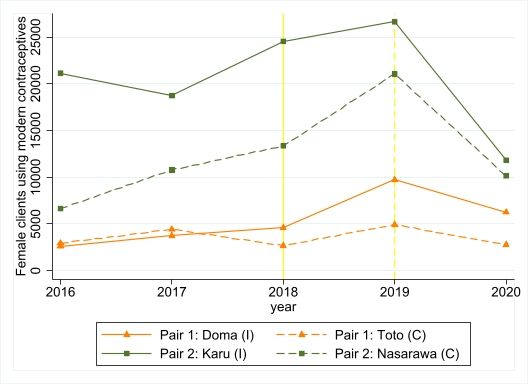


A360 activities started in 2019 in Doma, pair 1 (dashed yellow line), and in 2018 in Karu, pair 2 (solid yellow line)

Figure S16 Secondary dataset, HMIS: Female clients aged 15–49 years using modern contraceptives at health facilities at the LGA, between early 2016 and mid-2020 in Ogun State (31 Wards)


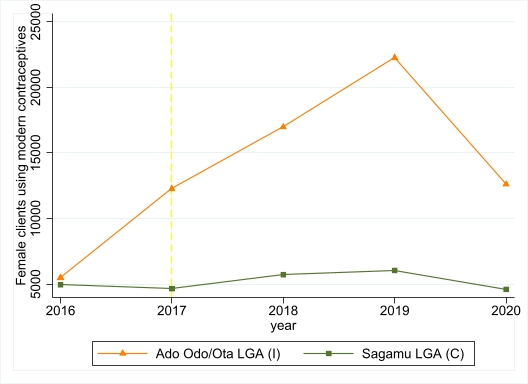


A360 activities started in 2017 in Ado-Odo/Ota (dashed yellow line).

Figure S17 Secondary dataset, HMIS: Female clients aged 15–49 years using modern contraceptives at health facilities, between early 2016 and mid-2020 in Ogun and Nasarawa States (left) and in all States in Nigeria (right)


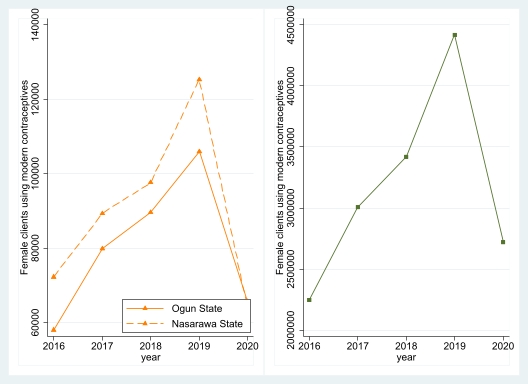


## Ethiopia

### Methodology

In a secondary analysis, data on modern contraceptive use available from other sources for the time period 2015-2018 were examined to assess whether changes in mCPR in A360 communities (2017-2020) reflect the overall trend in mCPR or whether mCPR appears to have increased more than would be expected during this time period. This was done because study outcomes were measured in intervention areas only and there was no comparison group, so observed changes in mCPR could be due to secular trends or other influences [24].

Data analyses were conducted in July 2020. After evaluating all datasets available, we decided to estimate trends in modern contraceptive use using Performance Monitoring for Action 2020 (PMA2020) dataset. PMA2020 was launched in 2013 to monitor the progress of the FP2020 initiative [25] and is implemented at the national level in Ethiopia. PMA2020 conducted multiple surveys asking questions on fertility, contraceptive use and other related measures. We analysed PMA2020 datasets from 2015 (round 3) to 2018 (round 6). We used the same mCPR definition used in the outcome evaluation. We focused on women aged 15-49 years, for greater precision in estimates (i.e. smaller 95%CI), as PMA2020 surveys have a small sample of married girls aged 15-19 years.

### Results

The secondary data analysis of trends in Ethiopia did not show a clear trend in modern contraceptive use between 2015 and 2018 at national level (PMA2020 population-based surveys). Fig**ure S18** describes modern contraceptive use among women aged 15-49 years in Ethiopia between 2015-2018. There seems to be an upward trend in mCPR, but the 95% confidence interval for mCPR estimates overlap, showing that it did not differ between years. Therefore, the increase in mCPR that we observed between baseline and endline surveys within the implementation woredas was larger than overall trends in mCPR, but this dataset is not directly comparable to our target population.

Figure S18 Secondary dataset (PMA2020) – Modern contraceptive use among married and unmarried women aged 15-49 years in Ethiopia between 2015-2018 using A360 outcome evaluation definition


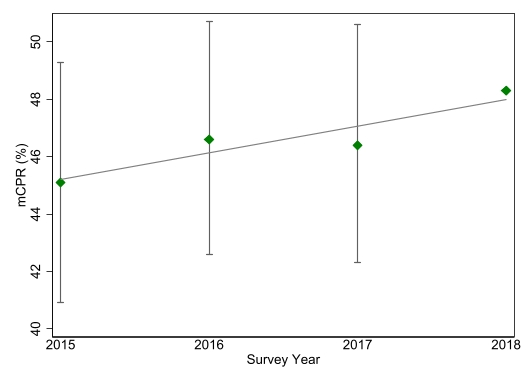


Note: Values are weighted for sampling weights

## Tanzania

### Methodology

Publicly available data on monthly mCPR among married and unmarried women aged 15–49 years group from 2016 to 2020 were used to describe trends in mCPR in Tanzania. Data were collected as a part of routine data collection by the Ministry of Health (MoH) in Tanzania and are publicly available at the Tanzania National Health Portal Data. Note that the mCPR definition used by the MoH most certainly differs from the one used in our outcome evaluation. We calculated the annual mean mCPR along with the 95% confidence interval estimates.

### Results

The data indicated an upward trend in mCPR, but the overlap between the 95% confidence interval estimates indicates no difference between the years (**Figure S19**).

Figure S19 Secondary dataset (Tanzania National Health Portal Data) – Modern contraceptive use among married and unmarried women aged 15-49 years in Tanzania between 2016-2020 using Tanzania National Health definition


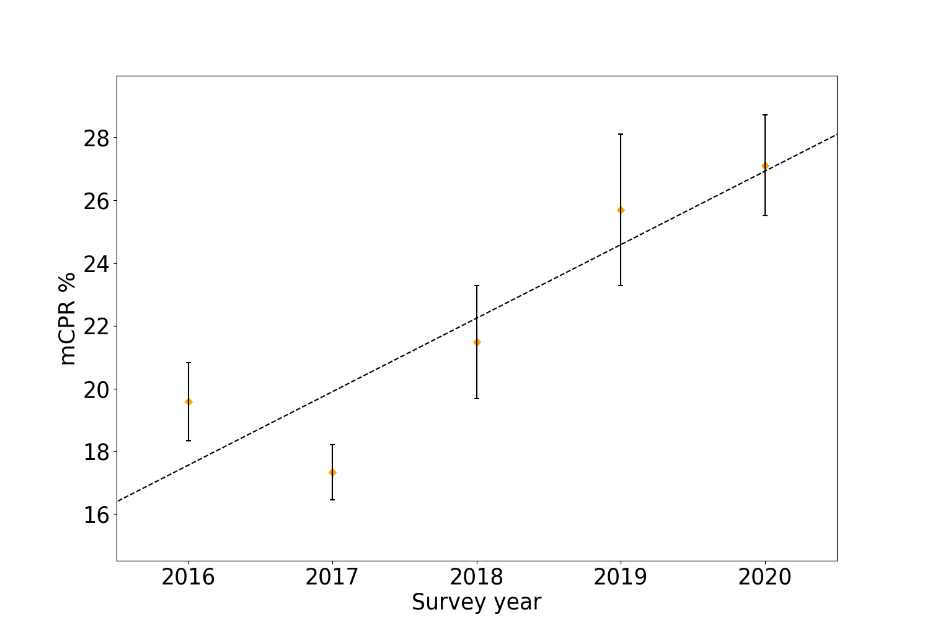


# Methodology details of the Adolescents 360 costing and cost-effectiveness study

Costs were collected from 2016-2020 combining costs from PSI and partner financial systems with multiple rounds of costing from surveys, interviews, and site visits. A360 costs included the costs of PSI and its partners, the government (such as in-kind contributions of commodities and market value of clinical space), and community volunteers (market value of in-kind time provided). Internvetion costs were calculated for the intervention areas included in the outcome evaluation; accounting for a share of costs incurred above these geographies. Design costs were calculated by country and intervention, and amortized over a five-year useful life. A portion of the total design cost per intervention was allocated to the study geographies based on the duration of the intervention and the total number of geographies per intervention. All costs were converted to constant 2020 US dollars.

In order to estimate incremental costs, comparison costs were also estimated. For design, the comparator cost was the cost of the DELTA design methodology, collected in 2017 through interviews and document review. For the internvetion, the comparator cost was the cost to keep contraceptive prevalence constant in the study geographies. Comparator implementation costs were modelled combining measured contraceptive prevalence rates, population projections, and the yearly cost per adolescent family planning user from Guttmacher Institute’s Adding It Up 2019 report (country specific costs from the data annex were used).[26]

Incremental effectiveness was measured in disability-adjusted life-years (DALYs) averted. Adjusted changes in contraceptive prevalence from the outcome evaluation were applied projections of the number of eligible girls in each study geography; a linear change in mCPR was assumed.[27] The projected number of eglible girls was based on sub-national proejctions of females age 15-19 obtained from the U.S. Census Bureau sub-national projections by age and sex to obtain estimates for the relevant subnational geographies (U.S. Census Bureau, 2021), combined marital status data from various sources (DHS and MICS for Nigeria; DHS for Ethiopia) and OE estimes of fecundity and sexual activity.

From this the cumulative number of additional adolescent user years was calculated based on the change in users over time (relative to baseline users). Utilizing outcome evaluation results for contraceptive method mix and country specific coefficients derived from MSI Reproductive Choices Impact 2 model, resulting cumulative incremental unintended pregnancies, maternal deaths, and maternal DALYS averted were calculated.

One-way and multiway sensitivity analyses generated plausible ranges for incremental costs. Sensitivity analysis, conducted in Excel, around effectiveness took into account the 95% confidence intervals for measured change in modern contraceptive use. Probabilistic sensitivity analysis incorporated uncertainty ranges for cost and effectiveness in a Monte Carlo simulation using 10,000 iterations.

This metholological appraoch has important limitations. First, using retrospective surveys and interviews may have generated potential recall error in estimates of leveraged costs and in estimates of how A360 staff split their time between design and other activities. Moreover, reliance on interviews and limited document review to identify costs of DELTA, the comparator design methodology, may have also produced error. Second, **d**esign costs were amortized assuming a five-year useful life of the intervention design. Estimates of design costs were sensitive to the choice of useful life. More research is needed to expand the scarce literature on useful life of intervention design, particularly for an A360-style approach, which is more costly than typical design methodologies. Third, design costs could potentially be underestimated as they included only on-budget expenditures by PSI and its consortium partners. The costing may have missed some costs incurred by other, non-consortium counterparts such as government officials and others who contributed to the design phase. This may have resulted in an underestimate of the true cost of design. Fourth, as inherent to this type of costing exercise results are reliant on allocation rules to distribute unassigned costs to the study geographies. The mostly top-down costing approach relied on PSI and subawardee financial systems, which did not provide full detail on costs specific to the study geographies. We tried to address this limitation by developing appropriate rules to allocate costs to the study geographies. **Fifth, there were constraints in the collection of leveraged costs.** For leveraged costs of the government, we used a bottom-up approach that relied on interviews and site-specific data collection. Although for some inputs we were able to use a census approach, for others we relied on nonrepresentative sampling. Moreover, for some inputs we had incomplete data collection due to inability to contact some personnel, and COVID-19 pandemic related restrictions.

# Adolescents 360 Analysis Plan

## Investigators

LSHTM: Aoife Doyle, Catarina Krug, Christian Bottomley, James Hargreaves, Melissa Neuman

Itad: Stefanie Wallach, Mary Lagaay

BOL: Emily Crawford, Yewande Olanipekun

MMA: Mohammed Mussa

MITU: Saidi Kapiga, Mussa Kelvin Nsanya

Study coordinators

LSHTM: Catarina Krug

BOL: Emily Crawford

MMA: Mohammed Mussa

MITU: Mussa Kelvin Nsanya

Study statisticians

Aoife Doyle, Christian Bottomley, Catarina Krug, Melissa Neuman

## List of Abbreviations and Acronyms

| A360 | Adolescents 360 intervention |
| --- | --- |
| mCPR | Modern contraceptive prevalence rate |
| PSU | Primary sampling unit |
| LGA | Local government area |
| EA | Enumeration area |
| HH | Household |
| PSI | Population Services International |
| MMA | Matasa Matan Arewa |
| SFH | Society for Family Health |
| SNNP | Southern Nations, Nationalities and Peoples |

## Introduction, key objectives and study design

Adolescents 360 (**A360**) is an initiative in the field of adolescent sexual and reproductive health programming, implemented in Nigeria, Ethiopia and Tanzania. The final package of interventions is country-specific. The external evaluation of the A360 intervention comprises a process evaluation, an outcome evaluation, and a cost effectiveness study. We present here the analysis plan for the outcome evaluation of A360, separated in four sections:

1. Overall purpose and scope
2. Nigeria
3. Ethiopia
4. Tanzania

The first section describes the overarching design, primary outcome and operationalisation, and common theory of change and list of secondary outcomes common to all sites. The following sections describe the country-specific research hypothesis and analysis considerations. Tanzania section was added in June 2021, as endline surveys were in standbye due to the COVID-19 pandemic in December 2020, when this analysis plan was first created.

The A360 project is underpinned by a Theory of Change, which elaborates the theoretical causal pathways through which the project intends to affect change. Research objectives are based on the A360 Theory of Change (**Appendix I**).

## Research hypothesis

Our overall hypothesis is that the A360 intervention leads to an increase in modern contraceptive prevalence rate (**mCPR**) over time that is greater than the increase that would have been expected to occur in the absence of the intervention.

Further, we hypothesized that (A) respondents reporting higher levels of exposure to A360 are more likely to use modern contraceptives compared to respondents that report none/low exposure; and (B) respondents living in areas where there was longer period of A360 activity are more likely to use modern contraceptives compared to respondents in areas with a shorter period of activity.

## Primary objectives

The primary goal of the outcome evaluation study is to evaluate the effectiveness of the A360 intervention in increasing **mCPR** among girls aged 15-19 years in study settings in Nigeria, Ethiopia and Tanzania. mCPR will be defined as follows:

$$\frac{\begin{aligned} Number of fecund sexually active 15-19 year old girls reporting \\ use of modern contraceptives at the time of the survey \end{aligned}}{Number of fecund sexually active 15-19 year old girls}$$

**Equation 1. Married or unmarried girls***^[[4]](#footnote-10)^*

Specifically, the primary aim is to quantify the impact of the A360 program on women’s self-reported use of a modern contraceptive method from baseline to endline.

Secondarily, we aim to quantify the impact of the A360 program according to (A) respondent’s degree of self-reported exposure to A360 and (B) implementer reports of duration of A360 activities.^[[5]](#footnote-11)^

## Secondary objectives

Secondary objectives of the outcome evaluation study are to evaluate the effectiveness of the intervention on the following outcomes:

- Proportion of current modern contraceptive users who are using a long-acting or permanent method
- Prevalence of modern contraceptive use in the last 12 months
- Proportion of modern contraceptive users in the past 12 months who used a long-acting or permanent method
- Age at first birth
- Age specific fertility rate
- Unmet need for modern contraception
- Adolescent girls’ access to contraceptive services and products
- Adolescent girls’ agency (self-efficacy) to use modern contraceptives to prevent unintended pregnancies
- Adolescent girls’ knowledge on the use of modern contraceptives to prevent unintended pregnancies
- Adolescent girls’ attitudes towards the use of modern contraceptives to prevent unintended pregnancies
- Adolescent girls’ misconceptions about modern contraceptives
- Community acceptance and social support for adolescent girls to adopt healthy sexual and reproductive health behaviours, including use of modern contraceptives
- Girl’s future aspirations

## Study outline

The study design in each setting is summarised in **Appendix III**. A cluster sampling design was used in both countries. Girl is at the lowest level of the hierarchy (level1). There are one or more girls within households (level2) and several household per primary sampling unit (**PSU**). In each country we used the smallest available admistrative unit as the PSU (**Figure 20**), which is enumeration area (**EA**) in Nigeria, Kebele in Ethiopia, or Street in Tanzania (level3). Finally, the highest level of the hierarchy (level4) corresponds to LGA in Nigeria, and Wareda in Ethiopia, and Ward in Tanzania.

The PSU is the cluster in our cluster sampling and is larger than the unit of concern^[[6]](#footnote-12)^, the 15-19 year old girl.

Sample size calculations for each setting are shown in **Appendix IV**. Further details are given in the protocol publication [28].

Figure S20 Hierarchical levels, by A360 outcome evaluation setting

Nig, Nigeria, Eth, Ethiopia, Tz, Tanzania, LGA, local government area, EA, enumeration area, Pop, population

## Study populations

Our first target population were adolescent girls aged 15 to 19 years. However, within this study population our primary outcome and some of our secondary outcomes will only be evaluated in girls who report that they have been sexually active in the 12 months prior to the survey (see **Appendix I and II** for details).

To measure community acceptance and social support for adolescent girls to adopt healthy sexual and reproductive health behaviours our target population were adults in the household who may be most influential to a girl’s decision making. Therefore, in households were the girl interviewed was married^[[7]](#footnote-13)^ we invited the husband/ male partner to participate. The girl’s permission was sought to interview her husband/ male partner. In households where the girl interviewed was unmarried^[[8]](#footnote-14)^, we asked her to nominate a co-habiting adult (age 20+ years) whose views were most likely to influence her decision-making with regards to sexual health and family planning.

## Inclusion/exclusion criteria

A360 targets different subpopulations of adolescent girls in the three countries (**Box 1**). Eligibility criteria does not include any criteria related to contact with or exposure to elements of the A360 programme.

Box 1 Study eligibility criteria

Inclusion criteria

- Adolescent girls aged 15 to 19 years
- Unmarried (Southern Nigeria)
- Married or living as married (Ethiopia and Northern Nigeria only)
- Living, at the time of the survey, in the study sites
- Voluntarily provides informed consent
- If unmarried and under 18 years of age, guardian/parent voluntarily provides informed consent

Exclusion critieria

- There were no specific exclusion criteria

## Data collection tools

Baseline and endline household surveys were carried out in mid-2017, and late-2020, respectively. The exception was Tanzania, for which endline surveys happened in mid-2021. Individual, informed consent, and where required parental/guardian consent, was obtained from all participants before conducting the interviews (more details in the country-specific sections).

The questionnaires were adapted from respective country Demographic and Health Survey (**DHS**) [29-31] and FP2020 survey instruments. At baseline, cross-sectional population based surveys were administered face-to-face using tablets by female interviewers aged between 18 and 26 years. At endline, cross-sectional population based surveys were administered partially face-to-face and partially by phone (see further details on **Appendix V**).

The questionnaire had five components:

1. Socio-demographic characteristics – age, religion, education, household amenities;
2. Sexuality and fertility characteristics – age at first sexual intercourse, timing of last sexual intercourse, pregnancy and childbearing experiences and intentions;
3. Contraceptive characteristics – knowledge and use of contraception, heard about modern contraception and sources of information on contraception, approval of married/unmarried couples using a modern contraceptive method to avoid or delay pregnancy, where obtained method, knowledge of the benefits of contraception, misconceptions about contraception, self-efficacy to use modern contraception, reasons for not using;
4. Exposure to the A360 intervention
5. Girl’s future aspirations

The survey tools were translated into the local languages, pre-tested, and adjusted accordingly. Enumerators received training on the project aims, the content of the surveys, community entry, data collection, and ethics over 5 days. All studies were approved by the London School of Hygiene and Tropical Medicine ethics committee and by the local in-country ethical entity.

## Outline of statistical analysis

### Unit of statistical analysis

The 15-19 year old girl will be the unit of statistical analysis for most analysis, except for the main analysis in Ethiopia and Tanzania, where Kebele and Street, respectively, will be the unit of statistical analysis, to increase power.

### Definitions and data manipulations

This section deals with data manipulation of key variables for analysis. Outcomes are presented in order of their importance in relation to the project aims.

#### General derived variables

**Appendix I** and **II** detail variable categorization from questionnaire.

In general, for those variables created based on two or more questions, scale or index scores will be calculated using an unweighted procedure. This will be done by simply summing raw item scores [32, 33]. Greater sums will generally be more desirable than lower sums.

##### Socioeconomic index

Socioeconomic status will be derived from a series of questions using the ‘Nigeria Equity Tool’ [14], ‘Ethiopia Equity Tool’ [34], and ‘Tanzania Equity Tool’ [35] for each respective country. Details are given in **Appendix VI**.

In summary, if the population of interest is predominantly urban, results are compared to other urban dwellers for interpretation, by generating urban wealth quintiles. If the population of interest live in rural areas, or a mix of urban and rural areas, results are compared to the national results to understand how relatively wealthy or poor they are, in comparison to the whole country, by calculating national wealth quintiles. Wealth quintiles range from poorest (1^st^ and 2^nd^ quintiles) to richest (4^th^ and 5^th^ quintiles).

##### Exposure to the A360 intervention^[[9]](#footnote-15)^

We will use a series of questions to rank individuals by their level of engagement with the A360 interventions that are available in the place where they live. Each country has five measures of exposure to the A360 programme, as listed in country-specific sections. Degree of self-reported exposure will be defined as follows:

1. **No exposure**: Participants that have never heard about the A360 programme;
2. **Low exposure**: Participants that have heard about the A360 programme and/or recognize the logo and/or have heard about particular features of the A360 programme (classes in Southern Nigeria or the goal card in Ethiopia) but have never attended/participated in the activities;
3. **High exposure**: Participants that have attended/participated in the A360 activities (participated in classes/counselling in Nigeria; or has a goal card if in Ethiopia).

### Approach to Missing data

We will conduct a complete case analysis i.e. consider only observations for which there are no missing values for the outcome or any of the predictors. However, if there are a large number of missing values for key variables then we will also conduct a sensitivity analysis where we impute values for the key variable(s).

### Descriptive analysis

#### Recruitment and data completeness

Structure and household listing (in Nigeria both intervention and comparison sites) at baseline and endline will be described. Contact and consent rates will also be described, as well as main reason for non-contact and for non-consent. These rates will be compared with A360 baseline survey data and also with other adolescent sexual and reproductive health surveys [36]. A flow chart as shown in **Figure 21** will present the process of participant recruitment, consent and data completeness. A Table (as shown in **Appendix VII,** Response rates) will specify the reasons for non-response at baseline and endline for both girls and co-habiting adults.

Figure S21 Participant flow diagram

PSU, Primary sampling unit, HH, Household

*Information obtained from individual living the structure/household or from a neighbour.

### Hierarchical or multilevel data

We will describe hierarchical/multilevel data as shown in **Table S4**.

Table S4 Structure of the dataset used for investigating the effect of Adolescents 360 intervention on modern contraceptive use

| Level | Number of units | Replication within higher level ^1^ | |
| --- | --- | --- | --- |
|  |  | Mean | Range |
| LGA / Woreda |  | - | - |
| EA / Kebele |  |  |  |
| Households |  |  |  |
| 15-19 year old girls |  |  |  |

^1^ Number of observations per cluster. For instance, number of 15-19 year old girls per household, or number of households per EA / Kebele.

### Demographics and characteristics

Eligible participants will be described as shown in **Appendix VII** (Demographic variables). Comparability between samples at baseline and endline (and in Nigeria between intervention and comparison sites) will be evaluated in terms of age, religion, school enrolment, etc [36]. We will also describe them by sexuality, fertility and fertility preferences, as shown in **Appendix VII** (Fertility).

The degree of self-reported exposure to A360 (percentages) at endline will be described by demographic variables, as shown in **Appendix VII** (Exposure to A360).

### Analysis of the primary outcome

We will describe prevalence of contraceptive use (with confidence intervals), as shown in **Appendix VII** (Contraceptive use section), by

- Demographic variables**;**
- Baseline and endline (and comparison and intervention areas in Nigeria);
- Exposure characteristics.

The primary outcome will then be assessed in the following ways:

1. The impact of the A360 program from baseline to endline (primary analysis);
2. The impact of the A360 program according to level of exposure to the programme (secondary analysis); Exposure will be defined in two ways:

B1) The degree of self-reported exposure to A360;

B2) The duration of A360 activities in each intervention area.

#### A The impact of the A360 program from baseline to endline

For our primary analysis, we will compare mCPR over time among young women meeting eligibility criteria. In this approach, all women living in the intervention-area will be considered ‘exposed’ to the program regardless of whether or not they received the intervention [37]. We will therefore be estimating the population-level effect of the intervention.

We will use regression models to assess the strength of association between the A360 interventions (predictor: time before, 0, vs. time after the intervention, 1) and the use of modern contraception (outcome). The analysis will vary by country, and is described in detail in each country section.

#### B The impact of the A360 program according to level of exposure

It will be critical to inform our interpretation of the outcome data to show data on the delivery of the implementation in the allocated A360 intervention areas.

It is not possible to know exactly who was exposed to the A360 intervention, among the endline respondents. Nevertheless, we can estimate that exposure based on B1) The degree of self-reported exposure to A360; and B2) The duration of A360 activities in each intervention area.

##### B1) Degree of self-reported exposure to A360

After describing the degree of self-reported exposure to A360 (percentages) at endline, by demographic and by outcome variables, we will use regression models to assess the strength of association between the level of engagement with the degree of self-reported exposure (0 no exposure, 1 low exposure, 2 high exposure) and the use of modern contraception (outcome).

We will use modified Poisson regression models with robust standard errors (at the EA or kebele level, depending on the country), as follows [11]:

Y_i_ ~ Poisson(λ_i_)

log(λ_i_) = β_0_ + β_1_ Exposure_i_

where β_0_ is the outcome at no exposure; β_1_ reflects the overall effect of exposure. The use of robust standard errors in this model allows not only to account for clustering of observations at the EA (Nigeria) or kebele (Ethiopia) level, and for the conservative confidence intervals that often result from Poisson regression models [37]. We will adjust for the following demographic variables: age, education level, parity, religion and wealth quintile [13].

**Appendix VII** (Final regression models) shows how we will present the model results. The analysis will be restricted to endline data, and, in Nigeria, to intervention areas only.

##### B2) Duration of A360 activities

We hypothesized that the relationship between A360 programme and mCPR could change by the duration of A360 activities in each geographical area of interest. Therefore, we will check interactions between duration of A360 activities at the EA (Nigeria), kebele (Ethiopia) or district (Tanzania) and time before (‘0’), and after the intervention (‘1’). To define the duration of A360 activities per site, we will use data collected by Population Services International (**PSI**), the institution implementing A360 in the two countries. Their data reports the number of girls who were exposed to A360 activities, per month and year, and per geographical area. PSI reporting systems were set up prior to implementation and the monitoring and evaluation data are understood to be an accurate reflection of implementation.

Data structure varied by country, as shown on **Table S5**.

Table S5 Structure of the PSI monitoring data, used to define duration of A360 activities

| **Country** | **Level 1 (lowest)** | **Level 2** | **Level 3** | **Level 4** | **Level 5** | **Level 6** | **Level 6** |
| --- | --- | --- | --- | --- | --- | --- | --- |
| Nigeria | Girl | Facility | Community | **Ward (EA)** | LGA | State | Region |
| Ethiopia | Girl | Facility | **Kebele** | Woreda | District | - | - |
| Tanzania | Facility | District | Region | - | - | - | - |

Note: This table still needs further clarification for PSI before being finalised

To define duration of activities, in Nigeria and Ethiopia we considered both the months of A360 activities (months where number of girls was greater than 0) per EA (Nigeria), or per Kebele (Ethiopia), and descriptive information given by PSI. In Tanzania, since activities were event-based, we considered not only the information given by PSI and the sum of girls reached per month per District (Tanzania), but also the number of events per month per District. Data is presented in each country section. From now on we will refer to PSI data as *PSI monitoring data*.

### Degree of self-reported length of time living in the community

This will be a sensitivity analysis, in which any girls who report having spent more than 3 months out of the area of A360 intervention in the past 12 months, will be excluded from the analysis. We will then conduct the same analysis described in section 5A (the impact of the A360 program from baseline to endline) and see if this leads to any changes in the statistical conclusions and in the point estimates. We hypothesise that we will observe a greater intervention impact when only keeping individuals that did not leave the intervention area.

This variable will be assessed through the following questions:

- In the last 12 months, have you stayed/lived in a place other than this LGA (Nig) / Woreda (Eth) for one month or more?
- In total approximately how long have you spent outside this LGA (Nig) / Woreda (Eth) in the last 12 months?

Absence for at least three months in the past 12 months will be used as a proxy for absence in the previous 24 months, which is the average time from the start of the intervention until endline surveys.

## Analysis of secondary outcomes

All secondary outcomes which are binary, and measured at both baseline and endline will be evaluated as described for the main outcome:

1. The impact of the A360 program from baseline to endline, as specified in each country-specific section;
2. The impact of the A360 program at endline among those reporting exposure to the program, as specified in overall study design.

For those outcomes which were only measured at endline, we will only analyse them as described in option (B) above, which only uses endline data. In Nigeria, we will also compare intervention and comparison areas, at endline, adjusting for predefined confounders.

For those outcomes which are not binary, we will use linear regression models as follows:

Y_i_ = β_0_ + β_1_PredictorA

where Y_i_ is the predicted outcome for the i^th^ girl; β_0_ is the intercept; β_1_ is the regression coefficient for predictor A.

Some of the outcomes used to measure community acceptance and social support for adolescent girls to adopt healthy sexual and reproductive health behaviours will use the cohabitant adult datasets (see **Appendix I** and **II** for details).

## Stata commands

General Stata commands are described in **Appendix IX**.

## Potential limitations

### General to all settings

#### Information bias

Information bias may occur due to misclassification (categorical variable) of mCPR. Since both the use of contraceptives and sexual activity (particularly among unmarried women) are sensitive topics, girls may report that they are not contraceptive users or that they are not sexually active, even though they are. To decrease bias in reporting, all interviews were conducted in privacy, and away from husband and other adults, as much as possible.

#### Selection bias

The girl’s permission was sought to interview an adult whose views were most likely to influence her decision-making with regards to sexual health and family planning. Consequently, selection bias of the population of adults may have happened, as the adult participants may be more supportive of contraceptive use compared to the overall population of influencial adults.

#### The issue of Multiple comparisons

Having too many secondary outcomes may lead to a problem of ‘multiple comparisons’ [37]. To deal with it we will focus on effect sizes and consistency within the findings and less on p-values.

## Analysis steps

**Initially (by 1^st^ Feb), we will describe:**

- Participant flow by baseline/endline and, in Nigeria, comparison/intervention areas (by matched pair)
- Multilevel/hierarchical data
- Population at baseline and endline and, in Nigeria, comparison/intervention areas (by matched pair), by demographic factors, sexuality, fertility and fertility preferences
- Primary outcomes by baseline/endline and, in Nigeria, comparison/intervention areas (by matched pair)
- Primary outcomes: Main analysis, using robust standard errors

**Later on (after 1^st^ Feb), we will also:**

- Describe the degree of reported exposure to A360 interventions by demographic factors
- Primary outcomes: The impact of the A360 program according to level of exposure
- Secondary outcomes by baseline/endline and, in Nigeria, comparison/intervention areas (by matched pair)
- Secondary outcomes:
  - Before and after analysis in Ethiopia, and before-after with comparison group in Nigeria
  - Analysis of effect of degree of reported exposure to A360

## Methodology in Nigeria

### The A360 intervention

#### Ogun

In Nigeria, 9ja Girls provided branded safe spaces in public health clinics for girls. Walk-in 1-1 counselling was provided alongside Life, Love, Health classes. The curriculum features vocational skills, future-planning exercises, and discussions about love, sex and dating. The aim was to help girls see contraception as a tool to reach their goals. The program was delivered through a youth-friendly provider network, leveraging partnerships with the Ministry of Health to train health service providers. 9ja Girls was implemented in seven regions in Southern Nigeria: Lagos, Osun, Ogun, Oyo, Edo, Delta and Akwa Ibom [38].

The A360 package was delivered at the level of local government areas (**LGA**; intervention unit).

#### Nasarawa

In Northern Nigeria, Matasa Matan Arewa (**MMA**) targets married adolescent girls and their husbands using maternal and child health as an entry point. Male Interpersonal Communicators discuss contraception with husbands, using the health of the baby and mother as an entry point to encourage husbands to refer their adolescent wives to a female mentor or to a clinic for counselling. Female mentors also mobilize married adolescent girls directly. Girls are then mentored through four Love, Life and Family classes in a setting identified by them, and receive one-on-one counselling with a provider and a vocational skills class. MMA also works with religious leaders and communities, to emphasize the benefits of child spacing. MMA was implemented in three regions in Northen Nigeria: Federal Capital Territory, Nasarawa and Kaduna [39].

### Research hypothesis

In Nigeria, the primary hypothesis is that mCPR in intervention communities at endline is higher than mCPR in comparison communities, after adjustment for baseline differences and confounding factors.

### Study design

In Nigeria, a quasi-experimental design with comparison group was used. It is quasi-experiemental because random assignment of intervention and comparison sites was not possible. We used before-and-after^[[10]](#footnote-16)^ population-based surveys, one survey happening in late 2017 and the other in late 2020.

#### Study unit inclusion criteria and selection

##### State

The intervention will be evaluated in Ogun (Southern Nigeria) and Nasawara (Northern Nigeria). Study states were selected by the Society for Family Health (**SFH**). The selected states were chosen because of the absence of other adolescent focused sexual and reproductive health activities and because of SFH’s previous experience working in these states.

##### Local government area - allocation to intervention and comparison arms

Study LGAs were selected by SFH in collaboration with the state Ministry of Health and local government officials. Within a pair, allocation of an LGA to the intervention or comparison arm of the outcome evaluation study was purposively selected by SFH in collaboration with the state Ministry of Health and local government officials. Intervention LGAs were selected first, and then paired LGAs recommended by SFH. The LGAs were selected from among those where there was no security concerns, and comparison-intervention pairs were selected to be similar with respect to some or all of the following criteria:

- Population density
- Estimated modern contraceptive prevalence rate (mCPR) among 15 to 49 year olds (DHIS2, 2016)
- Number of health facilities
- Presence of World Bank support for Maternal and Child Health activities

In Ogun, the evaluation was conducted in two LGAs (1 intervention and 1 comparison). In Nasawara, four LGAs consisting of two similar pairs have been selected for evaluation. Two of these received the intervention (1 in each pair) and two did not—i.e., they act as comparisons. The size of selected LGAs is presented in **Table S6**.

Table S6 Comparability of outcome evaluation study intervention and comparison LGAs in Ogun and Nasarawa states

| **LGA** | **Total pop. (2006)^1^** | **Estimated number of household^2^** | **Pop. 15-19 year old females^1^** | **Estimated mcpr (15-49 years)^3^** | **Pop. Density (/km^2^)** | **No. of EAs^1^** | **Ratio of health facilities public:private^4^** | **Health facilities^5^** | |
| --- | --- | --- | --- | --- | --- | --- | --- | --- | --- |
|  |  |  |  |  |  |  |  | **Public^4^** | **Private^4^** |
| Ogun state | | | | | | | | | |
| Ado-Ota Oda (I) | 527,242 | 131,811 | 24,100 | 2·2 | 600·5 | 2,253 | 0·3 | 8 | 28 |
| Shagamu (C) | 255,885 | 51,177 | 12,443 | 2·5 | 416·8 | 1,117 | 0·3 | 24 | 80 |
| Nasarawa State - pair 1 | | | | | | | | | |
| Doma (I) | 174,046 | 34,806 | 8,702 | 6·3 | 59·5 | 993 | 1·3 | 56 | |
| Toto (C) | 148,452 | 29,690 | 7,422 | 8·3 | 47·6 | 513 | 4·8 | 76 | |
| Nasarawa State - pair 2 | | | | | | | | | |
| Karu (I) | 256,166 | 51,233 | 12,808 | 34·6 | 95·2 | 1209 | 1·9 | 197 | |
| Nasarawa (C) | 236,665 | 47,333 | 11,833 | 11·8 | 38·1 | 1116 | 1·8 | 84 | |

LGA, local government area, HH, household, mCPR, modern contraceptive use, EA, enumeration area, I, Intervention arm, C, Comparison arm

^1^Estimated population (pop.) in 2013 based on 2006 Nigeria Population Census;

^2^4/household in Ogun and 5/household in Nasarawa;

^3^HMIS Nigeria (2015);

^4^2011 MEASURE Evaluation Health Facility Mapping;

^5^DHIS2 (2016)

##### Population A: 15-19 year old girls

In Ogun, the study population were unmarried girls aged 15–19 years. In Nasawara, the study population were married girls (or living as married) aged 15–19 years.

EAs within LGAs (intervention allocation units) were randomly sampled at baseline (through simple random sample), and the same EAs were included at endline, where possible. A simple random sample of 710 enumeration areas (EA) in Ogun and 1,150 EAs in Nasarawa was obtained. At endline, some EAs were replaced for security reasons. Moreover, the population of eligible girls decreased over time, so some additional EAs were visited, to be able to sample the target sample of girls.

In Nasawara state, Nasawara LGA (comparison LGA) shares a border with Karu and Doma (intervention LGAs; see **Figure 25**). Therefore, to reduce spill-over due to girls travelling to work or school across LGA boundaries, a “buffer zone” were created such that EAs within localities (larger geographic areas containing many EAs) in Nasarawa LGA that border either Karu or Doma were excluded from the sampling frame.

Within each EA we sampled (simple random sample) clusters of approximately 100 households. If a selected EA contained fewer than 100 households, then we continued the data collection in an adjoining EA until 100 households were selected. The questionnaire was administered to all eligible unmarried girls aged 15 to 19 years in the selected households in Ogun and all eligible married girls aged 15 to 19 years in Nasarawa. Although the design means that it is possible that in each site the same households and individuals may be included in the baseline and endline surveys, no attempt was made to trace individuals or households from baseline to endline.

##### Population B: Cohabitating adult

We interviewed a cohabitating adult in 20% of the study households in Ogun and the husband/male partner of 10% of the married girls interviewed in Nasarawa.

### A360 activities in Local government areas selected

#### According to PSI monitoring data for A360

Figure S22 Number of girls reached per month and year in Ado-Odo/Ota LGA, Ogun State


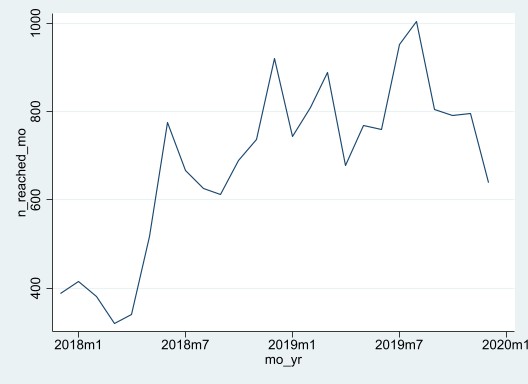


Figure S23 Number of girls reached per month and year in Doma LGA (left image) and Karu LGA (right image), Nasarawa State


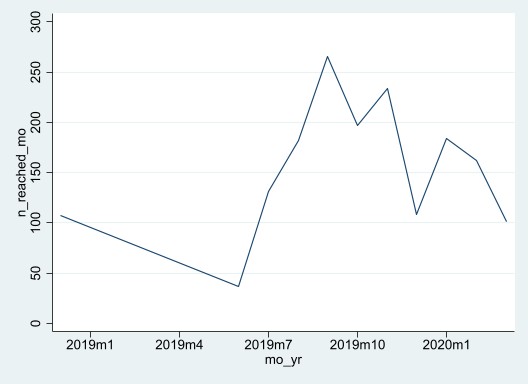

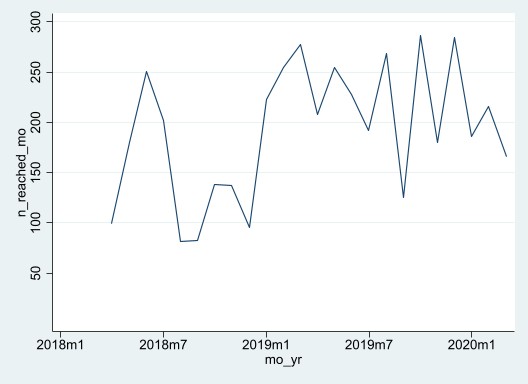


#### According to PSI team for A360

Table S7 Nigeria: Expected exposure to the A360 intervention, according to PSI team

| **Sites (LGA)** | **Type of activity** | **Communities of** | **A360 implementation starts in OE Sites** |
| --- | --- | --- | --- |
| Ogun: Ado-Odo Ota | In clinic | PHC in Otun Ota | Nov 17 |
|  | In clinic | Private sector facility (Ariset Medical Center) in Sango* | Nov 17 |
|  | In clinic | State hospital in Ota | Nov 17 |
|  | Reach out | One community of name unknown | Jun 18 |
|  | Reach out | Igberen, Iyana Iyesi and Ijoko | Nov 18 |
|  | Reach out | Alapoti, Atan, Iju Itele, Owode and Sango | Jul 19 |
| Nasarawa: Doma | In clinic | Arumangye PHC | Jun 19 |
|  | Reach out | Okpatta, Idadu, Effugibringo, Angwan Kwasu and Rukubi | Sep 19 |
| Nasarawa: Karu | In clinic | Mararaba PHC | Apr 18 |
|  | Reach out | Aso pada, Gitata, Masaka and Rugan Julifrom | Nov 18 |
|  | Reach out | Gurku and Gunduma | Feb 19 |
|  | Reach out | Zheun | Mar 19 |

SFH has moved out of Ariset Medical Center based on request from the owner at unknown date. Replacement facility selection ongoing in Nov 2020

Key info obtained from PSI team: Most of new adopters were reached before 2019 (45%), particularly in Ogun. In Doma, Nasarawa, the proportion of individuals reached that were aged 19 was lower (15%) than in Karu (35%). Moreover, Doma LGA had about one third as much activity as Karu LGA.

#### According to both sources of information

Table S8 Nigeria: Expected exposure to the A360 intervention, according to PSI monitoring data and PSI team info combined together

| **Sites (LGA)** | **Start date according to PSI monitoring data** | **Earliest start date by PSI team** | **A360 start date considered** | **Duration of A360 activities (Endline surveys**  **in Nov/Dec 2020)** |
| --- | --- | --- | --- | --- |
| Ogun: Ado-Odo Ota | Dec 17 | Nov 17 | Dec 17 | 36 months |
| Nasarawa: Doma | Dec 18 | Jun 19 | Jun 19 | 18 months |
| Nasarawa: Karu | Apr 18 | Apr 18 | Apr 18 | 31 months |

Note: In Doma, PSI team said interventions started in Jun 19, but the monitoring data says Dec 18. We checked these dates with PSI team in Feb 2021

Figure S24 Map of Ogun State showing LGAs by their boundaries. Intervention LGAs in blue and comparison LGAs in red.


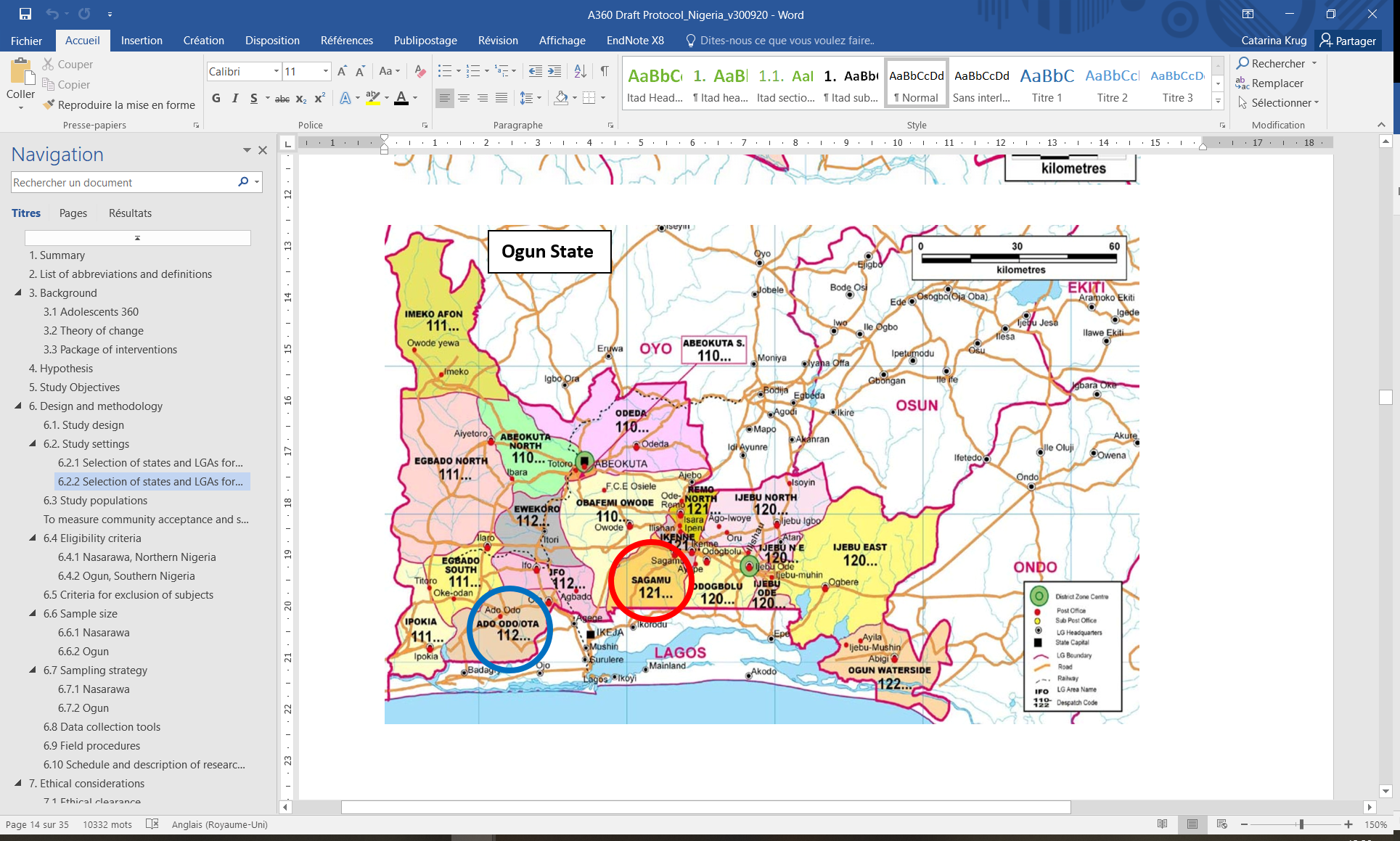


Figure S25 Maps of Nasarawa State showing LGAs by their boundaries. Intervention LGAs in blue and comparison LGAs in red.


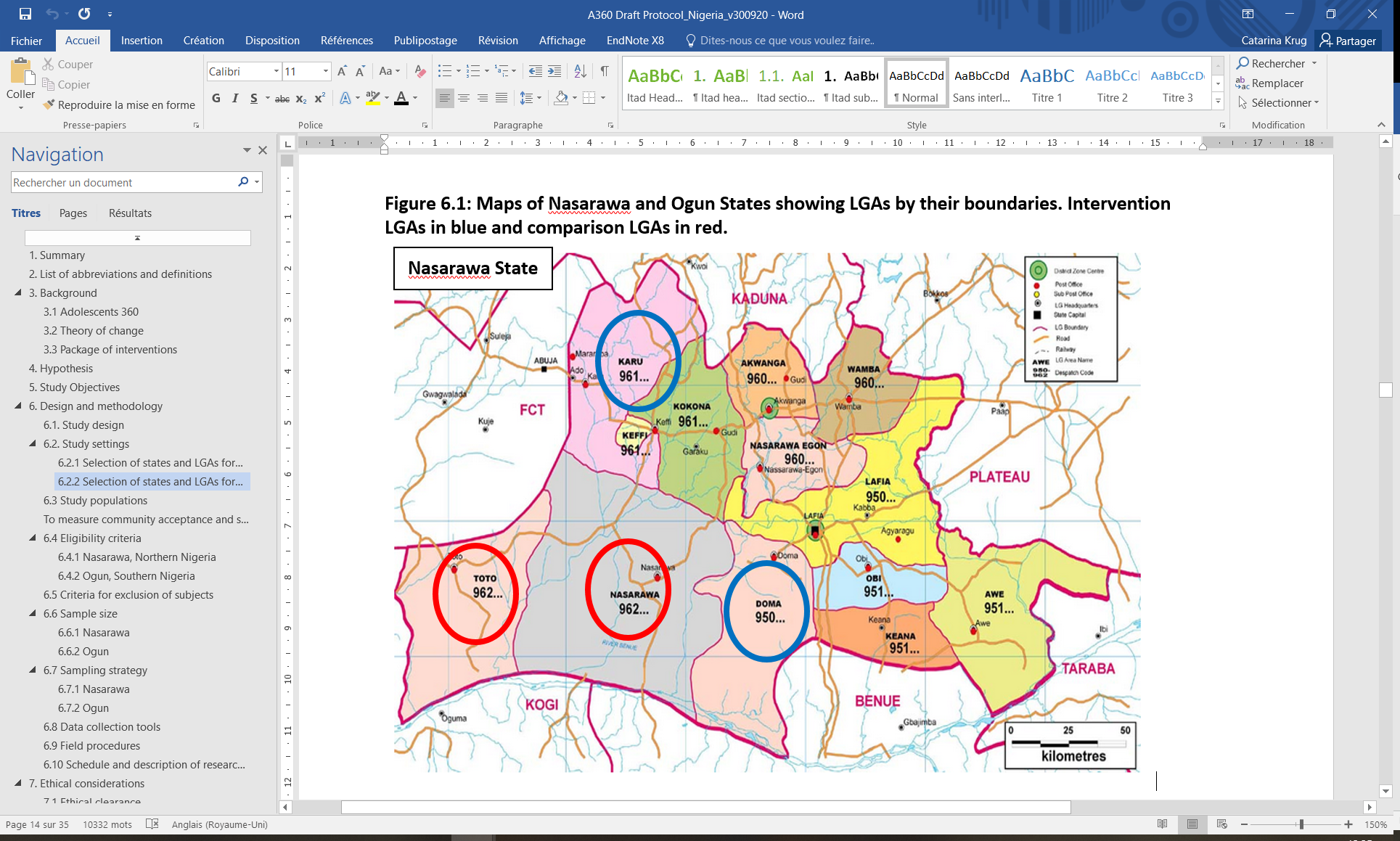


### Baseline comparability between pairs of Local Government Area

Quasi-experimental studies should report a balance table showing that the comparison group have the same characteristics as the intervention group, apart from treatment. However, as stated by White and Raitzer [40], balance tables can only check balance on observable characteristics, so there may still be bias from lack of balance on characteristics that have not been observed.

We will compare baseline key sociodemographic characteristics between intervention-comparison pairs of LGAs to assess balance at baseline as shown on **Table S9**.

Table S9 Nigeria: Adolescents 360 outcome evaluation baseline data

| **LGA** | **mCPR** | **Median age 1^st^ sex** | **Unmet need for family planning** | **% Secondary education** |
| --- | --- | --- | --- | --- |
| **Ogun** |  |  |  |  |
| Ado-Ota Oda (I) | 44·7% ( 41·1, 48·3) | 16 | 33·5% | 89·8% |
| Shagamu (C) | 49·8 (46·5, 53·1) | 16 | 31·7% | 90·6% |
| **Nasarawa- pair 1** |  |  |  |  |
| Doma (I) | 7·6 (5·5, 10·3) | 14 | 19·1% | 24·.6% |
| Toto (C ) | 12·8 (9·5, 17·2) | 15 | 27·5% | 41·8% |
| **Nasarawa- pair 2** |  |  |  |  |
| Karu (I) | 21·3 (18·5, 24·5) | 16 | 22·2% | 56·9% |
| Nasarawa (C ) | 13·0 (10·6, 15·8) | 15 | 20·0% | 42·4% |

LGA, local government area, I, Intervention arm, C, Comparison arm

### Regression framework

As mentioned above, comparison and intervention LGAs were selected in pairs, therefore the (descriptive and regression) analysis will be conducted separately for each matched pair. Nevertheless, for Nasarawa state, the main result will be the effect of time (endline, time ‘1’, compared to baseline, time ‘0’) on mCPR over all intervention (A360 ‘1’) and comparison areas (A360 ‘0’), i.e. the four LGAs will be analysed together in one model. The reason for this is that the study was powered to detect an impact overall for all four LGAs so it may be underpowered to observe an effect if separating LGA pairs.

#### The impact of the A360 program from baseline to endline

Two data sets will be used per State, one with baseline data from the intervention and comparison sites, and the other with endline data from the two sites. Datasets will be appended, and a dummy variable (e.g. time) will identify whether the survey was conducted at baseline or at endline. Another dummy variable will identify whether the respondents are from the intervention or the comparison site (e.g. A360).

Logistic regression is the most widely used model for binary data, expressing estimated of effect as odds ratios [37]. However, risk ratios are generally recommended when summarizing effect size in studies with common outcomes [41]. Poisson regression can be used in a similar way as logistic regression, with time-at-risk being specified as ‘1’, to directly estimate risk ratios [37, 42].

We will use modified Poisson regression models with robust standard errors (at the EA level), as follows [11]:

Y_i_ ~ Poisson(λ_i_)

log(λ_i_) = β_0_ + β_1_Time_i_ + β_2_A360_i_ + β_3_Time_i_ × A360_i_

where β_0_ is the outcome at baseline (time 0) in a comparison area (A360 0); exp(β_1_) is the risk ratio for contraceptive use between endline (time ‘1’) and baseline (time ‘0’), exp(β_2_) is the risk ratio for contraceptive use between being in an intervention area (A360 ‘1’) or in a comparison area (A360 ‘0’), and exp(β_3_) is the effect of A360 beyond the time effect [12]. The use of robust standard errors in this model allows us to account for clustering of observations at the EA level, and for the conservative confidence intervals that often result from Poisson regression models [37]. In Nasarawa State, a variable “pair” will identify paired comparison and intervention sites.

Participants were not randomly assigned to the intervention, thus differences in the outcomes of interest may result from some characteristics related to the study sites. We will adjust for the following demographic variables, which are associated with contraceptive use according to the literature: age, education level, parity, religion and wealth quintile [13].

**Appendix VI** (Final regression models) shows how we will present the model results.

#### The impact of the A360 program according to level of exposure

##### The impact of the A360 program at endline among those reporting exposure to the program^[[11]](#footnote-17)^

Analysis will be done as described in overall study design. In Nigeria, we will also describe level of engagement with A360 in comparison areas, where the intervention did not take place, to understand any bias in reporting, as shown on **Table S10**.

Table S10 Definition of exposure to A360 interventions at endline in Nigeria

|  | **Self-Reported Exposure** | | |
| --- | --- | --- | --- |
|  | **Categorization** | | **Degree if 1** |
| Southern Nigeria (5 interventions) | 1 | 0 |  |
| Heard about 9ja girls (no/yes) | Answers yes | Answers no | Low |
| Recognizes 9ja girls logo (no/yes) | Answers yes | Answers no | Low |
| Heard about life, love & health classes (no/yes) | Answers yes | Answers no | Low |
| Participated in LLH classes (no/yes) | Answers yes | Answers no | High |
| Received one or more one-on-one counselling from a trained provider (0, 1, 2-5 or >5 times) | 1-5 or >5 times | 0 times | High |
|  |  |  |  |
| Northern Nigeria (5 interventions) |  |  |  |
| Heard about matasa matan arewa (MMA) (no/yes) | Answers yes | Answers no | Low |
| Recognizes mma logo (no/yes) | Answers yes | Answers no | Low |
| Participated in life family health (LFH) classes (no/yes) | Answers yes | Answers no | High |
| Participated in health, nutrition and interpersonal skills classes (no/yes) | Answers yes | Answers no | High |
| Received one or more one-on-one counselling from a trained provider (0, 1, 2-5 or >5 times) | 1-5 or >5 times | 0 times | High |

Table S11 Degree of self-reported exposure to A360 (percentages) at endline, by comparison and intervention areas

|  | Endline (n=) | |
| --- | --- | --- |
|  | Comparison (n=) | Intervention (n=) |
| Degree of Self-Reported Exposure (percentages) |  |  |
| None (no exposure) |  |  |
| Low (Heard about A360) |  |  |
| High (Participated in A360) |  |  |

1 Analysis will be conducted separately for eached matched pair. Although we do not expect any exposure to A360 in comparison areas, we will also describe level of engagement with A360 in those sites to understand and maybe correct any bias in reporting

##### Duration of A360 activities

We hypothesized that the relationship between A360 programme and mCPR could change by the duration of A360 activities.

In this analysis, we will include an ecologic predictor, i.e. the duration of A360 activities in each EA, to estimate its contextual effect on estimates of mCPR at individual-level [43]. To do this, the following interactions will be added to the generalized mixed model defined in section A1:

- Time x Duration: 2-way interaction between Time (before, 0, after, 1) and duration of A360 activities
- A360 x Duration: 2-way interaction between A360 (comparison site, 0, intervention site, 1) and duration of A360 activities
- Time x A360 x Duration: 3-way interaction between Time, the A360, and duration of A360 activities

Interaction terms will be considered significant whenever the F-test yields a P-value <0.05. If the 3-way interaction is deemed significant, then all 2-way and main terms will be kept in the model to allow interpretation.

##### Degree of self-reported length of time living in the community

As shown in overall study design.

### Sampling weights

An equal number of households was selected from each EA, and each EA has approximately 100 households. Therefore, the probability of selection did not differ between households. Also, since all eligible girls were selected per household, then the probability of selection for any given girl from each household is 1. Therefore, there is no need to adjust for sampling weights.

### Potential limitations – Nigeria specific

#### Lack of randomisation

Randomisation was not possible and so a major challenge for analysis is to adjust for confounding and/or secular trends in our analysis. In Nigeria, we will obtain our estimate of likely change in mCPR in the absence of A360 from comparison communties, and use this in a difference in difference analysis.

The difference in difference analysis assumes a common trend in the outcome in both the intervention and comparison areas. Even if the two areas differ in a number of characteristics, the analysis is valid provided the common trend assumption is upheld. We will measure potential confounders at baseline and endline and adjust our analysis for any compositional changes over time in these confounders.

If there is an association between the level of exposure to the A360 and mCPR, even when adjusting for confounders, then this will increase our study’s validity.

#### Contamination

In Nigeria, comparison sites are meant to be the same as the intervention sites, except that the former is not exposed to A360, but it is possible that other programs in the comparison area affect the outcomes of interest – we will describe other past or ongoing interventions in the areas of study.

#### Spillover effects

In Nigeria, it is also possible that target populations move from comparison to intervention areas or vice-versa – we will record whether girls interviewed at endline lived on site within the last 12 months, or if she was out, and for how long.

In Ogun State, Ado-Oda/Ota and Shagamu are separated geographically by Ifo LGA and therefore we will assume that movement of residents between intervention and control LGA will be minimal (**Figure S24**).

## Methodology in Ethiopia

### The A360 intervention

In Ethiopia, Smart Start uses financial planning as an entry point to discuss contraception with newly married couples. It leverages the nationwide Health Extension Worker network, supported by a Smart Start team, and the Women’s Development Army. Health Extension Workers and Smart Start Navigators are trained to host conversations and provide services in an approachable way for rural, married adolescent girls and their husbands, using a visual discussion guide. Smart Start was implemented in four regions in Ethiopia: Amhara; Oromia; Southern Nations, Nationalities and Peoples (**SNNP**); and Tigray [44].

The PSI criteria for prioritising woredas to receive A360 included (personal communication, Metsehate Ayenekulu, PSI Ethiopia):

- Presence of PSI affiliated network of private health facilities or partner organisations providing SRH services
- Higher unmet need for modern contraception among married adolescent girls aged 15 to 19 years
- Larger population of adolescent girls
- Higher population density (urban setting)
- Areas with younger age at first marriage
- Limited other adolescent focused FP and RH activities

**
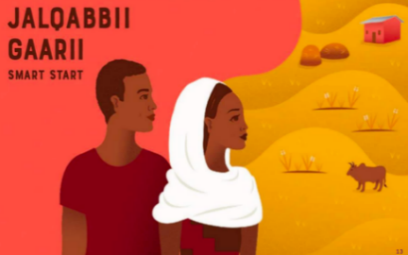
**

Figure S26 A360 logo in Ethiopia

### Research hypothesis

The primary hypothesis is that the intervention leads to an increase in mCPR over time that is greater than the increase that would have been expected to occur in the absence of the intervention.

### Study design

In Ethiopia, a before-and-after design without comparison group was used. We used population-based surveys, one survey happening in late 2017 and the other in late 2020.

#### Study unit inclusion criteria and selection

##### Region

The outcome evaluation study region and woredas were selected by PSI. Oromia Region was selected because of its relatively low mCPR as compared with other regions in Ethiopia DHS 2011 (24.9%) and its standing as having the highest unmet need for contraception (29.9%) as compared with other regions (DHS).

##### Woreda

Woredas are the equivalent to districts in Ethiopia. They are further subdivided into kebeles (or wards) or neighbourhood associations, which are the smallest geographical unit in Ethiopia. From Oromia Region, Were Jarso, Lome, Ada’a and Fentale woredas were purposively selected to be in the outcome evaluation study by the implementing agencies. Criteria used for selecting woredas for inclusion in the outcome evaluation study included:

- Good infrastructure and accessible all year round
- Close proximity to PSI head office in Addis Ababa
- No security issues
- Larger population of married adolescent girls

The characteristics of selected woredas are summarized in **Table S12**.

Table S12 Ethiopia: Characteristics of selected woredas

| **WOREDA** | **Total popn (2007)^1^** | **Popn 15-19 year old females^1^** | **Estimated no. of married girls aged 15-19^2^(sexually active)^3^** | **No. of Households^1^** | **Total no. of Kebele^1^** | **Average no. of Households per kebele** | **Average no. of married girls aged 15-19 per kebele** |
| --- | --- | --- | --- | --- | --- | --- | --- |
| East Shewa Administrative Zone | | | | | | | |
| WERE Jarso | - | - | - | - | - | - | - |
| North Shewa Administrative Zone | | | | | | | |
| Lome | 152,331 | 10,134 | 2,067 (2,005) | 35,814 | 40 | 895 | 49 |
| Ada’a | 158,572 | 8,872 | 1,810 (1,756) | 31,754 | 27 | 1,176 | 64 |
| Fentale | 104,668 | 6,083 | 1,241 (1,204) | 25,505 | 20 | 1,275 | 69 |
| Total | 497,479 | 29,530 | 6,024 (5,844) | 110,655 | 104 | 1,064 | 58 |

^1^2007 Ethiopia Census with population projections to 2017; ^2^Mini DHS 2014 (20.4% of 15-19 year old girls are married); ^3^Assume 97% of 15-19 year old married girls report having been sexually active in the past 12 months

#### Population A: 15-19 year old girls

The study population were married girls (or living as married) aged 15–19 years.

Clustered sampling of kebeles within woredas (intervention allocation units) was used. At baseline, a probability sample of 57 kebeles out of 104 kebeles was selected from across the four study woredas with probability proportional to population size. Within the selected kebele, all households were visited and a questionnaire administered to all eligible married girls aged 15 to 19 years. In households that had more than one eligible married female aged 15 to 19 years, all consenting married adolescent girls were interviewed. At endline we interviewed married females aged 15-19 years living in the 57 kebeles that were included at baseline. Although the design means that it is possible that in each site the same households and individuals may be included in the baseline and endline surveys, no attempt was made to trace individuals or households from baseline to endline.

#### Population B: Cohabitating adult

For every 15 sexually active married adolescent girls aged 15 to 19 years interviewed, one was systematically selected and asked permission to interview her husband/male partner.

### A360 activities in kebeles selected

#### According to PSI monitoring data for A360

| Figure S27 Number of girls reached per month and year in Fentale woreda, Oromia Region  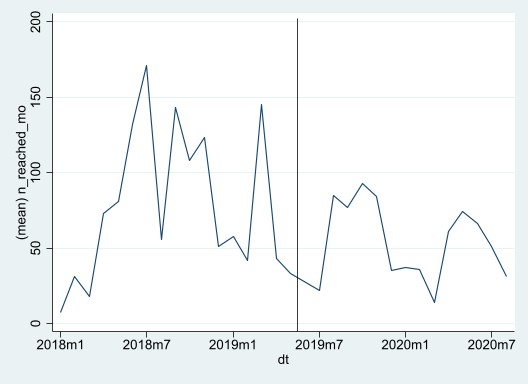 | Figure S28 Number of girls reached per month and year in Were Jarso woreda, Oromia Region  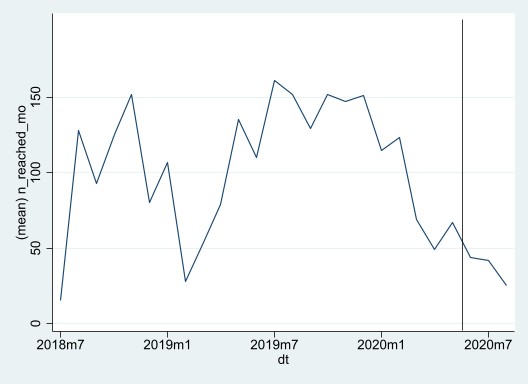 |
| --- | --- |
| Figure S29 Number of girls reached per month and year in Ada'a woreda, Oromia Region  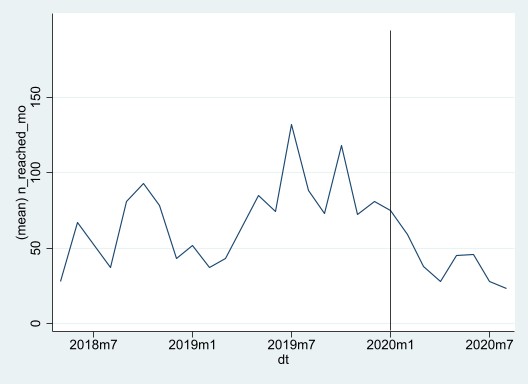 | Figure S30 Number of girls reached per month and year in Lume woreda, Oromia Region  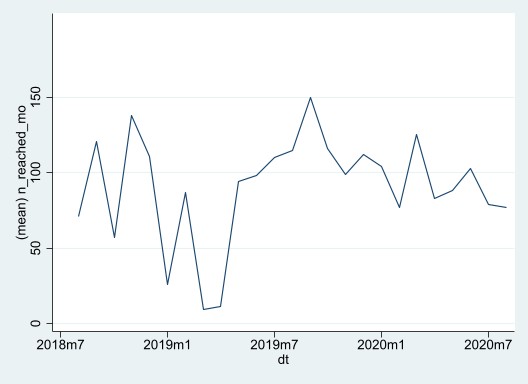 |

Note: Black line defines date at wich all kebeles were reached.

#### According to PSI team for A360

Table S13 Ethiopia: Expected exposure to the A360 intervention, according to PSI team ^1^

| **Woreda** | **A360 implementation starts in OE Sites^2^** | **Saturation or blanket coverage reached^3^** |
| --- | --- | --- |
| **Fentale** | 2018 | May 19 |
| **Wora Jarso** | 2018 | May 20 |
| **Ada’a** | 2018 | Jan 20 |
| **Lume** | 2018 | Dec 20 |

^1^ Information given by PSI team on Nov 20

^2^ Start month was not mentioned in PSI reports

^3^ SS has been rolled out to all kebeles in the woreda

Key info from PSI team: The bigger the gap between saturation and endline surveys, the lower the chance of observing an effect of A360. Accordingly, the impact should be greater in Lume and lower in Fentale.

#### According to both sources of information

Table S14 Ethiopia: Expected exposure to the A360 intervention, according to PSI monitoring data and PSI team info combined together

| **Woreda** | **A360 start date considered^1^** | **Duration of A360 activitieS^2^** |
| --- | --- | --- |
| Fentale | Apr 18 | 31 months |
| Wora Jarso | Aug 18 | 27 months |
| Ada’a | Jun 18 | 29 months |
| Lume | Aug 18 | 27 months |

^1^ Based on Figures S27-30

^2^ Endline surveys in Nov 2020

### Regression framework

#### The impact of the A360 program from baseline to endline

Two data sets will be used, one with baseline data, and the other with endline data. Datasets will be appended, and a dummy variable (e.g. time) will identify whether the survey was conducted at baseline (time ‘0’) or at endline (time ‘1’).

We will calculate mCPR at the kebele level, and then use linear regression models with mCPR (at the Kebele level) as the outcome, and Time as the predictor, as follows:

Y_i_ = β_0_ + β_1_Time + β_2_Kebele

where Y_i_ is the predicted mCPR for the i^th^ kebele; β_0_ is the intercept; β_1_ reflects the overall effect of time (endline, time ‘1’, vs baseline, time ‘0’); β_2_ is the Kebele fixed effect included to match baseline and endline kebeles, and therefore increase power.

We will adjust for the following demographic variables, which are associated with contraceptive use according to the literature: age, education level, parity, religion and wealth quintile [13]. These will be added to the model at the kebele level (e.g. average age per kebele).

**Appendix VI** (Final regression models) shows how we will present the model results.

##### Comparing to trends in modern contraceptive use observed in the literature

This will be done ‘qualitatively’ – data will be interpreted in light of trend estimates.

#### The impact of the A360 program according to level of exposure

##### The impact of the A360 program at endline among those reporting exposure to the program

Analysis will be done as described in overall study design.

Table S15 Definition of exposure to A360 interventions at endline in Ethiopia

|  | **Self-Reported Exposure** | |  |
| --- | --- | --- | --- |
|  | **Categorization** | | **Degree if 1** |
| Ethiopia (5 interventions) | 1 | 0 |  |
| Heard about smart start (no/yes) | Answers yes | Answers no | Low |
| Heard about the goal card (no/yes) | Answers yes | Answers no | Low |
| Recognizes images used in smart start activities (0, 1, or 2 images) | 1 image | 0 images | Low |
| Recognizes images used in smart start activities (0, 1, or 2 images) | 2 images | 0 or 1 image | High |
| Has seen a goal card (no/yes) | Answers yes | Answers no | High |
| Has a goal card (no/yes) | Answers yes | Answers no | High |

##### Duration of A360 activities

In this analysis, we will include an ecologic predictor, i.e. the duration of A360 activities at the Kebele level using PSI monitoring data, to estimate its contextual effect on estimates of mCPR at individual-level [43]. To do this, the 2-way interaction Time x Duration will be added to the model, where Time is 0, before the intervention, and 1, after the A360 intervention. The interaction term will be considered significant whenever the F-test yields a P-value <0.05.

##### Degree of self-reported length of time living in the community

As shown in overall study design.

### Sampling weights

Ethiopia data will be analysed accounting for sampling weights.

### Potential limitations – Ethiopia specific

#### Lack of comparison group

The lack of comparison groups in quasi-experimental designs leads to threats to internal validity and consequently, a threat to causal inference [45, 46]. The alternative explanations for observed evaluation results, include, but are not restricted to, history, instrumentation, maturation and selection bias [45-48]. These are described in detail below.

If there is an association between the different degrees of exposure to the A360 program and mCPR, even when adjusting for confounders, then this will increase the validity of our findings.

##### History/competing interventions

The before-and-after design cannot control for the presence of other events which are not part of the A360 intervention but could affect mCPR [47, 48]. In our case, these may include other sexual and reproductive health interventions occurring in our study sites (i.e. competing interventions), or the COVID-19 pandemic. We will use information from the process evaluation on the presence of other interventions that may affect mCPR. Even if no interventions have occurred, it will be important to be able to confidently state this in the outcome evaluation report. We have also added some ways to detect any COVID-19 factors that may have affected mCPR, at the endline surveys (see **Appendix V** for more details), and will describe these in our outcome evaluation report.

##### Changes in instrumentation

Due to the factors mentioned in **Appendix V**, the mode of baseline and endline surveys were slightly different. The use of masks and social distancing at endline, as well as the use of phone may have increased or decreased the rapport between interviewer and interviewee. These changes, may, therefore account for some of the observed changes in the outcome. To deal with this threat, questions kept at endline were generally the same as in baseline, and during the phone survey section, interviewers tryied to be able to see the interviewee from far, whenever possible.

##### Maturation (time)

A maturation threat to internal validity occurs when the apparent change in the outcome of interest could be due more to the intervention group changing naturally [45-47]. Specifically, mCPR could be increasing over time for reasons other than the intervention itself. To minimize this threat to validity, we will use secondary datasets on mCPR over time. Specifically, mCPR data available from other sources for the time period 2015-2020 will be examined to assess whether changes in mCPR in A360 communities between 2017 and 2020 reflect background changes in mCPR or whether mCPR appears to have increased more than would be expected during this time period.

#### Selection bias of population B

Selection bias happens when the study group is not representative of the source population [37]. Through population-based surveys we try to ensure a similar population being compared before and after the intervention. Nevertheless, we will need to verify that the composition did not change across the two periods. If the population changed in terms of any sociodemographic factors, this may need to be accounted for in the analysis.

#### Selection Bias of kebeles

As mentioned previously, selection of kebeles was proportional to size (number of households per kebele). However, the latest source of information on number of households per kebele, kebeles per woreda, etc., was obtained in 2007, at the time of the last census occurring in Ethiopia. Therefore, if there was an important change in kebele size from 2007, selection of kebeles may have been biased.

## Methodology in Tanzania

### The A360 intervention

In Tanzania, Kuwa Mjanja (‘Be Smart’) delivered life and entrepreneurial skills training alongside opt-out, youth-friendly contraceptive counselling sessions and on-site service provision. In-clinic and out-of-clinic pop up events aimed to provide a safe space for girls, with targeted messaging intended to make contraception relevant depending on their stage in life, lifestyle and priorities [49]. A30 was implemented in the following regions of Tanzania: Kagera, Geita, Mwanza, Arusha, Tabora, Tanga, Dar es Salaam, Mbeya, Iringa and Morogoro. The A360 package was delivered at the level of ward.


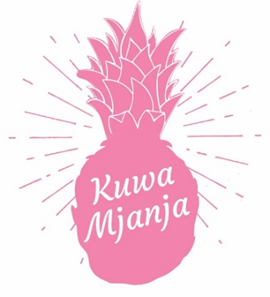


Figure S31 A360 logo in Tanzania

### Research hypothesis

The primary hypothesis is that the intervention leads to an increase in mCPR over time that is greater than the increase that would have been expected to occur in the absence of the intervention.

### Study design

As in Ethiopia, a quasi-experimental design without comparison group was used. We used before-and-after population-based surveys, one survey happening in late 2017 and the other in late 2020.

#### Study unit inclusion criteria and selection

##### Region

Mwanza Region was selected by the evaluators in collaboration with PSI because: of the high unmet need for modern contraception among girls aged 15 to 19 years relative to other A360 target regions [50]; of the absence of other large-scale family planning (FP) and sexual and reproductive health (SRH) activities; PSI has previous experience working in the region.

##### District

In consultation with PSI, the OE team has purposively selected Ilemela District in Mwanza Region as the evaluation study site.

##### Population A: 15-19 year old girls

In Tanzania, both married and unmarried girls aged 15–19 years were included in the study.

The study was restricted to urban and semi-urban wards in Ilemela District, in part because PSI focuses efforts in more densely populated areas, and in part because of resource constraints.

A simple random sample of 34 ‘streets’ (neighbourhoods) were selected from across the 15 urban and semi-urban wards of Ilemela District (**Table S16**). All households in the selected streets were visited and all eligible girls in the visited household were invited to take part in the survey. At endline we visited the same 34 streets.

Table S16 List of administrative wards within Ilemela District, Mwanza

|  | **Names of ward** | **Geographical settings** |
| --- | --- | --- |
| 1 | Buswelu | Semi urban setting |
| 2 | Nyakato | Urban setting |
| 3 | Nyamhongolo | Semi urban setting |
| 4 | Buzuruga | Urban setting |
| 5 | Mecco | Urban setting |
| 6 | Nyasaka | Semi urban setting |
| 7 | Pasiasi | Urban setting |
| 8 | Nyamanoro | Urban setting |
| 9 | Ibungilo | Urban setting |
| 10 | Kawekamo | Urban setting |
| 11 | Kirumba | Urban setting |
| 12 | Kitangiri | Urban setting |
| 13 | Ilemela | Semi urban setting |
| 14 | Kiseke | Semi urban setting |
| 15 | Kahama | Semi urban setting |
| 16 | Shibula | Rural setting |
| 17 | Bugogwa | Rural setting |
| 18 | Sangabuye | Rural setting |
| 19 | Kayenze | Rural setting |

Source: Ilemela Muncipal Council 2016. Shaded grey are urban and semi-urban wards included in the study

##### Population B: Cohabitating adult

For every 7 sexually active adolescent girls aged 15-19 years interviewed, one will be systematically selected and asked permission to interview her husband/male partner (married girls) or a co-habiting adult (unmarried girls).

### Regression framework

#### The impact of the A360 program from baseline to endline

Two data sets will be used, one with baseline data, and the other with endline data. Datasets will be appended, and a dummy variable (e.g. time) will identify whether the survey was conducted at baseline (time ‘0’) or at endline (time ‘1’).

We will calculate mCPR at the street level, and then use linear regression models with mCPR (at the street level) as the outcome, and Time as the predictor, as follows:

Y_i_ = β_0_ + β_1_Time + β_2_Street

where Y_i_ is the predicted mCPR for the i^th^ street; β_0_ is the intercept; β_1_ reflects the overall effect of time (endline, time ‘1’, vs baseline, time ‘0’); β_2_ is the street fixed effect included to match baseline and endline streets, and therefore increase power.

We will adjust for the following demographic variables, which are associated with contraceptive use according to the literature: age, education level, parity, religion and wealth quintile [13]. These will be added to the model at the street level (e.g. average age per street).

**Appendix VI** (Final regression models) shows how we will present the model results.

#### Comparing to trends in modern contraceptive use observed in the literature

This will be done ‘qualitatively’ – data will be interpreted in light of trend estimates.

#### The impact of the A360 program according to level of exposure

##### The impact of the A360 program at endline among those reporting exposure to the program

Analysis will be done as described in overall study design.

Table S17 Definition of exposure to A360 interventions at endline in Tanzania

|  |  | **Positive answer** | **Negative answer** | **Exposed** | **Not exposed** |
| --- | --- | --- | --- | --- | --- |
| Q1 | Heard about health project with pineapple as a sympol (no/yes) | Answers yes | Answers no | Answers positively to Q1 and to Q3 or Q4 | Answers positively to Q1 but not to Q3 or Q4 |
| Q2 | Heard about kuwa mjanja (no/yes) | Answers yes | Answers no | Answers positively to Q2 and to Q3 or Q4 | Answers positively to Q2 but not to Q3 or Q4 |
| Q3 | Attended a meeting, event or workshop where kuwa mjanja was mentioned or pineapple displayed (no/yes) | Answers yes | Answers no | Answers positively to Q3 and to Q1 or Q2 | Answers positively to Q3 but not Q1 or Q2 |
| Q4 | Heard sentences from the nanasi story (1 to 4 sentences) | Recognises at least one sentence | Does not recognise sentences | Answers positively to Q4 and to Q1 or Q2 | Answers positively to Q4 but not Q1 or Q2 |

This definition will have to be checked with PSI, the implementers of A360.

##### Duration of A360 activities

In this analysis, we will include an ecologic predictor, i.e. the duration of A360 activities at the street level using PSI monitoring data, to estimate its contextual effect on estimates of mCPR at individual-level [43]. To do this, the 2-way interaction Time x Duration will be added to the model, where Time is 0, before the intervention, and 1, after the A360 intervention. The interaction term will be considered significant whenever the F-test yields a P-value <0.05.

Table S18 Number of events and girls reached per month and year in Ilemela district, Tanzania

| District | Year | Month | Number of events | Number of girls reached |
| --- | --- | --- | --- | --- |
| Ilemela | 2018 | 4 | 66 | 1,078 |
| Ilemela | 2018 | 5 | 1,146 | 70,803 |
| Ilemela | 2018 | 7 | 55 | 1,659 |
| Ilemela | 2018 | 11 | 438 | 14,028 |
| Ilemela | 2019 | 1 | 10 | 281 |
| Ilemela | 2019 | 2 | 2 | 67 |
| Ilemela | 2019 | 4 | 1 | 8 |
| Ilemela | 2019 | 6 | 4 | 112 |
| Ilemela | 2019 | 9 | 18 | 796 |
| Ilemela | 2019 | 11 | 21 | 678 |
| Ilemela | 2019 | 12 | 1 | 32 |
| Ilemela | 2020 | 1 | 12 | 677 |
| Ilemela | 2020 | 2 | 14 | 784 |
| Ilemela | 2020 | 3 | 6 | 327 |

Table S19 Tanzania: Expected exposure to the A360 intervention

| **Assumptions** | |  | **Duration of A360 activities** |
| --- | --- | --- | --- |
| **A360 implementation starts in OE Sites** | **A360 fully scaled-up in OE Sites** | **Expected to reach saturation** | **Endline surveys**  **in July 2021** |
| Feb 18 | May 18 |  | x months |
| Jul 18 | Jun 19 |  | x months |

### Potential limitations – Tanzania specific

As shown in Ethiopia.

## Synthesis

Our assessment of whether A360 has achieved its desired effect will primarily be based on observing a change in mCPR between baseline and endline in Ethiopia and Nigeria and between intervention and comparison areas in Nigeria.

The following additional information will support the validity of any observed impact on mCPR

**Time trends:** Absence of important trends in mCPR in the Outcome Evaluation study geographies as estimated using other sources of data on contraceptive use

**Dose-response relationship**: Observation of a greater intervention impact among those who

report greater exposure to A360 and/or

live in a geography with a longer period of exposure to A360

**Theory of change:** Consistency in observed impact on primary and secondary A360 outcomes

**Triangulation with other data sources:** Consistency in geographical or sub-population variation in contraceptive uptake/use between

A360 OE survey data and A360 Process Evaluation data

A360 OE survey data and PSI monitoring

# Appendix I: Outcomes of interest for the A360 Outcome Evaluation – Collected at baseline and endline

Table S20 Adolescents 360 Theory of Change, outcomes of interest for the A360 Outcome Evaluation, and data collection tools

| **Outcomes** | **Definitions and Data collection tools** | |
| --- | --- | --- |
|  | **Denominator** | **Numerator** |
| Prevalence of current modern contraceptive use | Girls who are fecund (started menstruating; not pregnant; not infertile) and sexually active in the last 12 months | *Variables needed:*   - Current use of modern contraception (binary variable: not using or using) - Any known confounders (age, education, religion, parity) |
| Proportion of current modern contraceptive users who are using a LARC | Girls who are sexually active in the last 12 months and are currently using a modern contraceptive method | Currently using a long-acting (i.e. intrauterine device or implant) or permanent method (i.e. male or female sterilization); (binary variable: not using or using) |
| Modern contraceptive use in last 12 months^[[12]](#footnote-18)^ | Girls who are sexually active in the last 12 months | Used a modern method in last 12 months |
| Age at first birth | Girls who gave birth | Age at first birth |
| Age specific fertility rates | Number of girl-years of exposure 12 months before the survey | Number of births that occurred 12 months before the survey to girls aged 15-19 |
| Total unmet need for modern contraception   1. unmet need for spacing 2. unmet need for limiting | Girls who are sexually active in the last 12 months | The **total unmet need** is composed of unmet need for spacing plus the unmet need for limiting.  The numerator includes only women who were not using contraception at the time of the survey.  The nonusers were first split into   - Pregnant or postpartum amenorrhoeic (menstrual period not returned following a birth during the 2 years preceding the survey), who are then classified by whether the pregnancy or last birth (last 24 months) was:   - wanted at that time - unmet need for spacing   - or unwanted - unmet need for limiting; - Women who were neither pregnant nor postpartum amenorrhoeic will then be classified into fecund and infecund; fecund women are further split into:   - Fecund women who want children two or more years in the future, or are undecided whether/when they wanted a child - unmet need for spacing;   - Fecund women who wanted no more children - unmet need for limiting. |
| Awareness of where to obtain contraceptive services and products | Girls who are sexually active in the last 12 months and are not currently using a contraceptive method (traditional or modern) | **Awareness of where to obtain health services** was assessed through the question   - “Do you know of a place where or person from whom you would feel comfortable getting family planning services and products to delay or avoid getting pregnant?”   If the girl answers ‘No’, then she is coded 0’No’ or ‘Don’t know’ for awareness of where to obtain health services. If the girl answers ‘Yes’, then she is coded 1’Yes’ for awareness of where to obtain health services. |
| Awareness of contraceptive products | Girls who are sexually active in the last 12 months | **Awareness of contraceptive products** was assessed through the question   - “Have you ever heard of contraceptives?”   If the girl answers ‘No’, then she is coded 0’No’ or ‘Don’t know’ for awareness of contraceptive products. If the girl answers ‘Yes’, then she is coded 1’Yes’ for awareness of contraceptive products. |
| Benefits of modern contraception | Girls who heard about modern contraceptives | **Benefits** of modern contraception was assessed through the question “Using modern contraception can allow an adolescent woman girl to complete her education, find a better job and have a better life” with which the respondent must agree or disagree, scored 1 or 0, respectively. |
| Misconceptions about modern contraceptives | Girls which are sexually active in the last 12 months and heard about modern contraceptives | **Misconceptions** about contraception were assessed through three questions, with each of which the respondent must agree or disagree, scored 0 or 1, respectively. The questions include whether the woman believed that:   1. Some modern contraception can stop an adolescent woman from ever being pregnant again even after she stops using it, 2. If a modern contraception changes an adolescent woman’s menstrual bleeding, it’s bad for her health and can harm her womb, and 3. Some modern contraceptives can make adolescent women permanently fat   Sum score may therefore range between 0 and 3. With greater scores being more desirable than lower scores. |
| Agency (self-efficacy) to use modern contraceptives to prevent unintended pregnancies | Girls which are sexually active in the last 12 months and heard about modern contraceptives | **Self-efficacy** was assessed through four questions relating to the woman’s ability to access and use family planning methods, with each of which the respondent must agree or disagree, scored 1 or 0, respectively. The questions include whether she:   1. Felt able to start a conversation with her partner about contraception, 2. Felt able to use a method of contraception even if her partner did not want her to, 3. Felt able to obtain information on contraception services and products if she needed to, and 4. Felt able to obtain a contraception method if she decided to use one.   Sum score may therefore range between 0 and 4. With greater scores being more desirable than lower scores. |
| Attitudes towards the use of modern contraceptives to prevent unintended pregnancies | Girls who heard about modern contraceptives | **Attitudes** towards the use of modern contraceptives was assessed through two questions, with each of which the respondent answers approve or disapprove, scored 1 or 0:   1. Do you approve or disapprove of married couples using a modern contraceptive method to avoid or delay pregnancy? 2. Do you approve or disapprove of couples who are not married using a modern contraceptive method to avoid or delay pregnancy?   Sum score may therefore range between 0 and 2. With greater scores being more desirable than lower scores. |
| Community acceptance and social support for adolescent girls to adopt healthy sexual and reproductive health behaviours, including use of modern contraceptives | Unmarried girls which are sexually active in the last 12 months and heard about modern contraceptives | **Community acceptance** towards the use of modern contraceptives was assessed through two questions, with each of which the respondent answers approve or disapprove, scored 1 or 0, respectively:   1. Does your mother approve or disapprove of girls your age using a modern contraceptive method to avoid or delay pregnancy? 2. Does your community as a whole approve or disapprove of girls your age using a modern contraceptive method to avoid or delay pregnancy?   Sum score may therefore range between 0 and 2. With greater scores being more desirable than lower scores. |
|  | Married girls which are sexually active in the last 12 months and heard about modern contraceptives | **Community acceptance** towards the use of modern contraceptives was assessed through two questions, with each of which the respondent answers approve or disapprove, scored 1 or 0, respectively:   1. Does your husband/partner approve or disapprove of girls your age using a modern contraceptive method to avoid or delay pregnancy? 2. Does your community as a whole approve or disapprove of girls your age using a modern contraceptive method to avoid or delay pregnancy?   Sum score may therefore range between 0 and 2. With greater scores being more desirable than lower scores. |
| Not measured |  |  |
| Not measured |  |  |

LAPM, long-acting (i.e. intrauterine device or implant) or permanent method (i.e. male or female sterilization)

SRH, sexual and reproductive health

*Note: excluding the following reasons, which were only scored at endline: Favourite method was not available, Family planning services were closed/unavailable, Stopped meeting my partner/boyfriend, Stopped going outside of my house, Stopped going to the family planning services

#Note: We restricted past use to the last 12 months to decrease recall bias

# Appendix II: Other outcomes of interest for the A360 Outcome Evaluation – Only collected at endline

Table S21 Adolescents 360 Theory of Change, outcomes of interest for the A360 Outcome Evaluation related to population A, and data collection tools

| **Outcomes** | **Definitions and Data collection tools** | |
| --- | --- | --- |
|  | **Denominator** | **Numerator** |
| Future aspirations | Girls who heard about modern contraceptives | Future aspirations were assessed through four questions, with each of which the respondent indicated her agreement (strongly disagree to strongly agree):   1. I have goals for my life 2. I believe I have some tools to help me achieve my goals for my life 3. I have little control over the things that happen to me 4. I believe preventing unintended pregnancy is important to help me achieve my goals for life   Questions 1, and 2 were scored 2 if the girl says “strongly agree”, 1 if “agree” or 0 if she says “disagree” or “strongly disagree”.  Question 3 was scored 0 if the girl says “agree” or “strongly agree”, 1 if she says “disagree” and 2 if “strongly disagree”.  Question 4 was scored 4 if the girl says “strongly agree”, 3 if “agree” or 0 if she says “disagree” or “strongly disagree”.  Sum score may therefore range between 0 and 9. With greater scores being more desirable than lower scores. |
| Benefits on the use of modern contraception to prevent unintended pregnancies | Girls who heard about modern contraceptives | Benefits of contraception were assessed through the question “Using modern contraception can allow a girl to achieve her life goals” with which the respondent must agree or disagree. |
| Modern contraceptives disadvantages | Girls who heard about modern contraceptives | Modern contraceptives disadvantages were assessed through the question “What do you see as the disadvantages/negative consequences of using modern contraceptive methods?”, which then was coded as 1 if the girl responded ‘none’, and coded 0 if the girl mentioned “none” and 1 to 7 depending on the number of disadvantages mentioned. Greater scores were thus less desirable than lower scores. |
| Descriptive norms | Unmarried girls who heard about modern contraceptives | Descriptive norms towards the use of modern contraceptives was assessed through three questions, with each of which the respondent answers “Most of them”, “Less than half of them”, “None of them” or “Don’t know”:   1. How many unmarried girls aged 15-19 years in your community do you believe discuss using a method of contraception with their boyfriend/partner 2. How many unmarried girls aged 15-19 years in your community do you believe use contraceptive methods 3. How many unmarried girls aged 15-19 years in your community do you believe use contraceptive methods in secrecy from their boyfriend or family   Questions were scored 1 if the girl says “Most of them”, and 1 if she says “Less than half of them” and 0 if she says “None of them”. Sum score may therefore range between 0 and 6. With greater scores being more desirable than lower scores. |
|  | Married girls who heard about modern contraceptives | Descriptive norms towards the use of modern contraceptives was assessed through three questions, with each of which the respondent answers “Most of them”, “Less than half of them”, “None of them” or “Don’t know”:   1. How many married girls (or living as married) aged 15-19 years in your community do you believe discuss using a method of contraception with their husband/partner 2. How many married girls (or living as married) aged 15-19 years in your community do you believe use contraceptive methods 3. How many married girls (or living as married) aged 15-19 years in your community do you believe use contraceptive methods in secrecy from their husband/partner   Questions were scored 1 if the girl says “Most of them”, 1 if she says “Less than half of them” and 0 if she says “None of them”. Sum score may therefore range between 0 and 6. With greater scores being more desirable than lower scores. |

SRH, sexual and reproductive health

Table S22 Adolescents 360 Theory of Change, outcomes of interest for the A360 Outcome Evaluation related to population B, and data collection tools

| Outcomes | Definitions and data collection tools | |
| --- | --- | --- |
|  | Denominator | Numerator |
| Community acceptance and social support for adolescent girls to adopt healthy sexual and reproductive health behaviours, including use of modern contraceptives | Co-habiting adult who heard about modern contraceptives | **Attitudes** towards the use of modern contraceptives was assessed through two questions, with each of which the respondent answers approve or disapprove, scored 1 or 0, respectively:   1. Do you approve or disapprove of married couples using a modern contraceptive method to avoid or delay pregnancy? 2. Do you approve or disapprove of couples who are not married using a modern contraceptive method to avoid or delay pregnancy?   Sum score may therefore range between 0 and 2. With greater scores being more desirable than lower scores. |
| Descriptive norms* | Co-habiting adult of unmarried girl who heard about modern contraceptives | **Descriptive norms** towards the use of modern contraceptives was assessed through four questions, with each of which the respondent answers “Most of them”, “Less than half of them”, “None of them” or “Don’t know”:   1. How many husbands/partners of girls aged 15-19 years in your community do you believe discuss using a method of contraception with their wife/partner 2. How many parents/guardians of girls aged 15-19 years in your community do you believe discuss using a method of contraception with their daughter 3. How many girls aged 15-19 years in your community do you believe use contraceptive methods 4. How many girls aged 15-19 years in your community do you believe use contraceptive methods in secrecy from their husband/partner or family   Questions were scored 1 if the adult says “Most of them”, and 0 if the adult says “Less than half of them” or “None of them”. Sum score may therefore range between 0 and 4. With greater scores being more desirable than lower scores. |
|  | Husband/partner of married girl who heard about modern contraceptives | **Descriptive norms** towards the use of modern contraceptives was assessed through two questions, with each of which the respondent answers “Most of them”, “Less than half of them”, “None of them” or “Don’t know”:   1. How many husbands/partners of girls aged 15-19 years in your community do you believe discuss using a method of contraception with their wife/partner 2. How many couples in your community do you believe use contraceptive methods   Questions were scored 1 if the adult says “Most of them”, and 0 if the adult says “Less than half of them” or “None of them”. Sum score may therefore range between 0 and 2. With greater scores being more desirable than lower scores. |

SRH, sexual and reproductive health

*Only measured at endline

# Appendix III: Summary of study designs in each country / site

Table S23 Summary of study design

| **A360 country** | **Study design** | **Outcome evaluation study setting** | **Study population** | **Sampling strategy** |
| --- | --- | --- | --- | --- |
| Nigeria (South) | Cross-sectional before-and-after study with comparison group | Ogun State:   - Ado-Odo Ota LGA (I) - Shagamu LGA (C) | Unmarried girls aged 15-19 years  (12,000)  Co-habiting adults (250) | Cluster sampling design  Simple random sample of approx. 710 EAs in Ogun*  All households visited in selected EAs  All eligible girls invited to be interviewed |
| Nigeria (North) | Cross-sectional before-and-after study with comparison group | Nasarawa State:   - Doma LGA (I) - Toto LGA (C) - Karu LGA (I) - Nasarawa LGA (C) | Married girls aged 15-19 years  (4,600)  Husband/ male partner (250) | Cluster sampling design  Simple random sample of approx. 1,150 EAs in Nasarawa*  All households visited in selected EAs  All eligible girls invited to be interviewed |
| Ethiopia | Cross-sectional before-and-after study | Oromia regional state, 4 woredas purposively selected for implementation: Wara Jarso, Lome, Ada’a, Fentale woredas | Married girls aged 15-19 years (1,926)  Husband/ male partner (128) | Cluster sampling of kebele within woreda  Random sample of 57 kebele (probability proportional to population size)*  All households (HH) visited in selected kebele  All eligible girls invited to be interviewed |

LGA, Local Government Area, I, intervention, C, comparison

*Same visited at endline and baseline, where possible

# Appendix IV: Sample size calculations

## Nigeria

In Ogun State, among sexually active unmarried 15-19 year olds, we assume that between 2017 and 2020 mCPR will increase from 50% to 54% in the absence of A360 and 45% to 56% in the presence of A360. We would need to interview 1,747 sexually active unmarried girls in order to detect this increase between 2017 and 2020 in A360 exposed girls and a difference in difference of 7%.

Taking into account the sampling design, estimated non-response, and the fact that not all unmarried girls will be currently sexually active, the final target sample size is 12,048 unmarried 15-19 year old girls.

In Nasarawa State, among sexually active married 15-19 year olds, we assume that between 2017 and 2020 mCPR will increase from 13% to 14% in the absence of A360, and from 16% to 24% in the presence of A360. A sample size of 2,732 sexually active married girls would be needed to have 90% power to detect difference in differences of 7% between 2017 and 2020 in A360 exposed girls.

Taking into account the sampling design, estimated non-response, and the fact that not all married girls will be currently sexually active, the final target sample size is 4,870 married 15-19 year old girls.

Table S24 Nigeria: revised mCPR estimates

| **Setting** | **Notes** | **Original estimated study would have 90% power to detect effect size of…** | **Intervention or comparison community** | **Actual baseline mCPR (2017)** | **Estimated endline mCPR (2020)** | **Revised estimated study would have 90% power to detect effect size of…** |
| --- | --- | --- | --- | --- | --- | --- |
| Ogun |  | Difference in differences of 7% | Intervention | 44·7% | 55·7% | Difference in differences of 7·0% |
|  |  |  | Comparison | 49·8% | 53·8% |  |
| Nasarawa | Both pairs together | Difference in differences of 2% | Intervention | 16·0% | 23·8% | Difference in differences of 6·5% |
|  |  |  | Comparison | 12·9% | 14·2% |  |
|  | Pair 1: Doma/Toto |  | Intervention | 7·6% | 17·5% | Difference in differences of 8·6% |
|  |  |  | Comparison | 12·8% | 14·1% |  |
|  | Pair 2: Karu/Nasarawa |  | Intervention | 21·3% | 30·2% | Difference in differences of 7·6% |
|  |  |  | Comparison | 13·0% | 14·3% |  |

Note: (1)The endline mCPR are estimates and represent one possible scenario at endline. The study power is based on the difference in the differences in mCPR between baseline and endline and not on the actual values of mCPR at endline. There are many scenarios of endline mCPR which would give a difference in difference of e.g. 7%. (2)In Nasarawa, the original sample size calculation was not based on the two matched pair design. The table shows the study power to estimate intervention effect in each matched pair separately.

Table S25 Nigeria: estimated mCPR and sample size needed for sexually active 15-19 year olds

| **Setting** | **Group** | **mCPR 2017 (baseline)** | **mCPR 2020** | **DID in mCPR** | **Sample size (number of sexually active girls) for 90% power to detect difference** |
| --- | --- | --- | --- | --- | --- |
| Ogun | Intervention | 44·7% | 55·7% | 7·0% | 1,747 |
|  | Comparison | 49·8% | 53·8% |  |  |
| Nasarawa | Intervention | 16·0% | 23·8% |  | 2,732 |
|  | Comparison | 12·9% | 14·2% | 6·5% |  |

Table S26 Nigeria: summary of endline survey design

| **Setting** | **Sample size**  **(pop· A- women aged 15-19 years)** | **Target sample of girls aged 15-19 to be interviewed** | **Sample size**  **(pop· B)** | **Sampling area** |
| --- | --- | --- | --- | --- |
| Ogun | 1,747 | 12,048 | 250 | 716 EAs |
| Nasarawa | 2,732 | 4,870 | 250 | 621 EAs |

Table S27 Nigeria (Ogun): Details of sample size calculation

|  | **90% power to detect DID 7% increase in mCPR** |
| --- | --- |
| Target sample of sexually active 15-19 year olds (effective sample size) | 1,588 |
| Design effect* | 1·1 |
| Sample size of sexually active fecund 15-19 year old girls  (effective sample size * design effect) | 1,747  (1,843 sexually active girls) |
| Target number of 15-19 year old girls  -estimate 14·5 % will report that they have been sexually active in the past year based on A360 baseline data & are fecund | 12,048 |
| Target sample of 15-19 year olds girls  accounting for an estimated 11% non-response based on A360 baseline survey | 13,373 |

Table S28 Nigeria (Nasarawa): Details of sample size calculation

|  | **90% power to detect DID 7% increase in mCPR** |
| --- | --- |
| Target sample of sexually active 15-19 year olds (effective sample size) | 1,366 |
| Design effect* | 2 |
| Sample size of sexually active fecund 15-19 year old girls  (effective sample size * design effect) | 2,732  (4,417 sexually active girls) |
| Target number of 15-19 year old girls  -estimate 56·1 % will report that they have been sexually active in the past year based on A360 baseline data & are fecund | 4,870 |
| Target sample of 15-19 year olds girls  accounting for an estimated 3% non-response based on A360 baseline survey | 5,016 |

## Ethiopia

In the four study woredas, among sexually active married 15-19 year olds, we have assumed that between 2017 and 2020 mCPR will increase from 44% to 51% in the presence of A360 (PSI estimate). This represents an absolute increase of 7% and a relative increase of 15% between 2017 and 2020 in A360 exposed married girls. A sample size of 1,132 sexually active married girls aged 15-19 years would give us 90% power to detect this difference based on the assumptions in **Table S30**.

Taking into account the sampling design, estimated non-response, and the fact that not all married adolescent girls will be currently sexually active, the final target sample size is 1,926 married girls aged 15 to 19 years (**Tables S30 and S31**). In this scenario, we have estimated that the design effect will be 1·5. If we took a more conservative design effect of 2 we would have 80% power to detect the same effect size (**Table S31**).

Table S29 Ethiopia: Table of assumptions for key parameters required for sample size calculations

| Parameter | **Estimate** | **Source** |
| --- | --- | --- |
| Proportion of 15-19 year old females who are married (or living together) | 20·4% | Mini DHS 2014 |
| Proportion of 15-19 year old females who are unmarried (not currently married) | 79·6% | Mini DHS 2014 |
| Proportion of married 15-19 year olds who report sexual activity in the past year | 97% | DHS 2011 (all ages married) |
| Proportion of households with resident who is female aged 15-19 years | 26·7% | Estimated using 2007 census data |

Table S30 Ethiopia: Final target sample size taking into account design effect

| **Scenario** | **Best guess** | **More conservative estimate** |
| --- | --- | --- |
|  | 90% power to detect 15% increase in mCPR | 80% power to detect 15% increase in mCPR |
| Target sample of sexually active married 15-19 year olds | 1,132 | 846 |
| Target sample of all married 15-19 year old girls^1^  includes non-sexually married active girls | 1,167 | 872 |
| Total sample size of 15-19 year old girls (effective sample size)^2^  effective sample size  taking into account estimated non-response | 1,284 | 959 |
| Design effect^3^ | 1.5 | 2 |
| Sample size (effective sample size * design effect) | 1,926 | 1,918 |

^1^Estimate 97% of 15-19 year old married girls will report that they have been sexually active in the past year; ^2^Estimate 10% of girls approached will refuse to participate;

^3^Intracluster correlation coefficient=0.02 at Kebele level (PSU), 45 clusters, 28 eligible girls/cluster

Table S31 Ethiopia: sampling strategy

| **Study sites** | **Estimated no. of girls aged 15-19^1^** | **Estimated no. of married girls aged 15-19^2^** | **Estimated no. of sexually active married girls aged 15-19^3^** | **Target sample of married sexually active girls aged 15-19^4^** | **Total no. of married girls aged 15-19 to be interviewed^4^** | **Target sample of husbands/male partners** |
| --- | --- | --- | --- | --- | --- | --- |
| Girar Jarso, Lome, Ada’a and Fentale | 29,530 | 6,024 | 5,844 | 1,132 | 1,926 | 128 |

^1^2007 Ethiopia Census with population projections to 2017;

^2^Mini DHS 2014 (20·4% of 15-19 year old girls are married);

^3^From Table S29 assume 97% of married girls report having been sexually active in the past 12 months;

^4^From Table S30

## Tanzania

In Ilemela District, among sexually active 15-19 year olds, we have assumed that between 2017 and 2020 mCPR will increase from 48% to 57% in the presence of A360. This represents an absolute increase of 9% and a relative increase of 19% between 2017 and 2020 in A360 exposed girls. Based on the assumptions in **Table S32**, a sample size of 947 sexually active 15-19 year olds would give us 90% power to detect this difference.

Taking into account the sampling design, estimated non-response, and the fact that not all adolescent girls will be currently sexually active, the final target sample size is 5,106 girls (corresponding to 947 sexually active girls) aged 15 to 19 years (**Tables S32 and S33**).^[[13]](#footnote-19)^

Table S32 Tanzania: Table of assumptions

| **Parameter** | **Estimate** | **Source** |
| --- | --- | --- |
| Proportion of 15-19 year old females who are married (or living together) | 5·7% | A360 baseline survey |
| Proportion of unmarried 15-19 year old females who report sexual activity in the past year | 22·5% | A360 baseline survey |
| Proportion of married 15-19 year olds who report sexual activity in the past year | 93% | A360 baseline survey |
| Proportion of 15-19 year old females who report sexual activity in the past year | 26·5% | A360 baseline survey |
| Proportion of households with potentially eligible female aged 15-19 years | 28% | A360 baseline survey |

Table S33 Tanzania: Final target sample size taking into account various estimates of design effect

|  | **90% power to detect 19% increase in mCPR** |
| --- | --- |
| Target sample of sexually active 15-19 year olds (effective sample size) | 631 |
| Design effect* | 1·5 |
| Sample size of sexually active 15-19 year old girls  (effective sample size * design effect) | 947 |
| Target number of 15-19 year old girls  -estimate 26·5 % will report that they have been sexually active in the past year based on A360 baseline data | 3574 |
| Target sample of 15-19 year olds girls  accounting for an estimated 30% non-response based on A360 baseline survey | 5106 |

*The DE calculated at baseline was 1.06 but we have used a conservative estimate of 1·5

# Appendix V: Modifications made to the A360 outcome evaluation protocol

## Changes due to COVID-19 pandemic

The endline surveys for the A360 outcome evaluation were to be administered through in-person interview in 2020, as was done during baseline surveys in 2017. Due to the COVID-19 pandemic, we had to ensure that field implementation at endline was carried out with appropriate safeguards in place.

These measures were determined in accordance with national and international standards of best practice in research and included the following:

- - Use of face masks: Enumerators were required to use a securely fastened mask around mouth and nose;
  - Use of gloves: Enumerators were required to use gloves;
  - Social distancing: Enumerators were asked to practice social distancing rules (at least one meter) at all times; interviews were conducted in a ventilated space or outside whenever possible, and only when privacy was still possible;
  - Cleaning of equipment: Ipads and any other objects (e.g. phone) used during interviews were thoroughly cleaned before and after use;
  - Interviewees: Enumerators will ask interviewees if (1) they had any COVID-19 symptoms in the past 2 weeks, (2) to wash their hands before and after the interview (or use hand sanitizer) and (3) to practice social distancing before/during/after the interview; If the interviewee said yes to (1) or refuses (2) or (3), then the enumerator had the right to decline the interview;
  - Reduced length of interviews: all face-to-face interviews could only have a maximum length of 20 minutes.

### Data collection – Population A

At baseline, population A (i.e. girls aged 15-19 years) surveys took 40 to 60 minutes, and were collected through Computer Assisted Personal Interviewing (**CAPI**). At endline, CAPI was used for the first part of the survey, and Computer Assisted Telephone Interviewing (**CATI**) for the second part of the survey. Each section took a maximum of 20 minutes duration. Participants had the option to consent only to the first section (i.e. CAPI).

The second part of the survey occurred immediately after the first. If that was not possible, the phone interview could also be conducted at a later date. It happened as follows:

- - Phone numbers of the girls or a family member were collected during the consent procedures;
  - In the event that phones are not available in the household, at the end of the CAPI, the interviewer left a cleaned and sterilised phone near the interviewee;
  - The interviewer moved to an area a short distance (around 10 meters) from the respondent’s household and proceeds with the second part of the questionnaire;
  - In the event that phones were not available in the household, at the end of the phone survey, the interviewer returned to the household to retrieve the phone; the phone was then thoroughly cleaned and sterilised.

The phone survey will be conducted immediately after the face-to-face interview, to ensure the identity of participants (interviewer will ideally see the girl in the distance). Answers to sensitive questions will be provided in a non-disclosive categorical format (i.e. 1,2,3; or a,b,c).

Finally, we added some answer-options to understand if COVID-19 pandemic influenced the girl's access and need of modern contraceptives.

### Data collection – Population B

At baseline, population B (i.e. adults) surveys took 20 to 30 minutes, and were collected through Computer Assisted Personal Interviewing (CAPI). At endline, surveys took a maximum of 20 minutes.

## Other changes

At endline, the questionnaire included questions on the exposure to the intervention and on aspirations as linking contraception use to girls life goals was a major feature of the intervention.

Table S34 Modifications made to the A360 surveys from baseline

|  | **Baseline** | **Endline** |
| --- | --- | --- |
| Background characteristics of the respondent | + | + |
| Migration and movement history | + | + (reduced) |
| Housing and assets | + | + |
| Future aspirations | - | + |
| Marital and cohabitation status | + | + |
| Reproductive history | + | + (reduced) |
| Fertility preferences | + | + |
| Knowledge and use of contraceptive methods | + | + |
| Sexual history | + | + |
| Media exposure to family planning messages | + | + |
| Exposure to the A360 programme interventions (endline) | - | + |

+, present, -, absent

# Appendix VI: Socioeconomic index

## Data collection tools

Table S35 Data collection tools to calculate the socioeconomic index in Nigeria, Ethiopia and Tanzania

| **Variable** | **Question** | **Option 1** | **Option 2** | **Option 3** |
| --- | --- | --- | --- | --- |
| Nigeria | | | | |
| Q1 | Does your household have … an electric iron? | Yes | No |  |
| Q2 | …a fan? | Yes | No |  |
| Q3 | …a television? | Yes | No |  |
| Q4 | …a refrigerator? | Yes | No |  |
| Q5 | … a generating set? | Yes | No |  |
| Q6 | …a cable TV? | Yes | No |  |
| Q7 | …electricity? | Yes | No |  |
| Q8 | What is the main material of the walls in your household? | Cane, palm, trunks, dirt (mud) | Other |  |
| Q9 | What is the main material of the floor in your household? | Earth, sand, dung | Other |  |
| Q10 | What type of fuel does your household mainly use for cooking? | Wood | Kerosene | Other |
| Q11 | Does any member of this household have a bank account? | Yes | No |  |
| Ethiopia | | | | |
| Q1 | Does your household have… electricity? | Yes | No |  |
| Q2 | … a radio? | Yes | No |  |
| Q3 | … a television? | Yes | No |  |
| Q4 | … a refrigerator? | Yes | No |  |
| Q5 | … an electric mitad? | Yes | No |  |
| Q6 | … a table? | Yes | No |  |
| Q7 | … a chair? | Yes | No |  |
| Q8 | … a bed with cotton/sponge/spring mattress? | Yes | No |  |
| Q9 | Does any member of this household have a bank account? | Yes | No |  |
| Q10 | What is the main source of drinking water for members of your household? | Piped to yard / plot | Other |  |
| Q11 | What kind of toilet facility do members of your household usually use? | Pit latrine without slab / open pit | No facility / bush / field | Other |
| Q12 | What type of fuel does your household mainly use for cooking? | Electricity | Wood | Other |
| Q13 | What is the main material of the floor in your household? | Earth / sand | Other |  |
| Q14 | What is the main material of the exterior walls in your household? | Bamboo with mud | Other |  |
| Q15 | What is the main material of the roof in your household? | Metal / corrugated iron | Other |  |
| Tanzania | | | | |
| Q1 | Does your household have…Electricity? | Yes | No |  |
| Q2 | … A television? | Yes | No |  |
| Q3 | …A radio? | Yes | No |  |
| Q4 | …An iron? | Yes | No |  |
| Q5 | Does any member of this household have a bank account? | Yes | No |  |
| Q6 | What is the main material of the floor of your dwelling? | Earth/Sand/Dung | Cement/Concrete | Other |
| Q7 | What is the main material of the exterior walls of your dwelling? | Cement blocks | Other |  |
| Q8 | What is the main material of the roof of your dwelling? | Iron Sheet | Grass/Thatch/Palm Leaf/Mud | Other |
| Q9 | What type of fuel does your household mainly use for cooking? | Firewood | Charcoal | Other |
| Q10 | What is the main source of energy for lighting in the household? | Electricity | Battery/Solar powered Flashlight or Lamp | Other |

##

# Appendix VII: STATA commands

## Stata commands – Nigeria specific

The following commands are used to generate wealth quintiles and were obtained from the Equity Tool website [14], assuming variable names and options coded as shown in the Table above. All missing values are dropped.

### Rural respondents

1. Describe each observation using tabulate commands.
2. Recode answers to national scores, as follows:

recode Q1 (1=0.116581308794849) (2=-0.0562005155124444) (else = .), generate (Q1_NAT)

recode Q2 (1=0.0941284808856873) (2=-0.0774279792145419) (else = .), generate (Q2_NAT)

recode Q3 (1=0.0856057040104315) (2=-0.0767147788547901) (else = .), generate (Q3_NAT)

recode Q4 (1=0.144767176652353) (2=-0.0327110608564607) (else = .), generate (Q4_NAT)

recode Q5 (1=0.103097002704218) (2=-0.0357715849447168) (else = .), generate (Q5_NAT)

recode Q6 (1=0.168697222351427) (2=-0.0202911566232043) (else = .), generate (Q6_NAT)

recode Q7 (1=0.0673324312816635) (2=-0.0820788543077513) (else = .), generate (Q7_NAT)

recode Q8 (1=-0.101075555661748) (2=0.0467240402690847) (else = .), generate (Q8_NAT)

recode Q9 (1=-0.0990777198087143) (2=0.0506955377540253) (else = .), generate (Q9_NAT)

recode Q10 (1=-0.0817571630833239) (2=0.203665709317909) (3=0.0602793427055348) (else = .), generate (Q10_NAT)

recode Q11 (1=0.0990074078352956) (2=-0.054447302256893) (else = .), generate (Q11_NAT)

1. Calculate the sum of the national scores

gen NationalScore = Q1_NAT+ Q2_NAT+ Q3_NAT+ Q4_NAT+ Q5_NAT+ Q6_NAT+ Q7_NAT+ Q8_NAT+ Q9_NAT+ Q10_NAT+ Q11_NAT

1. Assign respondents to national quintiles based on their national scores

generate NationalQuintile = .

replace NationalQuintile = 1 if NationalScore > -100 & NationalScore <-0.569755

replace NationalQuintile = 2 if NationalScore >=-0.569755

replace NationalQuintile = 3 if NationalScore >=-0.41837

replace NationalQuintile = 4 if NationalScore >=0.063307

replace NationalQuintile = 5 if NationalScore >=0.578532

replace NationalQuintile = . if NationalScore ==.

1. We now have a variable 'NationalQuintile' containing the national quintile for each respondent.

tabulate NationalQuintile

### Urban respondents

1. Describe each observation using tabulate commands.
2. Recode answers to urban scores, as follows:

recode Q1 (1=0.0776157101536467) (2=-0.0995187764381914) (else = .), generate (Q1_URB)

recode Q2 (1=0.0528773078085572) (2=-0.141779429548896) (else = .), generate (Q2_URB)

recode Q3 (1=0.052825971153273) (2=-0.13272598377912) (else = .), generate (Q3_URB)

recode Q4 (1=0.111778180352775) (2=-0.0544530801699798) (else = .), generate (Q4_URB)

recode Q5 (1=0.0907740959351771) (2=-0.0522264392228614) (else = .), generate (Q5_URB)

recode Q6 (1=0.141978131457421) (2=-0.0350925815642241) (else = .), generate (Q6_URB)

recode Q7 (1=0.0311207438553126) (2=-0.1547714884616) (else = .), generate (Q7_URB)

recode Q8 (1=-0.194361473857033) (2=0.0209937007324092) (else = .), generate (Q8_URB)

recode Q9 (1=-0.176452581078634) (2=0.0239743981024329) (else = .), generate (Q9_URB)

recode Q10 (1=-0.147568013508554) (2=0.13581980733905) (3=0.0162224763142865) (else = .), generate (Q10_URB)

recode Q11 (1=0.0677125721878897) (2=-0.0885297793802381) (else = .), generate (Q11_URB)

1. Calculate the sum of the urban scores

gen UrbanScore = Q1_URB+ Q2_URB+ Q3_URB+ Q4_URB+ Q5_URB+ Q6_URB+ Q7_URB+ Q8_URB+ Q9_URB+ Q10_URB+ Q11_URB

1. Assign respondents to Urban quintiles based on their Urban scores

generate UrbanQuintile = .

replace UrbanQuintile = 1 if UrbanScore > -100 & UrbanScore < -0.519563

replace UrbanQuintile = 2 if UrbanScore >=-0.519563

replace UrbanQuintile = 3 if UrbanScore >=-0.050624

replace UrbanQuintile = 4 if UrbanScore >=0.288265

replace UrbanQuintile = 5 if UrbanScore >=0.521642

replace UrbanQuintile = . if UrbanScore ==.

1. We now have a variable 'UrbanQuintile' containing the urban quintile for each respondent.

tabulate UrbanQuintile

## Stata commands – Ethiopia specific

The following commands are used to generate wealth quintiles and were obtained from the Equity Tool website [34] assuming variable names and options coded as shown in the Table above. It also assumes that the dataset includes a variable called UrbanM4M indicating whether each respondent lives in an urban (‘1’) or rural area (‘2’). All missing values are dropped.

### Rural respondents

1. Describe each observation using tabulate commands.
2. Recode answers to national scores, as follows:

recode Q1 (1=0.302612474105336) (2=-0.0350760745004816) (else = .), generate (Q1_RUR)

recode Q2 (1=0.13040533657055) (2=-0.0361459087272734) (else = .), generate (Q2_RUR)

recode Q3 (1=0.553530657182426) (2=-0.0168856825024964) (else = .), generate (Q3_RUR)

recode Q4 (1=0.793970733951432) (2=-0.00574326042554847) (else = .), generate (Q4_RUR)

recode Q5 (1=0.843022485623207) (2=-0.00512543409181437) (else = .), generate (Q5_RUR)

recode Q6 (1=0.174469223821027) (2=-0.0568463355831167) (else = .), generate (Q6_RUR)

recode Q7 (1=0.120358518035892) (2=-0.0626800486841155) (else = .), generate (Q7_RUR)

recode Q8 (1=0.187322686624023) (2=-0.0549576074259425) (else = .), generate (Q8_RUR)

recode Q9 (1=0.194479224051596) (2=-0.0379188691164965) (else = .), generate (Q9_RUR)

recode Q10 (1=0.524441347882877) (2=-0.00811568788079159) (else = .), generate (Q10_RUR)

recode Q11 (1=0.211173219195302) (2=-0.197607159474316) (3=0.0358419422454398) (else = .), generate (Q11_RUR)

recode Q12 (1=0.888989320174884) (2=-0.0265162351180398) (3=0.18992844111447) (else = .), generate (Q12_RUR)

recode Q13 (1=-0.0616132208105327) (2=0.132723728143794) (else = .), generate (Q13_RUR)

recode Q14 (1=0.0471855919365102) (2=-0.0930447799351904) (else = .), generate (Q14_RUR)

recode Q15 (1=0.141773190945636) (2=-0.104539391862835) (else = .), generate (Q15_RUR)

1. Calculate the sum of the rural scores

gen double RuralScore = Q1_RUR+ Q2_RUR+ Q3_RUR+ Q4_RUR+ Q5_RUR+ Q6_RUR+ Q7_RUR+ Q8_RUR+ Q9_RUR+ Q10_RUR+ Q11_RUR+ Q12_RUR+ Q13_RUR+ Q14_RUR+ Q15_RUR if UrbanM4M == 2

1. Calculate national scores, based on the rural scores.

gen double NationalScore = .

replace NationalScore = -0.55242+0.459389*RuralScore if UrbanM4M ==2

1. Assign respondents to national quintiles based on their national scores

generate NationalQuintile = .

replace NationalQuintile = 1 if NationalScore > -100 & NationalScore <-0.668991697501

replace NationalQuintile = 2 if NationalScore >=-0.668991697501

replace NationalQuintile = 3 if NationalScore >=-0.533857462052

replace NationalQuintile = 4 if NationalScore >=-0.365488678751

replace NationalQuintile = 5 if NationalScore >=-0.070938341752

replace NationalQuintile = . if NationalScore ==.

1. We now have a variable 'NationalQuintile' containing the national quintile for each respondent.

tabulate NationalQuintile

### Urban respondents

1. Describe each observation using tabulate commands.
2. Recode answers to urban scores, as follows:

recode Q1 (1=0.0226501546446774) (2=-0.272875055083384) (else = .), generate (Q1_URB)

recode Q2 (1=0.0635439563898256) (2=-0.0577925325997976) (else = .), generate (Q2_URB)

recode Q3 (1=0.0721570469702142) (2=-0.145061405128842) (else = .), generate (Q3_URB)

recode Q4 (1=0.134474951289948) (2=-0.0652335202255239) (else = .), generate (Q4_URB)

recode Q5 (1=0.145603477657478) (2=-0.0658894138205688) (else = .), generate (Q5_URB)

recode Q6 (1=0.0536186538097468) (2=-0.0743616366704518) (else = .), generate (Q6_URB)

recode Q7 (1=0.0412249023185786) (2=-0.0752376726980539) (else = .), generate (Q7_URB)

recode Q8 (1=0.0488432701241907) (2=-0.143732456544811) (else = .), generate (Q8_URB)

recode Q9 (1=0.0550824989076294) (2=-0.106550498447792) (else = .), generate (Q9_URB)

recode Q10 (1=0.0624922936191401) (2=-0.0872830981423537) (else = .), generate (Q10_URB)

recode Q11 (1=-0.0598220957744794) (2=-0.210173944519007) (3=0.0453688566773257) (else = .), generate (Q11_URB)

recode Q12 (1=0.194998244994968) (2=-0.201964302804422) (3=0.000606880399777) (else = .), generate (Q12_URB)

recode Q13 (1=-0.177740659992874) (2=0.0444139346279375) (else = .), generate (Q13_URB)

recode Q14 (1=-0.047279347192829) (2=0.0558325929959501) (else = .), generate (Q14_URB)

recode Q15 (1=0.0169124260701114) (2=-0.134345374269757) (else = .), generate (Q15_URB)

1. Calculate the sum of the urban scores

gen double UrbanScore = Q1_URB+ Q2_URB+ Q3_URB+ Q4_URB+ Q5_URB+ Q6_URB+ Q7_URB+ Q8_URB+ Q9_URB+ Q10_URB+ Q11_URB+ Q12_URB+ Q13_URB+ Q14_URB+ Q15_URB if UrbanM4M == 1

1. Assign respondents to Urban quintiles based on their Urban scores

generate UrbanQuintile = .

replace UrbanQuintile = 1 if UrbanScore > -100 & UrbanScore < -0.628796283958

replace UrbanQuintile = 2 if UrbanScore >=-0.628796283958

replace UrbanQuintile = 3 if UrbanScore >=-0.181762470211

replace UrbanQuintile = 4 if UrbanScore >=0.22717610433

replace UrbanQuintile = 5 if UrbanScore >=0.633549303759

replace UrbanQuintile = . if UrbanScore ==.

1. We now have a variable 'UrbanQuintile' containing the urban quintile for each respondent.

tabulate UrbanQuintile

Alternatively to 4) and 5), we can calculate national scores based on the urban scores, as follows:

1. Calculate national scores, based on the urban scores.

gen double NationalScore = .

replace NationalScore = 1.205567+1.150764*UrbanScore if UrbanM4M ==1

1. Assign respondents to national quintiles based on their national scores

generate NationalQuintile = .

replace NationalQuintile = 1 if NationalScore > -100 & NationalScore <-0.668991697501

replace NationalQuintile = 2 if NationalScore >=-0.668991697501

replace NationalQuintile = 3 if NationalScore >=-0.533857462052

replace NationalQuintile = 4 if NationalScore >=-0.365488678751

replace NationalQuintile = 5 if NationalScore >=-0.070938341752

replace NationalQuintile = . if NationalScore ==.

1. We now have a variable 'NationalQuintile' containing the national quintile for each respondent.

tabulate NationalQuintile

## Stata commands – Tanzania specific

The following commands are used to generate wealth quintiles and were obtained from the Equity Tool website [34] assuming variable names and options coded as shown in the Table above. It also assumes that the dataset includes a variable called UrbanM4M indicating whether each respondent lives in an urban (‘1’) or rural area (‘2’). All missing values are dropped.

### Rural respondents

1. Describe each observation using tabulate commands.
2. Recode answers to national scores

recode Q1 (1=0.169659012318642) (2=-0.0488815569173983) (else = .), generate (Q1_NAT)

recode Q2 (1=0.170988070175416) (2=-0.042627992435339) (else = .), generate (Q2_NAT)

recode Q3 (1=0.0412906593265701) (2=-0.0445925448323206) (else = .), generate (Q3_NAT)

recode Q4 (1=0.12226292994139) (2=-0.0364300330562009) (else = .), generate (Q4_NAT)

recode Q5 (1=0.0717940866021087) (2=-0.0427973388816026) (else = .), generate (Q5_NAT)

recode Q6 (1=-0.13113143317996) (2=0.183287150016319) (3=0.0452558103444119) (else = .), generate (Q6_NAT)

recode Q7 (1=0.133170190832932) (2=-0.0286929194064376) (else = .), generate (Q7_NAT)

recode Q8 (1=0.0634304954843373) (2=-0.201520330471286) (3=-0.0647562433533058) (else = .), generate (Q8_NAT)

recode Q9 (1=-0.0899310549527644) (2=0.232130141045092) (3=0.0837255245897975) (else = .), generate (Q9_NAT)

recode Q10 (1=0.198526694713869) (2=-0.123647653580005) (3=-0.0194621454099437) (else = .), generate (Q10_NAT)

1. Calculate the sum of the national scores

gen double NationalScore = Q1_NAT+ Q2_NAT+ Q3_NAT+ Q4_NAT+ Q5_NAT+ Q6_NAT+ Q7_NAT+ Q8_NAT+ Q9_NAT+ Q10_NAT

1. Assign respondents to national quintiles based on their national scores

generate NationalQuintile = .

replace NationalQuintile = 1 if NationalScore > -100 & NationalScore <-0.567605575546622

replace NationalQuintile = 2 if NationalScore >=-0.567605575546622

replace NationalQuintile = 3 if NationalScore >=-0.413390997797251

replace NationalQuintile = 4 if NationalScore >=-0.0587787982076406

replace NationalQuintile = 5 if NationalScore >=0.627948811277747

replace NationalQuintile = . if NationalScore ==.

1. You should now have a variable 'NationalQuintile' containing the national quintile for each respondent.

tabulate NationalQuintile

### Urban respondents

1. Recode answers to urban scores

recode Q1 (1=0.0922201696999626) (2=-0.116842897480115) (else = .), generate (Q1_URB)

recode Q2 (1=0.0993372067851843) (2=-0.0893671368935192) (else = .), generate (Q2_URB)

recode Q3 (1=0.0390861113316249) (2=-0.0686824232504123) (else = .), generate (Q3_URB)

recode Q4 (1=0.0818380548258137) (2=-0.0628115031688395) (else = .), generate (Q4_URB)

recode Q5 (1=0.047632232016071) (2=-0.0756551833303691) (else = .), generate (Q5_URB)

recode Q6 (1=-0.238343937859736) (2=0.0738192605252541) (3=-0.012060896) (else = .), generate (Q6_URB)

recode Q7 (1=0.0677564699389386) (2=-0.0421172523984567) (else = .), generate (Q7_URB)

recode Q8 (1=0.0229294787898856) (2=-0.470780301794038) (3=-0.182391821235289) (else = .), generate (Q8_URB)

recode Q9 (1=-0.191588080869143) (2=0.0786932584205091) (3=-0.0105372312890768) (else = .), generate (Q9_URB)

recode Q10 (1=0.108010517615222) (2=-0.251617784718242) (3=-0.101626878268823) (else = .), generate (Q10_URB)

1. Calculate the sum of the urban scores

gen double UrbanScore = Q1_URB+ Q2_URB+ Q3_URB+ Q4_URB+ Q5_URB+ Q6_URB+ Q7_URB+ Q8_URB+ Q9_URB+ Q10_URB

1. Assign respondents to Urban quintiles based on their Urban scores

generate UrbanQuintile = .

replace UrbanQuintile = 1 if UrbanScore > -100 & UrbanScore < -0.528655195608735

replace UrbanQuintile = 2 if UrbanScore >=-0.528655195608735

replace UrbanQuintile = 3 if UrbanScore >=-0.0851883981376886

replace UrbanQuintile = 4 if UrbanScore >=0.373024512082338

replace UrbanQuintile = 5 if UrbanScore >=0.601449040696025

replace UrbanQuintile = . if UrbanScore ==.

1. You should now have a variable 'UrbanQuintile' containing the urban quintile for each respondent.

tabulate UrbanQuintile

# Appendix VIII: Dummy tables

Table S36 Reasons for non-response and response rates at baseline and endline for girls aged 15-19 years (n, %) [51]

|  | Baseline (n=) | | Endline (n=) | |
| --- | --- | --- | --- | --- |
|  | Comparison (n=)^1^ | Intervention (n=) | Comparison (n=) | Intervention (n=) |
| **Unmarried girls aged 15-19 identified** |  |  |  |  |
| **Interviewed for face-to-face** |  |  |  |  |
| **Reasons for non-response for face-to-face surveys** |  |  |  |  |
| Reason x |  |  |  |  |
| Reason y |  |  |  |  |
| Respondent refused |  |  |  |  |
| Respondent was <17 and cohabitating adult refused consent for interview of minor |  |  |  |  |
| **Face-to-face response rate^2^** |  |  |  |  |
| **Interviewed for face-to-face but not to phone surveys^2^** |  |  |  |  |
| **Reasons for non-response for phone surveys** |  |  |  |  |
| Reason x |  |  |  |  |
| Reason y |  |  |  |  |
| No network available |  |  |  |  |
| Respondent refused |  |  |  |  |
| Respondent was <17 and cohabitating adult refused consent for interview of minor |  |  |  |  |
| **Phone response rate^3^** |  |  |  |  |

1 Comparison and intervention will only be presented in Nigeria; analysis will be conducted separately for each matched pair

2 Denominator is identified girls

3 Denominator is interviewed girls

Table S37 Reasons for non-response and response rates at baseline and endline for co-habiting adults (n, %) [51]

|  | Baseline (n=) | | Endline (n=) | |
| --- | --- | --- | --- | --- |
|  | Comparison (n=)^1^ | Intervention (n=) | Comparison (n=) | Intervention (n=) |
| **Co-habiting adults identified** |  |  |  |  |
| **Interviewed** |  |  |  |  |
| **Reasons for non-response** |  |  |  |  |
| Reason x |  |  |  |  |
| Reason y |  |  |  |  |
| Respondent refused |  |  |  |  |
| **Response rate^2^** |  |  |  |  |

1 Comparison and intervention will only be presented in Nigeria; analysis will be conducted separately for each matched pair

2 Denominator is identified co-habiting adults

Table S38 Percentage distribution of adolescent girl respondents by age, education, religion, socioeconomic status, access to phone and marital status (%, n)

|  | Baseline (n=)^1^ |  | Endline (n=) |  |
| --- | --- | --- | --- | --- |
|  | Comparison (n=)^2^ | Intervention (n=) | Comparison (n=) | Intervention (n=) |
| Age (years) |  |  |  |  |
| 15 |  |  |  |  |
| 16 |  |  |  |  |
| 17 |  |  |  |  |
| 18 |  |  |  |  |
| 19 |  |  |  |  |
|  |  |  |  |  |
| Number of living children |  |  |  |  |
| No children |  |  |  |  |
| 1 child |  |  |  |  |
| 2 children |  |  |  |  |
| 3 or more children |  |  |  |  |
|  |  |  |  |  |
| Ever attended school |  |  |  |  |
| Yes |  |  |  |  |
| No |  |  |  |  |
|  |  |  |  |  |
| Education status |  |  |  |  |
| In school |  |  |  |  |
| out of school |  |  |  |  |
|  |  |  |  |  |
| Education level |  |  |  |  |
| NEVER ATTENDED |  |  |  |  |
| PRIMARY |  |  |  |  |
| SECONDARY/TECHNICAL/HIGHER |  |  |  |  |
| Don’t know |  |  |  |  |
| No response |  |  |  |  |
|  |  |  |  |  |
| Religion |  |  |  |  |
| Religion 1 |  |  |  |  |
| Religion 2 |  |  |  |  |
| Don’t know |  |  |  |  |
| No response |  |  |  |  |
|  |  |  |  |  |
| Wealth quintile^3^ |  |  |  |  |
| 1^st^ Quintile |  |  |  |  |
| 2^nd^ Quintile |  |  |  |  |
| 3^rd^ Quintile |  |  |  |  |
| 4^th^ Quintile |  |  |  |  |
| 5^th^ Quintile |  |  |  |  |
|  |  |  |  |  |
| Mobile phone access |  |  |  |  |
| Owns phone |  |  |  |  |
| Accesses mobile phone at least once a week |  |  |  |  |
| Accesses mobile phone less than once a week |  |  |  |  |
| No mobile phone access |  |  |  |  |
| Don’t know |  |  |  |  |
| No response |  |  |  |  |

1 In Ethiopia, data will be presented separately for eached Wareda: Wara Jarso, Lome, Ada’a and Fentale woredas

2 Comparison and intervention will only be presented in Nigeria; analysis will be conducted separately for eached matched pair. i.e. One Table will show the results for Ogun Ado-Odo Ota (Ix) vs. Shagamu (Cx), another table will show the results for Nasarawa Doma (Ix) vs. Toto (Cx) and another table will show the results for Nasarawa Karu (Ix) Nasarawa (Cx)

3 Wealth quintiles range from poorest (1st and 2nd quintiles) to richest (4th and 5th quintiles).

Table S39 Background characteristics of husbands and co-habiting adult respondents surveyed (%,n)

|  | Baseline (n=) |  | Endline (n=) |  |
| --- | --- | --- | --- | --- |
|  | Comparison (n=)^1^ | Intervention (n=) | Comparison (n=) | Intervention (n=) |
| Relationship with adolescent girl |  |  |  |  |
| Husband |  |  |  |  |
| Co-habiting partner |  |  |  |  |
| Mother |  |  |  |  |
| Father |  |  |  |  |
| Other^2^ |  |  |  |  |
| No response |  |  |  |  |
|  |  |  |  |  |
| Age (years) |  |  |  |  |
| 20-29 |  |  |  |  |
| 30-39 |  |  |  |  |
| 40-49 |  |  |  |  |
| 50-59 |  |  |  |  |
| >59 |  |  |  |  |
| Don’t know |  |  |  |  |
|  |  |  |  |  |
| Education |  |  |  |  |
| NEVER ATTENDED |  |  |  |  |
| PRIMARY |  |  |  |  |
| SECONDARY/TECHNICAL/HIGHER |  |  |  |  |
| Don’t know |  |  |  |  |
| No response |  |  |  |  |
|  |  |  |  |  |
| Religion |  |  |  |  |
| Religion 1 |  |  |  |  |
| Religion 2 |  |  |  |  |
| Don’t know |  |  |  |  |
| No response |  |  |  |  |
|  |  |  |  |  |
| Mobile phone access |  |  |  |  |
| Owns phone |  |  |  |  |
| Accesses mobile phone at least once a week |  |  |  |  |
| Accesses mobile phone less than once a week |  |  |  |  |
| No mobile phone access |  |  |  |  |
| Don’t know |  |  |  |  |
| No response |  |  |  |  |

1 Comparison and intervention will only be presented in Nigeria; analysis will be conducted separately for eached matched pair

2 Includes grandmother, aunt, sister, brother, stepmother and neighbour

Table S40 Sexuality, fertility and fertility preferences of adolescent girl respondents (Estimate, 95% Confidence Interval)

|  | Baseline (n=)^1^ |  | Endline (n=) |  |
| --- | --- | --- | --- | --- |
|  | Comparison (n=)^2^ | Intervention (n=) | Comparison (n=) | Intervention (n=) |
| Timing of last intercourse (%) |  |  |  |  |
| Within last month |  |  |  |  |
| Within last 12 months |  |  |  |  |
| More than 12 months |  |  |  |  |
| Never had sex |  |  |  |  |
| Don’t know |  |  |  |  |
| No response |  |  |  |  |
|  |  |  |  |  |
| Median (interquartile range) age at first sexual intercourse |  |  |  |  |
|  |  |  |  |  |
| Ever been pregnant (%) |  |  |  |  |
| Yes |  |  |  |  |
| No |  |  |  |  |
| Don’t know |  |  |  |  |
| No response |  |  |  |  |
|  |  |  |  |  |
| Currently pregnant (%) |  |  |  |  |
| Yes |  |  |  |  |
| No |  |  |  |  |
| Don’t know |  |  |  |  |
| No response |  |  |  |  |
|  |  |  |  |  |
| Age-specific fertility rates (per 1,000) |  |  |  |  |
| 15-19 |  |  |  |  |
|  |  |  |  |  |
| Ever given birth (%) |  |  |  |  |
| Yes |  |  |  |  |
| No |  |  |  |  |
| Don’t know |  |  |  |  |
| No response |  |  |  |  |
|  |  |  |  |  |
| Median (interquartile range) age at first birth |  |  |  |  |
|  |  |  |  |  |
| Planning status of most recent birth (%) |  |  |  |  |
| Wanted then |  |  |  |  |
| Wanted later |  |  |  |  |
| Wanted no more |  |  |  |  |
| Don’t know |  |  |  |  |
| No response |  |  |  |  |
|  |  |  |  |  |
| Unmet need for modern contraception (%) |  |  |  |  |
| No unmet need |  |  |  |  |
| Unmet need for spacing ^3^ |  |  |  |  |
| Unmet need for limiting ^4^ |  |  |  |  |
| Total unmet need |  |  |  |  |

1 In Ethiopia, data will be presented separately for eached Wareda: Wara Jarso, Lome, Ada’a and Fentale woredas

2 Comparison and intervention will only be presented in Nigeria; analysis will be conducted separately for eached matched pair. i.e. One Table will show the results for Ogun Ado-Odo Ota (Ix) vs. Shagamu (Cx), another table will show the results for Nasarawa Doma (Ix) vs. Toto (Cx) and another table will show the results for Nasarawa Karu (Ix) Nasarawa (Cx)

3 Unmet need for spacing includes pregnant women whose pregnancy was mistimed; fecund women who are non-pregnant, who are not using any modern method of contraception, and say they want to wait two or more years for their first/next birth; and postpartum amenorrheic women, who are not using any modern method of contraception, and say at the time they became pregnant they had wanted to delay pregnancy.

4 Unmet need for limiting refers to pregnant women whose pregnancy was unwanted; fecund women who are non-pregnant, who are not using any modern method of contraception, and want no more children; and postpartum amenorrheic women, who are not using any modern method of contraception, and say at the time they became pregnant they had not wanted any more children.

Table S41 Family planning knowledge, attitudes and beliefs of co-habiting adult respondents’ surveyed

|  | **Baseline (n=)^1^** |  | **Endline (n=)** |  |
| --- | --- | --- | --- | --- |
|  | **Comparison (n=)^2^** | **Intervention (n=)** | **Comparison (n=)** | **Intervention (n=)** |
| Ever heard of contraception |  |  |  |  |
| Yes |  |  |  |  |
| No |  |  |  |  |
| Don’t know |  |  |  |  |
| No response |  |  |  |  |
|  |  |  |  |  |
| Misconception about contraception index |  |  |  |  |
| Misconception about contraception index components |  |  |  |  |
| Some modern contraception can stop a girl from ever being pregnant again even after she stops using it |  |  |  |  |
| If a modern contraception changes a girl’s menstrual bleeding, it's bad for her health and can harm her womb |  |  |  |  |
| Some modern contraceptives can make adolescent girls permanently fat |  |  |  |  |
|  |  |  |  |  |
| Modern contraceptives disadvantages index | - | - |  |  |
|  |  |  |  |  |
| Benefits about contraception component |  |  |  |  |
| Using modern contraception can allow a girl to complete her education, find a better job, and have a better life |  |  |  |  |
| Using modern contraception can support a girl to achieve her life goals | - | - |  |  |
|  |  |  |  |  |
| Approved of using contraception |  |  |  |  |
| Married adolescent girls aged 15–19 years using a modern contraception method to avoid or delay pregnancy |  |  |  |  |
| Unmarried sexually active adolescent girls aged 15–19 years using a modern contraception method to avoid or delay pregnancy |  |  |  |  |
|  |  |  |  |  |
| Aware of where adolescent girls may obtain health services |  |  |  |  |
|  |  |  |  |  |
| Agency (self-efficacy) index |  |  |  |  |
| Agency (self-efficacy) index components |  |  |  |  |
| It is acceptable for an adolescent girl to start a conversation with her boyfriend/husband about contraception |  |  |  |  |
| It is acceptable for an adolescent girl to obtain information on contraception services and products if she needs to |  |  |  |  |
| It is acceptable for an adolescent girl to obtain a contraception method if she decides to use one |  |  |  |  |
| It is acceptable for an adolescent girl to start a conversation with her boyfriend/husband about contraception |  |  |  |  |
|  |  |  |  |  |
| Descriptive norms (Unmarried) INDEX | - | - |  |  |
| Descriptive norms (Unmarried) | - | - |  |  |
| How many husbands/partners of girls aged 15-19 years in your community do you believe discuss using a method of contraception with their wife/partner | - | - |  |  |
| 1. How many parents/guardians of girls aged 15-19 years in your community do you believe discuss using a method of contraception with their daughter | - | - |  |  |
| 1. How many girls aged 15-19 years in your community do you believe use contraceptive methods | - | - |  |  |
| 1. How many girls aged 15-19 years in your community do you believe use contraceptive methods in secrecy from their husband/partner or family | - | - |  |  |
|  |  |  |  |  |
| 1. Descriptive norms (married) INDEX | - | - |  |  |
| 1. Descriptive norms (married) | - | - |  |  |
| 1. How many husbands/partners of girls aged 15-19 years in your community do you believe discuss using a method of contraception with their wife/partner | - | - |  |  |
| 1. How many couples in your community do you believe use contraceptive methods | - | - |  |  |

1 In Ethiopia, data will be presented separately for eached Wareda: Wara Jarso, Lome, Ada’a and Fentale woredas

2 Comparison and intervention will only be presented in Nigeria; analysis will be conducted separately for eached matched pair. i.e. One Table will show the results for Ogun Ado-Odo Ota (Ix) vs. Shagamu (Cx), another table will show the results for Nasarawa Doma (Ix) vs. Toto (Cx) and another table will show the results for Nasarawa Karu (Ix) Nasarawa (Cx)

Table S42 Family planning knowledge, attitudes and beliefs of adolescent girl respondents (%, 95% Confidence Interval)

|  | Baseline (n=)^1^ |  | Endline (n=) |  |
| --- | --- | --- | --- | --- |
|  | Comparison (n=)^2^ | Intervention (n=) | Comparison (n=) | Intervention (n=) |
| Ever heard of contraception |  |  |  |  |
| Yes |  |  |  |  |
| No |  |  |  |  |
| Don’t know |  |  |  |  |
| No response |  |  |  |  |
|  |  |  |  |  |
| misconception about contraception index |  |  |  |  |
| misconception about contraception index components |  |  |  |  |
| Some modern contraception can stop a girl from ever being pregnant again even after she stops using it |  |  |  |  |
| If a modern contraception changes a girl’s menstrual bleeding, it's bad for her health and can harm her womb |  |  |  |  |
| Some modern contraceptives can make adolescent girls permanently fat |  |  |  |  |
|  |  |  |  |  |
| Modern contraceptives disadvantages index |  |  |  |  |
|  |  |  |  |  |
| benefits about contraception component |  |  |  |  |
| Using modern contraception can allow a girl to complete her education, find a better job, and have a better life |  |  |  |  |
| Using modern contraception can support a girl to achieve her life goals | - | - |  |  |
|  |  |  |  |  |
| Approved of using contraception |  |  |  |  |
| Married adolescent girls aged 15–19 years using a modern contraception method to avoid or delay pregnancy |  |  |  |  |
| Unmarried sexually active adolescent girls aged 15–19 years using a modern contraception method to avoid or delay pregnancy |  |  |  |  |
|  |  |  |  |  |
| Future aspirations index | - | - |  |  |
| Future aspirations | - | - |  |  |
| I have goals for my life | - | - |  |  |
| I believe I have some tools to help me achieve my goals for my life | - | - |  |  |
| I have little control over the things that happen to me | - | - |  |  |
| I believe preventing unintended pregnancy is important to help me achieve my goals for life | - | - |  |  |
|  |  |  |  |  |
| Aware of where to obtain health services |  |  |  |  |
| Received high Quality health services |  |  |  |  |
|  |  |  |  |  |
| Agency (self-efficacy) index |  |  |  |  |
| Agency (self-efficacy) index components |  |  |  |  |
| It is acceptable for an adolescent girl to start a conversation with her boyfriend/husband about contraception |  |  |  |  |
| It is acceptable for an adolescent girl to obtain information on contraception services and products if she needs to |  |  |  |  |
| It is acceptable for an adolescent girl to obtain a contraception method if she decides to use one |  |  |  |  |
| It is acceptable for an adolescent girl to start a conversation with her boyfriend/husband about contraception |  |  |  |  |
|  |  |  |  |  |
| Descriptive norms INDEX | - | - |  |  |
| Descriptive norms | - | - |  |  |
| How many (un)married girls aged 15-19 years in your community do you believe discuss using a method of contraception with their partner | - | - |  |  |
| 1. How many (un)married girls aged 15-19 years in your community do you believe use contraceptive methods | - | - |  |  |
| 1. How many (un)married girls aged 15-19 years in your community do you believe use contraceptive methods in secrecy from their partner or family | - | - |  |  |

1 In Ethiopia, data will be presented separately for eached Wareda: Wara Jarso, Lome, Ada’a and Fentale woredas

2 Comparison and intervention will only be presented in Nigeria; analysis will be conducted separately for eached matched pair. i.e. One Table will show the results for Ogun Ado-Odo Ota (Ix) vs. Shagamu (Cx), another table will show the results for Nasarawa Doma (Ix) vs. Toto (Cx) and another table will show the results for Nasarawa Karu (Ix) Nasarawa (Cx)

Table S43 Degree of self-reported exposure to A360 (percentages) at endline, by age, education, religion, socioeconomic status and access to phone (%,n) [52].

|  | Degree of Self-Reported Exposure (percentages)^1^ | | |
| --- | --- | --- | --- |
|  | **None (no exposure)** | **Low (heard about A360)** | **High (participated in A360)** |
| Age (years) |  |  |  |
| 15 |  |  |  |
| 16 |  |  |  |
| 17 |  |  |  |
| 18 |  |  |  |
| 19 |  |  |  |
|  |  |  |  |
| Number of living children |  |  |  |
| No children |  |  |  |
| 1 child |  |  |  |
| 2 children |  |  |  |
| 3 or more children |  |  |  |
|  |  |  |  |
| Ever attended school |  |  |  |
| Yes |  |  |  |
| No |  |  |  |
|  |  |  |  |
| Education status |  |  |  |
| In school |  |  |  |
| out of school |  |  |  |
|  |  |  |  |
| Education level |  |  |  |
| NEVER ATTENDED |  |  |  |
| PRIMARY |  |  |  |
| SECONDARY/TECHNICAL/HIGHER |  |  |  |
| Don’t know |  |  |  |
| No response |  |  |  |
|  |  |  |  |
| Religion |  |  |  |
| Religion 1 |  |  |  |
| Religion 2 |  |  |  |
| Don’t know |  |  |  |
| No response |  |  |  |
|  |  |  |  |
| Wealth Quintile^2^ |  |  |  |
| 1^st^ Quintile |  |  |  |
| 2^nd^ Quintile |  |  |  |
| 3^rd^ Quintile |  |  |  |
| 4^th^ Quintile |  |  |  |
| 5^th^ Quintile |  |  |  |
|  |  |  |  |
| Mobile phone access |  |  |  |
| Owns phone |  |  |  |
| Accesses mobile phone at least once a week |  |  |  |
| Accesses mobile phone less than once a week |  |  |  |
| No mobile phone access |  |  |  |
| Don’t know |  |  |  |
| No response |  |  |  |

1 In Nigeria, one Table will show the results for Ogun Ado-Odo Ota (Ix) vs. Shagamu (Cx), another table will show the results for Nasarawa Doma (Ix) vs. Toto (Cx) and another table will show the results for Nasarawa Karu (Ix) Nasarawa (Cx); In Ethiopia, data will be presented separately for eached Wareda: Wara Jarso, Lome, Ada’a and Fentale woredas

2 Wealth quintiles range from poorest (1st and 2nd quintiles) to richest (4th and 5th quintiles).

Table S44 Current use of modern contraception by age, education, religion, socioeconomic status and access to phone (%,n)

|  | Baseline (n=)^1^ |  | Endline (n=) |  |
| --- | --- | --- | --- | --- |
|  | Comparison (n=)^2^ | Intervention (n=) | Comparison (n=) | Intervention (n=) |
| Age (years) |  |  |  |  |
| 15 |  |  |  |  |
| 16 |  |  |  |  |
| 17 |  |  |  |  |
| 18 |  |  |  |  |
| 19 |  |  |  |  |
|  |  |  |  |  |
| Number of living children |  |  |  |  |
| No children |  |  |  |  |
| 1 child |  |  |  |  |
| 2 children |  |  |  |  |
| 3 or more children |  |  |  |  |
|  |  |  |  |  |
| Ever attended school |  |  |  |  |
| Yes |  |  |  |  |
| No |  |  |  |  |
|  |  |  |  |  |
| Education status |  |  |  |  |
| In school |  |  |  |  |
| out of school |  |  |  |  |
|  |  |  |  |  |
| Education level |  |  |  |  |
| NEVER ATTENDED |  |  |  |  |
| PRIMARY |  |  |  |  |
| SECONDARY/TECHNICAL/HIGHER |  |  |  |  |
| Don’t know |  |  |  |  |
| No response |  |  |  |  |
|  |  |  |  |  |
| Religion |  |  |  |  |
| Religion 1 |  |  |  |  |
| Religion 2 |  |  |  |  |
| Don’t know |  |  |  |  |
| No response |  |  |  |  |
|  |  |  |  |  |
| Wealth Quintile^3^ |  |  |  |  |
| 1^st^ Quintile |  |  |  |  |
| 2^nd^ Quintile |  |  |  |  |
| 3^rd^ Quintile |  |  |  |  |
| 4^th^ Quintile |  |  |  |  |
| 5^th^ Quintile |  |  |  |  |
|  |  |  |  |  |
| Mobile phone access |  |  |  |  |
| Owns phone |  |  |  |  |
| Accesses mobile phone at least once a week |  |  |  |  |
| Accesses mobile phone less than once a week |  |  |  |  |
| No mobile phone access |  |  |  |  |
| Don’t know |  |  |  |  |
| No response |  |  |  |  |

1 In Ethiopia, data will be presented separately for eached Wareda: Wara Jarso, Lome, Ada’a and Fentale woredas

2 Comparison and intervention will only be presented in Nigeria; analysis will be conducted separately for eached matched pair. i.e. One Table will show the results for Ogun Ado-Odo Ota (Ix) vs. Shagamu (Cx), another table will show the results for Nasarawa Doma (Ix) vs. Toto (Cx) and another table will show the results for Nasarawa Karu (Ix) Nasarawa (Cx)

3 Wealth quintiles range from poorest (1st and 2nd quintiles) to richest (4th and 5th quintiles).

Table S45 Percentage distribution of married and sexually active unmarried adolescent girls aged 15–19 years who currently use contraception, by method used (DHS definition) (%, 95% Confidence Interval)

|  | **Baseline (n=)^1^** |  | **Endline (n=)** |  |
| --- | --- | --- | --- | --- |
|  | **Comparison (n=)^2^** | **Intervention (n=)** | **Comparison (n=)** | **Intervention (n=)** |
| No. of girls^3^ |  |  |  |  |
|  |  |  |  |  |
| Any method (face-to-face) |  |  |  |  |
|  |  |  |  |  |
| Any modern method^4^ |  |  |  |  |
|  |  |  |  |  |
| Modern method |  |  |  |  |
| Implant |  |  |  |  |
| Intra-uterine device |  |  |  |  |
| Injectables |  |  |  |  |
| Daily pills |  |  |  |  |
| Emergency pills |  |  |  |  |
| Male condom |  |  |  |  |
| Other modern method |  |  |  |  |
|  |  |  |  |  |
| long-acting or permanent method |  |  |  |  |
|  |  |  |  |  |
| Any traditional method |  |  |  |  |
|  |  |  |  |  |
| Not currently using |  |  |  |  |
|  |  |  |  |  |
| Don’t know |  |  |  |  |

1 In Ethiopia, data will be presented separately for eached Wareda: Wara Jarso, Lome, Ada’a and Fentale woredas

2 Comparison and intervention will only be presented in Nigeria; analysis will be conducted separately for eached matched pair. i.e. One Table will show the results for Ogun Ado-Odo Ota (Ix) vs. Shagamu (Cx), another table will show the results for Nasarawa Doma (Ix) vs. Toto (Cx) and another table will show the results for Nasarawa Karu (Ix) Nasarawa (Cx)

3 Married/unmarried girls who report sexually activity in last 12 months and are fecund

4 Modern methods include female sterilisation, male sterilisation, contraceptive pill (oral contraceptives), IUD, injectables (Depo-Provera), implants (Norplant), female condom, male condom, diaphragm, contraceptive foam and contraceptive jelly, LAM, SDM, cycle beads

Table S46 Degree of self-reported exposure to A360 (percentages) at endline, by outcome variables [52].

|  | Degree of Self-Reported Exposure (percentages)^1^ | | |
| --- | --- | --- | --- |
|  | **None (no exposure)** | **Low (heard about A360)** | **High (participated in A360)** |
| No. of girls^2^ |  |  |  |
|  |  |  |  |
| Any method (face-to-face) |  |  |  |
| Any method (phone) | - | - |  |
|  |  |  |  |
| Any modern method^3^ |  |  |  |
|  |  |  |  |
| Modern method |  |  |  |
| Implant |  |  |  |
| Intra-uterine device |  |  |  |
| Injectables |  |  |  |
| Daily pills |  |  |  |
| Emergency pills |  |  |  |
| Male condom |  |  |  |
| Other modern method |  |  |  |
|  |  |  |  |
| long-acting or permanent method |  |  |  |
|  |  |  |  |
| Any traditional method |  |  |  |
|  |  |  |  |
| Not currently using |  |  |  |
|  |  |  |  |
| Don’t know |  |  |  |

1 In Nigeria, one Table will show the results for Ogun Ado-Odo Ota (Ix) vs. Shagamu (Cx), another table will show the results for Nasarawa Doma (Ix) vs. Toto (Cx) and another table will show the results for Nasarawa Karu (Ix) Nasarawa (Cx); In Ethiopia, data will be presented separately for eached Wareda: Wara Jarso, Lome, Ada’a and Fentale woredas

Table S47 Percentage distribution of married and sexually active unmarried adolescent girls aged 15–19 years who ever used contraception, by method used (DHS definition) (%, 95% Confidence Interval)

|  | Baseline (n=)^1^ |  | Endline (n=) |  |
| --- | --- | --- | --- | --- |
|  | Comparison (n=)^2^ | Intervention (n=) | Comparison (n=) | Intervention (n=) |
| No. of girls^3^ |  |  |  |  |
|  |  |  |  |  |
| Ever use of Any method (face-to-face) |  |  |  |  |
| < 12 months |  |  |  |  |
| 12-24 months |  |  |  |  |
| > 24 months |  |  |  |  |
| EVER USE OF Any method (phone) | - | - |  |  |
|  |  |  |  |  |
| Any modern method in last 12 months^4^ |  |  |  |  |
|  |  |  |  |  |
| Modern method in last 12 months |  |  |  |  |
| Implant |  |  |  |  |
| Intra-uterine device |  |  |  |  |
| Injectables |  |  |  |  |
| Daily pills |  |  |  |  |
| Emergency pills |  |  |  |  |
| Male condom |  |  |  |  |
| Other modern method |  |  |  |  |
|  |  |  |  |  |
| long-acting or permanent method in last 12 months |  |  |  |  |
|  |  |  |  |  |
| Any traditional method in last 12 months |  |  |  |  |
|  |  |  |  |  |
| Did not use in last 12 months |  |  |  |  |
|  |  |  |  |  |
| Don’t know |  |  |  |  |

1 In Ethiopia, data will be presented separately for eached Wareda: Wara Jarso, Lome, Ada’a and Fentale woredas

2 Comparison and intervention will only be presented in Nigeria; analysis will be conducted separately for eached matched pair. i.e. One Table will show the results for Ogun Ado-Odo Ota (Ix) vs. Shagamu (Cx), another table will show the results for Nasarawa Doma (Ix) vs. Toto (Cx) and another table will show the results for Nasarawa Karu (Ix) Nasarawa (Cx)

3 Married/unmarried girls who report sexually activity in last 12 months

4 Modern methods include female sterilisation, male sterilisation, contraceptive pill (oral contraceptives), IUD, injectables (Depo-Provera), implants (Norplant), female condom, male condom, diaphragm, contraceptive foam and contraceptive jelly, LAM, SDM, cycle beads

Table S48 Difference-in-difference estimates of A360 impact on mCPR, intervention and comparison sites (Nigeria only) [15].

|  | Current use of modern contraception |
| --- | --- |
|  | Model 1 |
| MODEL variables |  |
| A360 (Ref: comparison sites) | Point estimate (95%CI) |
| Time (ref: baseline) |  |
| A360*Time |  |
|  |  |
| Demographic Variables |  |
| Age 17 and older (ref: ≤16) |  |
| Education level (Ref: XXX) |  |
| One or more children (ref: no children) |  |
| Religion (REF: XXX) |  |
| Wealth quintile (linear) |  |
|  |  |
| Constant |  |
|  |  |
| Observations |  |

* = p < 0.1; ** = p < 0.05; *** = p < 0.001.

Table S49 The estimates of A360 impact on mCPR (Ethiopia).

|  | Current use of modern contraception |
| --- | --- |
|  | Model 1 |
| Time |  |
| 0 (Baseline) | Point estimate (95%CI) |
| 1 (Endline) |  |
|  |  |
| KEBELE Fixed effect^1^ |  |
|  |  |
| Demographic Variables |  |
| Age 17 and older (ref: ≤16) |  |
| Education level (Ref: XXX) |  |
| One or more children (ref: no children) |  |
| Religion (REF: XXX) |  |
| Wealth quintile (linear) |  |
|  |  |
| Constant |  |
|  |  |
| Observations |  |

^1^Only in Ethiopia Woreda will be added as a fixed effect. In Nigeria, LGA is the same as A360 (comparison vs intervention LGA).

* = p < 0.1; ** = p < 0.05; *** = p < 0.001

Table S50 Degree of self-reported exposure to A360 by sexual and reproductive health outcomes [52].

|  | Current use of modern contraception |
| --- | --- |
|  | Model 1 |
| Degree of Self-Reported Exposure (percentages) |  |
| None (no exposure) | Point estimate (95%CI) |
| Low (Heard about A360) |  |
| High (Participated in A360) |  |
|  |  |
| Cluster Fixed effect |  |
|  |  |
| Demographic Variables |  |
| Age 17 and older (ref: ≤16) |  |
| Education level (Ref: XXX) |  |
| One or more children (ref: no children) |  |
| Religion (REF: XXX) |  |
| Wealth quintile (linear) |  |
|  |  |
| Constant |  |
|  |  |
| Observations |  |

* = p < 0.1; ** = p < 0.05; *** = p < 0.001.

# Appendix IX: Stata commands

## Declare survey design for dataset

We will use *svyset* command to declare survey design for datasets. These are specific to country, and are described in **Table S51**. The command *svydescribe* may be used after declaring survey design to describe survey data.

Table S51 Declare survey design for datasets for each country.

| **Country** | **Svyset** |
| --- | --- |
| Nigeria | svyset EA |
| Ethiopia | svyset Kebele [pw=WEIGHT], strata(Woreda) |

## Descriptive tables and summaries

For categorical variables, we will describe the data as follows

svy: tabulate *varname1 varname2*, percent column obs ci

where options *percent* display percentages; *column* percentages are presented within-column; *obs* requests that the number of observations for each cell be displayed; *ci* requests confidence intervals

For continuous variables, we will describe the data as follows

summarize *varname1*, detail

or

svy: mean *varname1*

If we are interested in describing the data by levels a categorical variable, we may use

bysort *cluster*: summarize *varname1*, detail

*svy: mean varname1, over(cluster)*

svy, subpop(if *cluster*==*level1*): mean *varname1*

## Regression (fixed effects only)

For binary outcomes, we will analyse the effects of a predictor, adjusting for confounders, as follows

svy: glm *outcome1 predictor1 confounder1 confounder2*, fam(bin) link(log) base

Or, if we are interested in risk ratios rather than coefficients, we will instead use

svy: glm *outcome1 predictor1 confounder1 confounder2*, fam(bin) link(log) base eform

For continuous outcomes, we will analyse the effects of a predictor, adjusting for confounders, as follows

svy: regress *outcome1 predictor1 confounder1 confounder2*, base

Interactions will be added to any of the models above, as needed, as *variable1*##*variable2* for 2-way interactions.

## Regression accounting for clustering

Our data is multilevel, as shown in **Figure 20**. Girl is at the lowest level of the hierarchy (level1). There are one or more girls within households (level2) and several household per primary sampling unit (**PSU**), which is EA in Nigeria or Kebele in Ethiopia (level3). Finally, the highest level of the hierarchy (level4) corresponds to LGA in Nigeria, and Wareda in Ethiopia. Note that there is no need to specify clustering at LGA level when analysing the effect in Nigeria, as LGA is already specified in the model through A360 variable (comparison vs intervention site); i.e. LGA is the same as A360. Nevertheless, in Ethiopia, a fixed effect for Woreda should be added to the model.

### Robust standard errors

For binary/binomial outcomes, we will analyse the effects of a predictor, adjusting for confounders and for clustering of observations at the EA (Nigeria) / Kebele (Ethiopia) level, generalized linear models with a log canonical link with robust standard errors as follows

For binary outcomes, we will analyse the effects of a predictor, adjusting for confounders, as follows

svy: poisson *outcome1 predictor1 confounder1 confounder2*, robust cluster(level 3)

Or, if we are interested in risk ratios rather than coefficients, we will instead use

svy: poisson *outcome1 predictor1 confounder1 confounder2*, irr robust cluster(level 3)

For continuous outcomes with normal (gaussian) distribution, we will analyse the effects of a predictor, adjusting for confounders and for clustering of observations at the highest level, using linear models with robust standard errors as follows

svy: regress *outcome1 predictor1 confounder1 confounder2*, cluster(*level3*)

### Random effects model

For binary/binomial outcomes, we will analyse the effects of a predictor, adjusting for confounders and for clustering of observations, using generalized linear models with a log canonical link as follows

svy: meglm *outcome1 predictor1 confounder1 confounder2* || level3: || level2: || level1:, family(binomial) link(log) eform

For continuous outcomes with normal (gaussian) distribution, we will analyse the effects of a predictor, adjusting for confounders and for clustering of observations, using generalized linear models with an identity canonical link as follows

svy: meglm *outcome1 predictor1 confounder1 confounder2* || level3: || level2: || level1:, family(gaussian) link(identity)

We may use command *estat group* to summarize the composition of the nested groups, *estat icc* to calculate residual intraclass correlation and *estat sd* to display variance components as standard deviations and correlations, after using the *meglm* command

# References

1. Malakoff, S., M. Cutherell, and A. Coppola, *Aligning Contraception with Family and Life Goals of Married Adolescent Girls in Northern Nigeria: The Case of Matasa Matan Arewa*. 2021, Population Services International: Washington, DC.

2. Cole, C., M. Cutherell, and M. Phillips, *Connecting Contraception to Girls’ Lives and Aspirations in Southern Nigeria: The Case of 9ja Girls*. 2020, Population Services International: Washington, DC.

3. Cutherell, M. and C. Cole, *Supporting Scalable, Youth-Powered Programming at the Community Level in Ethiopia: The Case of Smart Start*. 2019, Population Services International: Washington, DC.

4. Itad. *A360 Process Evaluation – Final Report*. 2022; Available from: <https://www.itad.com/knowledge-product/a360-process-evaluation-final-report/>.

5. MSI. *Marie Stopes International Reproductive Choices*. Marie Stopes Nigeria 2020 13/05/2021]; Available from: <https://www.msichoices.org/where-we-work/nigeria/>.

6. PPFN. *Planned Parenthood Federation of Nigeria*. 2020 13-05-2021]; Available from: <https://www.ppfn.org/>.

7. WRA. *The White Ribbon Alliance*. 2021 13/05/2021]; Available from: <https://www.whiteribbonalliance.org/nigeria/>.

8. Jackson, E., L. McDougal, and A. Raj. *Community Experiences with a School-Based Intervention to Delay Marriage among Girls in Oromia Region, Ethiopia*. 2017; Available from: <https://gehweb.ucsd.edu/wp-content/uploads/2019/04/raj_mcdougapackard-pb-oda_ethiopia_9_12_17.pdf>.

9. Kaba, M. and Z. Adugna, *SCHOOL LEVEL MENSTRUAL HYGIENE MANAGEMENT IN OROMIA REGION OF ETHIOPIA.* Ethiopian Journal of Reproductive Health, 2020. **12**(4): p. 7-7.

10. Kaba, M. and Z.A. Geletu, *Mensural Hygiene Management in Schools of Oromia Region, Ethiopia.* 2020.

11. Zou, G., *A modified poisson regression approach to prospective studies with binary data.* American journal of epidemiology, 2004. **159**(7): p. 702-706.

12. Villa, J.M., *diff: Simplifying the estimation of difference-in-differences treatment effects.* The Stata Journal, 2016. **16**(1): p. 52-71.

13. Greenland, S., R. Daniel, and N. Pearce, *Outcome modelling strategies in epidemiology: traditional methods and basic alternatives.* International journal of epidemiology, 2016. **45**(2): p. 565-575.

14. Tool, E. *Nigeria Equity Tool*. 2015 November 2020]; Available from: <https://www.equitytool.org/nigeria>.

15. Edmeades, J., H. Lantos, and F. Mekuria, *Worth the effort? Combining sexual and reproductive health and economic empowerment programming for married adolescent girls in Amhara, Ethiopia.* Vulnerable Children and Youth Studies, 2016. **11**(4): p. 339-351.

16. Meribole, E.C., et al., *The Nigerian health information system policy review of 2014: the need, content, expectations and progress.* Health Information & Libraries Journal, 2018. **35**(4): p. 285-297.

17. Olugbade, O., et al., *Family Planning Indicators Assessment and Data Quality Audit in Selected Health Facilities across Nigeria*. 2019.

18. Team, D.D. *DHIS User Manual*. 2016 [cited 2020; Available from: <https://docs.dhis2.org/2.23/en/user/html/dhis2_user_manual_en_full.html#d0e551>.

19. Ouedraogo, M., et al., *A quality assessment of Health Management Information System (HMIS) data for maternal and child health in Jimma Zone, Ethiopia.* PloS one, 2019. **14**(3): p. e0213600.

20. ASANGANSI, I., et al., *Improving the routine HMIS in Nigeria through mobile technology for community data collection.* Journal of Health Informatics in Developing Countries, 2013. **7**(1).

21. Makinde, O.A., et al., *Assessment of the routine health management information system in Imo State, Federal Republic of Nigeria.* Bethesda, Maryland: Health Systems, 2012. **20**: p. 20.

22. Belay, H., T. Azim, and H. Kassahun, *Assessment of health management information system (HMIS) performance in SNNPR, Ethiopia.* Measure Evaluation, 2013.

23. Woldegiorgis, M.A., J. Bhowmik, and W. Mekonnen, *Trends in reproductive health indicators in Ethiopia: 2000–2014.* Int J Healthcare, 2017. **3**(1): p. 10.5430.

24. Atchison, C.J., et al., *Evaluating the impact of an intervention to increase uptake of modern contraceptives among adolescent girls (15–19 years) in Nigeria, Ethiopia and Tanzania: the Adolescents 360 quasi-experimental study protocol.* BMJ open, 2018. **8**(5): p. e021834.

25. Horton, R. and H.B. Peterson, *The rebirth of family planning.* The Lancet, 2012. **380**(9837): p. 77.

26. Murro, R., et al., *Adding It Up: Investing in the Sexual and Reproductive Health of Adolescents in India.* 2021.

27. Bureau, U.C. *Subnational Population by Sex, Age, and Geographic Area*. 2021 01/10/2021]; Available from: Online Databases available from: <https://www.census.gov/geographies/mapping-files/time-series/demo/international-programs/subnationalpopulation.html>.

28. Atchison, C.J., et al., *Evaluating the impact of an intervention to increase uptake of modern contraceptives among adolescent girls (15-19 years) in Nigeria, Ethiopia and Tanzania: the Adolescents 360 quasi-experimental study protocol.* BMJ Open, 2018. **8**(5): p. e021834.

29. DHS, *Tanzania Demographic and Health Survey*. 2010.

30. DHS, *Ethiopia Demographic and Health Survey*. 2011.

31. DHS, *Nigeria Demographic and Health Survey*. 2018.

32. Armor, D.J., *Theta Reliability and Factor Scaling.* Sociological Methodology, 1973. **5**: p. 17-50.

33. Boateng, G.O., et al., *Best practices for developing and validating scales for health, social, and behavioral research: a primer.* Frontiers in public health, 2018. **6**: p. 149.

34. Tool, E. *Ethiopia Equity Tool*. 2018 November 2020]; Available from: <https://www.equitytool.org/ethiopia>.

35. Tool, E. *Tanzania Equity Tool*. 2017 November 2020]; Available from: <https://www.equitytool.org/tanzania>.

36. Erulkar, A.S., et al., *Behavior change evaluation of a culturally consistent reproductive health program for young Kenyans.* International family planning perspectives, 2004: p. 58-67.

37. Dohoo, I., S. Martin, and H. Stryhn, *Veterinary epidemiologic research. VER.* Inc., Charlottetown, PE, Canada, 2009.

38. A360. *Country Solutions - Southern Nigeria*. 2020 [cited November 2020; Available from: <http://dev-psi-a360.pantheonsite.io/countries/southern-nigeria/>.

39. A360. *Country Solutions - Northern Nigeria*. 2020 November 2020]; Available from: <http://dev-psi-a360.pantheonsite.io/countries/northern-nigeria/>.

40. White, H. and D.A. Raitzer, *Impact evaluation of development interventions: A practical guide*. 2017: Asian Development Bank.

41. Zocchetti, C., D. Consonni, and P.A. Bertazzi, *Relationship between prevalence rate ratios and odds ratios in cross-sectional studies.* International journal of epidemiology, 1997. **26**(1): p. 220-223.

42. McNutt, L.-A., et al., *Estimating the relative risk in cohort studies and clinical trials of common outcomes.* American journal of epidemiology, 2003. **157**(10): p. 940-943.

43. Morgenstern, H., *Ecologic studies*, in *Modern epidemiology*, K.J. Rothman, S. Greenland, and T.L. Lash, Editors. 2008, Lippincott Williams & Wilkins.

44. A360. *Country Solutions - Ethiopia*. 2020 November 2020]; Available from: <http://dev-psi-a360.pantheonsite.io/countries/ethiopia/>.

45. Shadish, W.R., T.D. Cook, and D.T. Campbell, *Quasi-experimental designs that either lack a control group or lack pretest observations on the outcome*, in *Experimental and quasi-experimental designs for generalized causal inference*, W.R. Shadish and T.D. Cook, Editors. 2002, Houghton Mifflin Company: Boston, MA. p. 103-134.

46. Marsden, E. and C.J. Torgerson, *Single group, pre-and post-test research designs: Some methodological concerns.* Oxford Review of Education, 2012. **38**(5): p. 583-616.

47. Robson, L., et al., *Before-and-after design: A simple evaluation design*, in *Guide to Evaluating the Effectiveness of Strategies for Preventing Work Injuries: How to Show Whether a Safety Intervention Really Works*. 2001, Centers for Disease Control and Prevention: Druid Hills.

48. Penfold, R.B. and F. Zhang, *Use of interrupted time series analysis in evaluating health care quality improvements.* Academic pediatrics, 2013. **13**(6): p. S38-S44.

49. A360. *Country Solutions - Tanzania*. 2020 November 2020]; Available from: <http://dev-psi-a360.pantheonsite.io/countries/tanzania/>.

50. Chandra-Mouli, V. and E. Akwara, *Improving access to and use of contraception by adolescents: What progress has been made, what lessons have been learned, and what are the implications for action?* Best Practice & Research Clinical Obstetrics & Gynaecology, 2020.

51. Acharya, R., et al., *Broadening girls' horizons: Effects of life skills education programme in rural Uttar Pradesh.* 2009.

52. Williams, T., et al., *Evaluation of the Africa Youth Alliance Program in Ghana, Tanzania and Uganda: Impact on Sexual and Reproductive Health Behavior among Young People.* Inc.(JSI), 2007.

1. [↑](#footnote-ref-6)
2. The purpose of this analysis was simply to describe trends in study LGAs (i.e. no adjustments were made to the main analysis based on the findings). [↑](#footnote-ref-7)
3. HMIS data was kindly shared by Adejumoke Gloria Oluwayinka (Measurement and Evaluation Lead, Adolescents 360 Project, Society for Family Health) on 08/09/2020. [↑](#footnote-ref-8)
4. Sexually active girls: those who report having sexual intercourse in the last 12 months. Fecund girls: those who have started menstruating, are not pregnant, and do not report that they are infertile. [↑](#footnote-ref-10)
5. Note added on October 28, 2022: The main difference between DHS mCPR definition and the definition used in our study is that the former includes pregnant girls, infertile girls, and those girls who have not started menstruating. Moreover, DHS only includes unmarried girls who report having had sexual intercourse in the last month, while we consider all unmarried girls reporting sexual intercourse in the last 12 months. [↑](#footnote-ref-11)
6. Unit of concern may also be called study unit, or sample unit [↑](#footnote-ref-12)
7. Married girls are defined as those girls who are married or cohabiting/living with a man as if married. [↑](#footnote-ref-13)
8. Unmarried girls are those are divorced/separated, widow, or never married. [↑](#footnote-ref-14)
9. This analysis plan was revised in June 2021. Due to low exposure in Nigeria and Ethiopia, a binary variable was created to define girls exposed to A360. [↑](#footnote-ref-15)
10. Before-and-after design may also be called ‘pre- and post-test’ design or ‘pre-experimental’ research design [↑](#footnote-ref-16)
11. This analysis plan was revised in June 2021. Due to low exposure in Nigeria and Ethiopia, a binary variable was created to define girls exposed to A360 [↑](#footnote-ref-17)
12. We restricted past use to the last 12 months to decrease recall bias. [↑](#footnote-ref-18)
13. Note on October 28, 2022 : This sample size calculation has been revised and are shown on page 33 of this supplementary material. [↑](#footnote-ref-19)
