# Supplementary material for: Effect and cost-effectiveness of human-centred design-based approaches to increase adolescent uptake of modern contraceptives in Nigeria, Ethiopia and Tanzania: Population-based, quasi-experimental studies
Source: PLOS Glob Public Health. 2023 Oct 18;3(10):e0002347. doi: 10.1371/journal.pgph.0002347 (PMC10584105; doi:10.1371/journal.pgph.0002347)
Supplement: S1 Table — 1 Sexually active girls are those who report having sexual intercourse in the last 12 months. S1 Table presents a description of the secondary outcomes measured for the Adolescents 360 outcome evaluation, aligning with Adolescents 360 Theory of Change components. (DOCX) [file pgph.0002347.s004.docx]

| **Outcome** | **Details** |
| --- | --- |
| Theory of Change component 1: Adolescents use high quality sexual and reproductive health products and services |  |
| Outcome 1 Proportion of current modern contraceptive users who were using long-acting reversible contraception among sexually active girls ^1^ |  |
| Outcome 2 Use of modern contraceptive within the 12 months before the survey among sexually active girls (mCPR) |  |
| Outcome 3 Age at first birth among girls who gave birth |  |
| Outcome 4 Births in last 12 months | Adolescent girls were divided into two categories, those who gave birth in the 12 months before the survey, and those who did not |
| Outcome 5 Unmet need for modern contraception among sexually active girls | Created by adding unmet need for spacing and unmet need for limiting; unmet need for spacing includes 1) pregnant women whose pregnancy was mistimed, 2) fecund women who are non-pregnant, who are not using any modern method of contraception, and say they want to wait two or more years for their first/next birth, and 3) postpartum amenorrheic women, who are not using any modern method of contraception, and say at the time they became pregnant they had wanted to delay pregnancy; unmet need for limiting refers to1) pregnant women whose pregnancy was unwanted, 2) fecund women who are non-pregnant, who are not using any modern method of contraception, and want no more children, and 3) postpartum amenorrheic women, who are not using any modern method of contraception, and say at the time they became pregnant they had not wanted any more children |
| Theory of Change component 2: Adolescent girls have access to appropriate high quality sexual and reproductive health information and services |  |
| Outcome 6 Awareness of contraceptive products | Sexually active girls were divided into two categories, those who answered ‘Yes’ to the question ‘Have you ever heard of contraceptives?’, and those who did not |
| Outcome 7 Awareness of where to obtain health services | Sexually active girls who were not currently using a contraceptive method (traditional or modern) but intended to use one in the future, were divided into two categories, those who answered ‘Yes’ to the question ‘Do you know of a place where or person from whom you would feel comfortable getting family planning services and products to delay or avoid getting pregnant?’, and those who did not |
| Theory of Change component 3: Contraception positioned as relevant and valuable for adolescent girls |  |
| Outcome 8 Future aspirations index (0-10) | Created using four statements among girls who heard of contraceptives; ‘I have goals for my life’ (2 strongly agree, 1 agree, 0 disagree or strongly disagree), ‘I believe I have some tools to help me achieve my goals for my life’ (2 strongly agree, 1 agree, 0 disagree or strongly disagree), ‘I have little control over the things that happen to me’ (0 strongly agree or agree, 1 disagree, 2 strongly disagree), and ‘I believe preventing unintended pregnancy is important to help me achieve my goals for life’ (4 strongly agree, 3 agree, 0 disagree or strongly disagree); greater scores more desirable than lower scores |
| Outcome 9 Benefit 1 of modern contraception | Girls who heard of contraceptives were divided into two categories, those who agreed with the sentence ‘Using modern contraception can allow an adolescent woman girl to complete her education, find a better job and have a better life’, and those who disagreed |
| Outcome 10 Benefit 2 of modern contraception | Girls who heard of contraceptives were divided into two categories, those who agreed with the sentence ‘Using modern contraception can allow a girl to achieve her life goals’, and those who disagreed |
| Outcome 11 Intention to use a modern method | Sexually active girls who were not using a modern method at the time of the survey were divided into two categories, those who intended to use a method, and those who did not |
| Theory of Change component 4: Supportive environment for adolescent girls to access services |  |
| Outcome 12 Attitudes towards the use of modern contraceptives index (0-2) | Created using two questions among girls who heard of contraceptives; ‘Do you approve or disapprove of married couples using a modern contraceptive method to avoid or delay pregnancy?’ (1 approve, 0 disapprove) and ‘Do you approve or disapprove of couples who are not married using a modern contraceptive method to avoid or delay pregnancy?’ (1 approve, 0 disapprove); greater scores more desirable than lower scores |
| Outcome 13 Self-efficacy to use modern contraceptives index (0-4) | Created using four statements among girls who heard of contraceptives; whether she felt able to start a conversation with her partner about contraception (1 agree, 0 disagree), felt able to use a method of contraception even if her partner did not want her to (1 agree, 0 disagree), felt able to obtain information on contraception services and products if she needed to (1 agree, 0 disagree), and felt able to obtain a contraception method if she decided to use one (1 agree, 0 disagree); greater scores more desirable than lower scores |
| Outcome 14 Descriptive norms index (0-6) | Created using three questions among girls who heard of contraceptives; ‘How many married girls aged 15-19 years in your community do you believe discuss using a method of contraception with their boyfriend or partner/husband or partner?’ (2 most of them, 2 less than half of them, 0 none of them), ‘How many married girls aged 15-19 years in your community do you believe use contraceptive methods?’ (2 most of them, 1 less than half of them, 0 none of them) and ‘How many married girls aged 15-19 years in your community do you believe use contraceptive methods in secrecy from their husband or partner?’ (2 most of them, 1 less than half of them, 0 none of them); greater scores more desirable than lower scores |
| Outcome 15 Community acceptance index (0-2) | Created using two questions among sexually active girls (in last 12 months) who heard of contraceptives; ‘Does your husband approve or disapprove of girls your age using a modern contraceptive method to avoid or delay pregnancy?’ (1 approve, 0 disapprove) and ‘Does your community as a whole approve or disapprove of girls your age using a modern contraceptive method to avoid or delay pregnancy?’ (1 approve, 0 disapprove); greater scores more desirable than lower scores |
| Theory of Change component 5: Trust and credibility of family planning products |  |
| Outcome 16 Misconceptions about contraceptives index (0-3) | Created using three variables among sexually active girls (in last 12 months) who heard of contraceptives; ‘Some modern contraception can stop an adolescent woman from ever being pregnant again even after she stops using it’ (0 agree, 1 disagree), ‘If a modern contraception changes an adolescent woman’s menstrual bleeding, it is bad for her health and can harm her womb’ (0 agree, 1 disagree) and ‘Some modern contraceptives can make adolescent women permanently fat’ (0 agree, 1 disagree); greater scores more desirable than lower scores |
| Outcome 17 Modern contraceptives disadvantages index (0-7) | Number of disadvantages/negative consequences of using modern contraceptive methods mentioned by girls who heard of contraceptives; greater scores less desirable than lower scores |
| Theory of Change component 6: Family planning services available for adolescent girls |  |
| Theory of Change component 7: Adolescent girls sustain use |  |
