# Supplementary material for: Effect and cost-effectiveness of human-centred design-based approaches to increase adolescent uptake of modern contraceptives in Nigeria, Ethiopia and Tanzania: Population-based, quasi-experimental studies
Source: PLOS Glob Public Health. 2023 Oct 18;3(10):e0002347. doi: 10.1371/journal.pgph.0002347 (PMC10584105; doi:10.1371/journal.pgph.0002347)
Supplement: S2 Table — Data are n (%) or mean (SE). 1 p values are for differences between intervention and comparison areas pre-intervention. S2 Table presents a description of demographic characteristics of adolescent girls included in the Adolescents 360 outcome evaluation, pre- and post-intervention, by site. (DOCX) [file pgph.0002347.s005.docx]

| **Characteristics** | **Levels** | **Nasarawa, Nigeria** |  |  |  |  | **Ogun, Nigeria** |  |  |  |  | **Oromia, Ethiopia** |  | **Mwanza, Tanzania** |  |
| --- | --- | --- | --- | --- | --- | --- | --- | --- | --- | --- | --- | --- | --- | --- | --- |
|  |  | **Intervention area** |  | **Comparison area** |  |  | **Intervention area** |  | **Comparison area** |  |  | **Intervention area** |  | **Intervention area** |  |
|  |  | **Pre-intervention** | **Post-intervention** | **Pre-intervention** | **Post-intervention** | **p-value ^1^** | **Pre-intervention** | **Post-intervention** | **Pre-intervention** | **Post-intervention** | **p-value ^1^** | **Pre-intervention** | **Post-intervention** | **Pre-intervention** | **Post-intervention** |
| **Age (years)** |  | 17·54 (0·04) | 18·00 (0·03) | 17·64 (0·03) | 18·18 (0·02) | 0·052 | 16·88 (0·02) | 16·62 (0·03) | 16·89 (0·03) | 16·63 (0·02) | 0·763 | 17·80 (0·06) | 17·81 (0·07) | 16·91 (0·02) | 16·93 (0·03) |
| **Number of living children** | No children | 1,245 (53%) | 1,202 (47%) | 1,083 (44%) | 1,084 (41%) | <0·001 | 5,903 (98%) | 6,807 (99%) | 4,690 (97%) | 6,792 (99%) | 0·024 | 600 (50%) | 493 (44%) | 3,535 (92%) | 4,643 (92%) |
|  | 1 child | 755 (32%) | 1,081 (42%) | 910 (37%) | 1,114 (42%) |  | 114 (2%) | 85 (1%) | 116 (2%) | 58 (1%) |  | 520 (44%) | 592 (48%) | 246 (7%) | 360 (7%) |
|  | 2 children | 296 (13%) | 256 (10%) | 385 (16%) | 411 (16%) |  | 4 (0%) | 5 (0%) | 13 (0%) | 1 (0%) |  | 74 (6%) | 87 (7%) | 29 (1%) | 38 (1%) |
|  | 3 or more children | 46 (2%) | 23 (1%) | 96 (4%) | 28 (1%) |  | 1 (0%) | 1 (0%) | 2 (0%) | 1 (0%) |  | 4 (0%) | 4 (0%) | 1 (0%) | 1 (0%) |
| **Education level** | No education | 642 (27%) | 463 (18%) | 679 (27%) | 374 (14%) | 0·766 | 81 (1%) | 67 (1%) | 60 (1%) | 50 (1%) | 0·540 | 327 (31%) | 331 (32%) | 130 (4%) | 113 (2%) |
|  | Qur'anic only | 61 (3%) | 87 (3%) | 64 (3%) | 154 (6%) |  | 1 (0%) | 0 (0%) | 2 (0%) | 1 (0%) |  | N/A | N/A | N/A | N/A |
|  | Primary | 548 (23%) | 776 (30%) | 625 (25%) | 852 (32%) |  | 263 (4%) | 317 (5%) | 179 (4%) | 281 (4%) |  | 695 (55%) | 641 (51%) | 1,339 (38%) | 1,557 (31%) |
|  | Secondary | 1,039 (44%) | 1,144 (45%) | 1,043 (42%) | 1,169 (44%) |  | 5,407 (90%) | 6,253 (91%) | 4,358 (90%) | 6,290 (92%) |  | 172 (14%) | 191 (16%) | 1,935 (55%) | 3,046 (62%) |
|  | Higher/Technical | 51 (2%) | 91 (4%) | 62 (3%) | 88 (3%) |  | 270 (4%) | 261 (4%) | 221 (5%) | 230 (3%) |  | 4 (0%) | 12 (1%) | 107 (3%) | 327 (7%) |
|  | Don't know | 1 (0%) | 0 (0%) | 1 (0%) | 0 (0%) |  | 0 (0%) | 0 (0%) | 0 (0%) | 0 (0%) |  | 0 (0%) | 0 (0%) | 0 (0%) | 0 (0%) |
| **Religion** | Roman Catholic | 406 (17%) | 363 (14%) | 136 (5%) | 103 (4%) | <0·001 | 125 (2%) | 113 (2%) | 124 (3%) | 104 (2%) | 0·023 | 0 (0%) | 2 (0%) | 1,376 (39%) | 1,793 (36%) |
|  | Orthodox Christian | 0 (0%) | 0 (0%) | 0 (0%) | 0 (0%) |  | 0 (0%) | 0 (0%) | 0 (0%) | 0 (0%) |  | 868 (66%) | 833 (64%) | N/A | N/A |
|  | Protestant/other Christian | 1,002 (43%) | 1,028 (40%) | 939 (38%) | 832 (32%) |  | 3,596 (60%) | 4,201 (61%) | 3,036 (63%) | 4,464 (65%) |  | 106 (8%) | 117 (8%) | 1,554 (44%) | 2,392 (47%) |
|  | Muslim | 918 (39%) | 1,152 (45%) | 1,392 (56%) | 1,684 (64%) |  | 2,274 (38%) | 2,559 (37%) | 1,645 (34%) | 2,267 (33%) |  | 205 (25%) | 208 (27%) | 581 (17%) | 813 (16%) |
|  | Traditional | 14 (1%) | 10 (0%) | 5 (0%) | 18 (1%) |  | 23 (0%) | 21 (0%) | 14 (0%) | 16 (0%) |  | 18 (1%) | 16 (1%) | N/A | N/A |
|  | No religion | 2 (0%) | 8 (0%) | 2 (0%) | 0 (0%) |  | 0 (0%) | 4 (0%) | 1 (0%) | 1 (0%) |  | 1 (0%) | 0 (0%) | 10 (0%) | 6 (0%) |
|  | Don't know | 0 (0%) | 0 (0%) | 0 (0%) | 0 (0%) |  | 2 (0%) | 0 (0%) | 1 (0%) | 0 (0%) |  | 0 (0%) | 0 (0%) | 0 (0%) | 39 (1%) |
| **Wealth quintile** | Lowest quintile | 293 (13%) | 332 (13%) | 217 (9%) | 244 (10%) | <0·001 | 2 (0%) | 1 (0%) | 19 (0%) | 4 (0%) | 0·004 | 321 (33%) | 183 (22%) | 329 (13%) | 501 (12%) |
|  | 2nd quintile | 460 (20%) | 399 (16%) | 480 (20%) | 651 (26%) |  | 16 (0%) | 24 (0%) | 34 (1%) | 10 (0%) |  | 144 (13%) | 135 (12%) | 596 (23%) | 819 (20%) |
|  | 3rd quintile | 370 (16%) | 429 (17%) | 634 (27%) | 722 (28%) |  | 147 (3%) | 226 (3%) | 117 (2%) | 190 (3%) |  | 147 (13%) | 172 (14%) | 354 (14%) | 758 (18%) |
|  | 4th quintile | 477 (21%) | 481 (19%) | 606 (26%) | 628 (25%) |  | 940 (16%) | 1,721 (25%) | 825 (17%) | 1,407 (21%) |  | 197 (16%) | 290 (25%) | 624 (24%) | 1,260 (30%) |
|  | Highest quintile | 656 (29%) | 856 (34%) | 418 (18%) | 302 (12%) |  | 4,738 (81%) | 4,894 (71%) | 3,732 (79%) | 5,214 (76%) |  | 351 (26%) | 374 (26%) | 693 (27%) | 849 (20%) |
| **Mobile phone access** | Owns smartphone | 419 (18%) | 796 (31%) | 294 (12%) | 378 (14%) | 0·002 | 2,415 (40%) | 2,643 (38%) | 1,843 (38%) | 2,937 (43%) | <0·001 | 50 (4%) | 134 (10%) | 367 (10%) | 502 (10%) |
|  | Owns non-smart mobile phone | 846 (36%) | 1,170 (46%) | 1,027 (42%) | 1,571 (60%) |  | 1,146 (19%) | 1,944 (28%) | 737 (15%) | 1,776 (26%) |  | 422 (33%) | 395 (33%) | 879 (25%) | 1,465 (29%) |
|  | Accesses mobile phone at least once a week | 53 (2%) | 190 (7%) | 57 (2%) | 252 (10%) |  | 336 (6%) | 1,218 (18%) | 621 (13%) | 1,054 (15%) |  | 183 (15%) | 287 (24%) | 268 (8%) | 782 (16%) |
|  | Accesses mobile phone less than once a week | 77 (3%) | 95 (4%) | 93 (4%) | 47 (2%) |  | 522 (9%) | 216 (3%) | 435 (9%) | 241 (4%) |  | 106 (8%) | 127 (12%) | 419 (12%) | 296 (6%) |
|  | No mobile phone access | 945 (40%) | 311 (12%) | 1,000 (40%) | 389 (15%) |  | 1,602 (27%) | 876 (13%) | 1,182 (25%) | 844 (12%) |  | 436 (40%) | 223 (21%) | 1,578 (45%) | 1,997 (40%) |
|  | Don’t know | 2 (0%) | 0 (0%) | 2 (0%) | 0 (0%) |  | 1 (0%) | 0 (0%) | 2 (0%) | 0 (0%) |  | 0 (0%) | 0 (0%) | 0 (0%) | 1 (0%) |
