# Supplementary material for: Effect and cost-effectiveness of human-centred design-based approaches to increase adolescent uptake of modern contraceptives in Nigeria, Ethiopia and Tanzania: Population-based, quasi-experimental studies
Source: PLOS Glob Public Health. 2023 Oct 18;3(10):e0002347. doi: 10.1371/journal.pgph.0002347 (PMC10584105; doi:10.1371/journal.pgph.0002347)
Supplement: S3 Table — mCPR, modern contraceptive prevalence rate, LARC, long-acting reversible contraceptive, Data are n (%) or mean (SE). 1 Girls who agreed with the sentence ‘Using modern contraception can allow an adolescent woman girl to complete her education, find a better job and have a better life’ 2 The impact of the Adolescents 360 approach is defined as the risk of mCPR pre- versus post-intervention. S3 Table presents a description of primary and secondary outcomes measured for the Adolescents 360 outcome evaluation, pre- and post-intervention, by site. This table also presents the impact of the Adolescents 360 approach in each site. (DOCX) [file pgph.0002347.s006.docx]

| **Outcomes** | **Intervention area** |  | **Comparison area** |  |  | **Analytic results** |  |  |
| --- | --- | --- | --- | --- | --- | --- | --- | --- |
|  | **Pre-intervention** | **Post-intervention** | **Pre-intervention** | **Post-intervention** | **Effect** ^2^ | **Effect (95%CI)** | **p-value** | **n** |
| **Nasarawa, Nigeria** |  |  |  |  |  |  |  |  |
| mCPR | 210/1,280 (16%) | 537/1,404 (38%) | 180/1,336 (13%) | 422/1,560 (27%) | 1·06 | 0·96 (0·76 to 1·21) | 0·738 | 5,414 |
| Proportion of current modern contraceptive users using a LARC | 55/210 (26%) | 165/537 (31%) | 30/180 (17%) | 133/422 (32%) | 0·62 | 0·66 (0·41 to 1·06) | 0·086 | 1,332 |
| Use of a modern method in last 12 months | 221/1,280 (17%) | 549/1,404 (39%) | 196/1,335 (15%) | 444/1,560 (28%) | 1·17 | 0·97 (0·78 to 1·22) | 0·821 | 5,413 |
| Age at first birth | 16·39 (0·06) | 16·91 (0·04) | 16·18 (0·06) | 16·84 (0·04) | -0·13 | -0·07 (-0·23 to 0·10) | 0·448 | 5,426 |
| Birth in last 12 months | 531/2,342 (23%) | 1,094/2,562 (43%) | 580/2,474 (23%) | 1,210/2,637 (46%) | 0·96 | 1·00 (0·87 to 1·14) | 0·959 | 9,651 |
| Unmet need | 426/2,029 (21%) | 491/2,418 (20%) | 490/2,151 (23%) | 531/2,418 (22%) | 1·00 | 0·99 (0·83 to 1·18) | 0·909 | 8,687 |
| Awareness of contraceptive products | 1,185/2,339 (51%) | 2,074/2,562 (81%) | 1,087/2,469 (44%) | 2,062/2,635 (78%) | 0·90 | 0·88 (0·80 to 0·96) | 0·006 | 9,643 |
| Awareness of where to obtain health services | 202/442 (46%) | 479/523 (92%) | 230/435 (53%) | 575/639 (90%) | 1·18 | 1·13 (0·97 to 1·31) | 0·116 | 1,998 |
| Benefit 1 of modern contraception ^1^ | 1,070/1,185 (90%) | 1,949/2,074 (94%) | 995/1,087 (92%) | 1,852/2,062 (90%) | 1·06 | 1·05 (1·01 to 1·09) | 0·009 | 6,255 |
| Intention to use a method | 815/1,217 (67%) | 1,512/1,792 (84%) | 759/1,190 (64%) | 1,331/1,662 (80%) | 1·00 | 0·98 (0·90 to 1·07) | 0·639 | 5,738 |
| Attitudes index score (0-2) | 1·27 (0·03) | 1·51 (0·02) | 1·11 (0·03) | 1·22 (0·02) | 0·12 | 0·10 (0·01 to 0·18) | 0·023 | 6,255 |
| Self-efficacy index score (0-4) | 1·43 (0·06) | 2·16 (0·06) | 1·36 (0·06) | 1·82 (0·06) | 0·27 | 0·10 (-0·11 to 0·30) | 0·363 | 5,423 |
| Community acceptance index score (0-2) | 0·78 (0·03) | 1·24 (0·03) | 0·67 (0·03) | 1·02 (0·03) | 0·12 | 0·07 (-0·04 to 0·17) | 0·212 | 6,212 |
| Misconceptions about modern contraceptives index score (0-3) | 0·76 (0·04) | 0·68 (0·03) | 0·69 (0·04) | 0·6 (0·03) | 0·00 | -0·05 (-0·12 to 0·02) | 0·196 | 5,643 |
| **Ogun, Nigeria** |  |  |  |  |  |  |  |  |
| mCPR | 346/763 (45%) | 360/738 (49%) | 485/959 (51%) | 413/810 (51%) | 1·03 | 1·08 (0·92 to 1·26) | 0·340 | 3,230 |
| Proportion of current modern contraceptive users using a LARC | 1/346 (0%) | 8/360 (2%) | 7/485 (1%) | 4/413 (1%) | 11·46 | 13·32 (1·44 to 123·09) | 0·022 | 1,593 |
| Use of a modern method in last 12 months | 355/763 (47%) | 374/738 (51%) | 504/958 (53%) | 441/810 (54%) | 1·05 | 1·06 (0·91 to 1·24) | 0·422 | 3,229 |
| Age at first birth | 17·25 (0·13) | 17·21 (0·13) | 17·18 (0·11) | 17·09 (0·17) | 0·05 | 0·17 (-0·30 to 0·65) | 0·472 | 437 |
| Birth in last 12 months | 36/6,043 (1%) | 76/6,897 (1%) | 54/6,010 (1%) | 47/6,851 (1%) | 2·42 | 2·12 (1·20 to 3·76) | 0·010 | 25,436 |
| Unmet need | 275/821 (34%) | 193/774 (25%) | 325/1,025 (32%) | 216/842 (26%) | 0·92 | 0·92 (0·73 to 1·15) | 0·467 | 3,419 |
| Awareness of contraceptive products | 4,618/6,027 (77%) | 4,863/6,898 (71%) | 4,929/6,002 (82%) | 5,155/6,852 (75%) | 1·00 | 1·01 (0·95 to 1·07) | 0·777 | 25,414 |
| Awareness of where to obtain health services | 60/185 (32%) | 92/197 (47%) | 104/288 (36%) | 100/210 (48%) | 1·09 | 1·03 (0·74 to 1·44) | 0·857 | 860 |
| Benefit 1 of modern contraception | 3,449/4,618 (75%) | 3,396/4,862 (70%) | 3,685/4,929 (75%) | 3,886/5,154 (75%) | 0·93 | 0·93 (0·88 to 0·97) | 0·001 | 19,341 |
| Intention to use a method | 546/704 (78%) | 545/828 (66%) | 648/879 (74%) | 587/890 (66%) | 0·95 | 0·98 (0·89 to 1·08) | 0·718 | 3,097 |
| Attitudes index score (0-2) | 1·18 (0·02) | 1·12 (0·02) | 1·13 (0·02) | 1·17 (0·02) | -0·10 | -0·09 (-0·16 to -0·03) | 0·003 | 19,343 |
| Self-efficacy index score (0-4) | 2·72 (0·06) | 2·37 (0·09) | 2·76 (0·05) | 2·57 (0·07) | -0·16 | -0·15 (-0·42 to 0·12) | 0·274 | 3,238 |
| Community acceptance index score (0-2) | 0·28 (0·02) | 0·48 (0·03) | 0·34 (0·02) | 0·54 (0·03) | 0·01 | 0·00 (-0·11 to 0·10) | 0·977 | 3,087 |
| Misconceptions about modern contraceptives index score (0-3) | 0·77 (0·03) | 0·72 (0·04) | 0·82 (0·03) | 0·68 (0·03) | 0·10 | 0·10 (-0·03 to 0·22) | 0·125 | 4,360 |
| **Oromia, Ethiopia** |  |  |  |  |  |  |  |  |
| mCPR | 559/846 (61%) | 565/854 (61%) | N/A | N/A | -0·01 | 0·05 (0·01 to 0·10) | 0·025 | 112 |
| Proportion of current modern contraceptive users using a LARC | 89/559 (16%) | 140/565 (26%) | N/A | N/A | 0·10 | 0·10 (0·03 to 0·17) | 0·004 | 109 |
| Use of a modern method in last 12 months | 621/846 (68%) | 595/851 (64%) | N/A | N/A | -0·04 | 0·02 (-0·02 to 0·07) | 0·290 | 112 |
| Age at first birth | 17·00 (0·06) | 16·89 (0·09) | N/A | N/A | -0·11 | -0·06 (-0·29 to 0·16) | 0·590 | 111 |
| Birth in last 12 months | 282/1,198 (25%) | 287/1,176 (25%) | N/A | N/A | 0·00 | 0·02 (-0·04 to 0·09) | 0·436 | 114 |
| Unmet need | 199/1,044 (21%) | 196/1,002 (21%) | N/A | N/A | 0·00 | 0·01 (-0·04 to 0·06) | 0·626 | 114 |
| Awareness of contraceptive products | 1099/1,198 (90%) | 1,072/1,176 (90%) | N/A | N/A | 0·00 | 0·11 (0·01 to 0·21) | 0·025 | 114 |
| Awareness of where to obtain health services | 177/200 (88%) | 168/178 (93%) | N/A | N/A | 0·05 | 0·07 (0·00 to 0·13) | 0·058 | 35 |
| Benefit 1 of modern contraception | 965/1,064 (90%) | 1032/1,055 (98%) | N/A | N/A | 0·08 | 0·09 (0·02 to 0·16) | 0·014 | 113 |
| Intention to use a method | 201/277 (67%) | 176/243 (68%) | N/A | N/A | 0·01 | 0·05 (-0·06 to 0·16) | 0·393 | 100 |
| Attitudes index score (0-2) | 1·46 (0·04) | 1·66 (0·04) | N/A | N/A | 0·19 | 0·22 (0·08 to 0·36) | 0·003 | 113 |
| Self-efficacy index score (0-4) | 2·98 (0·08) | 3·70 (0·05) | N/A | N/A | 0·72 | 0·70 (0·45 to 0·94) | <0·001 | 114 |
| Community acceptance index score (0-2) | 1·23 (0·07) | 1·58 (0·05) | N/A | N/A | 0·35 | 0·29 (0·09 to 0·49) | 0·005 | 114 |
| Misconceptions about modern contraceptives index score (0-3) | 1·3 (0·05) | 1·27 (0·07) | N/A | N/A | -0·04 | -0·06 (-0·19 to 0·07) | 0·354 | 113 |
| **Mwanza, Tanzania** |  |  |  |  |  |  |  |  |
| mCPR | 385/758 (51%) | 505/1,215 (42%) | N/A | N/A | -0·09 | -0·09 (-0·17 to -0·003) | 0·043 | 60 |
| Proportion of current modern contraceptive users using a LARC | 65/401 (16%) | 123/532 (23%) | N/A | N/A | 0·07 | 0·09 (0·02 to 0·16) | 0·019 | 44 |
| Use of a modern method in last 12 months | 424/758 (56%) | 602/1,215 (50%) | N/A | N/A | 0·06 | 0·35 (-0·27 to 0·96) | 0·251 | 56 |
| Age at first birth | 16·52 (0·07) | 16·81 (0·06) | N/A | N/A | 0·29 | 0·47 (-0·01 to 0·93) | 0·052 | 60 |
| Birth in last 12 months | N/A | N/A | N/A | N/A | N/A | N/A | N/A | N/A |
| Unmet need | 356/932 (38%) | 508/1,413 (36%) | N/A | N/A | -0·02 | -0·04 (-0·09 to 0·02) | 0·193 | 59 |
| Awareness of contraceptive products | 2,852/3,511 (81%) | 4,793/5,043 (95%) | N/A | N/A | 0·14 | 0·13 (0·11 to 0·16) | <0·001 | 59 |
| Awareness of where to obtain health services | 470/807 (58%) | 376/626 (60%) | N/A | N/A | 0·02 | -0·02 (-0·11 to 0·07) | 0·599 | 57 |
| Benefit 1 of modern contraception | 2,481/2,852 (87%) | 4,033/4,793 (84%) | N/A | N/A | -0·03 | -0·05 (-0·09 to -0·03) | <0·001 | 60 |
| Intention to use a method | 373/497 (75%) | 615/843 (73%) | N/A | N/A | -0·02 | -0·14 (-0·24 to -0·04) | 0·008 | 58 |
| Attitudes index score (0-2) | 1·47 (0·01) | 1·32 (0·01) | N/A | N/A | -0·15 | -0·14 (-0·24 to -0·05) | 0·007 | 58 |
| Self-efficacy index score (0-4) | 3·34 (0·03) | 3·15 (0·03) | N/A | N/A | -0·18 | -0·36 (-0·55 to -0·17) | <0·001 | 58 |
| Community acceptance index score (0-2) | N/A | N/A | N/A | N/A | N/A | N/A | N/A | N/A |
| Misconceptions about modern contraceptives index score (0-3) | 0·81 (0·02) | 0·89 (0·01) | N/A | N/A | 0·08 | 0·05 (0·02 to 0·19) | 0·459 | 58 |
