# Supplementary material for: Effect and cost-effectiveness of human-centred design-based approaches to increase adolescent uptake of modern contraceptives in Nigeria, Ethiopia and Tanzania: Population-based, quasi-experimental studies
Source: PLOS Glob Public Health. 2023 Oct 18;3(10):e0002347. doi: 10.1371/journal.pgph.0002347 (PMC10584105; doi:10.1371/journal.pgph.0002347)
Supplement: S4 Table — A360, Adolescents 360 approach, mCPR, modern contraceptive prevalence rate, LARC, long-acting reversible contraceptive, Data are n (%) or mean (SE). 1 Girls who agreed with the sentence ‘Using modern contraception can allow an adolescent woman girl to complete her education, find a better job and have a better life’ 2 Girls who agreed with the sentence ‘Using modern contraception can allow a girl to achieve her life goals’ 3 The impact of the A360 exposure is defined as the risk of mCPR in the exposed compared to girls not exposed to A360. S4 Table presents a description of primary and secondary outcomes measured for the Adolescents 360 outcome evaluation, by girls who reported being exposed to the intervention and girls who did not report being exposed to the intervention, by site. (DOCX) [file pgph.0002347.s007.docx]

| **Outcomes** | **Reported being exposed to A360** | **Reported not being exposed to A360** | **Effect** ^3^ | **Effect (95%CI)** | **p-value** | **n** |
| --- | --- | --- | --- | --- | --- | --- |
| **Nasarawa, Nigeria** |  |  |  |  |  |  |
| mCPR | 50/98 (51%) | 487/1,326 (37%) | 1·68 | 1·43 (1·15 to 1·79) | 0·002 | 1,371 |
| Proportion of current modern contraceptive users using a LARC | 20/50 (40%) | 145/487 (30%) | 1·34 | 1·25 (0·87 to 1·81) | 0·231 | 532 |
| Use of a modern method in last 12 months | 53/98 (54%) | 496/1,326 (37%) | 1·83 | 1·50 (1·22 to 1·83) | <0·001 | 1,373 |
| Age at first birth | 16·80 (0·14) | 16·92 (0·04) | -0·13 | -0·15 (-0·38 to 0·08) | 0·192 | 1,358 |
| Birth in last 12 months | 73/142 (51%) | 1,021/2,420 (42%) | 1·22 | 1·18 (1·01 to 1·40) | 0·043 | 2,495 |
| Unmet need | 18/135 (13%) | 473/2,283 (21%) | 0·64 | 0·60 (0·39 to 0·94) | 0·024 | 2,357 |
| Awareness of contraceptive products | 137/142 (96%) | 1,937/2,420 (80%) | 1·21 | 1·18 (1·11 to 1·25) | <0·001 | 2,495 |
| Awareness of where to obtain health services | 32/32 (100%) | 447/491 (91%) | 1·10 | 1·11 (1·07 to 1·16) | <0·001 | 517 |
| Aspirations index score (0-9) | 6·32 (0·20) | 5·68 (0·08) | 0·64 | 0·56 (0·13 to 0·99) | 0·011 | 2,100 |
| Benefit 1 of modern contraception ^1^ | 134/137 (98%) | 1,815/1,937 (94%) | 1·04 | 1·05 (1·02 to 1·08) | 0·001 | 2,027 |
| Benefit 2 of modern contraception ^2^ | 131/136 (96%) | 1,796/1,898 (95%) | 1·02 | 1·02 (0·99 to 1·06) | 0·190 | 1,989 |
| Intention to use a method | 97/98 (99%) | 1,415/1,694 (84%) | 1·18 | 1·20 (1·15 to 1·25) | <0·001 | 1,777 |
| Attitudes index score (0-2) | 1·66 (0·04) | 1·50 (0·02) | 0·17 | 0·20 (0·10 to 0·30) | <0·001 | 2,027 |
| Self-efficacy index score (0-4) | 2·24 (0·18) | 2·15 (0·06) | 0·09 | 0·11 (-0·19 to 0·42) | 0·461 | 1,374 |
| Descriptive norms index score (0-6) | 3·77 (0·16) | 3·34 (0·07) | 0·42 | 0·44 (0·14 to 0·73) | 0·004 | 1,779 |
| Community acceptance index score (0-2) | 1·28 (0·08) | 1·24 (0·03) | 0·03 | 0·03 (-0·11 to 0·17) | 0·667 | 1,779 |
| Misconceptions about modern contraceptives index score (0-3) | 0·69 (0·11) | 0·68 (0·03) | 0·02 | 0·02 (-0·19 to 0·22) | 0·877 | 1,779 |
| Disadvantages of contraceptives index score (0-7) | 1·88 (0·13) | 1·63 (0·04) | 0·25 | 0·24 (-0·02 to 0·49) | 0·068 | 1,779 |
| **Ogun, Nigeria** |  |  |  |  |  |  |
| mCPR | 22/54 (41%)/ | 338/684 (49%) | 0·72 | 0·82 (0·59 to 1·14) | 0·234 | 736 |
| Proportion of current modern contraceptive users using a LARC | 0/22 (0%) | 8/338 (2%) | 0·00 | - |  |  |
| Use of a modern method in last 12 months | 23/54 (43%) | 351/684 (51%) | 0·72 | 0·82 (0·61 to 1·11) | 0·206 | 736 |
| Age at first birth | 17·86 (0·38) | 17·16 (0·14) | 0·70 | 0·58 (-0·24 to 1·40) | 0·162 | 95 |
| Birth in last 12 months | 5/86 (6%) | 71/1,056 (7%) | 0·86 | 0·87 (0·37 to 2·04) | 0·756 | 1,138 |
| Unmet need | 18/56 (32%) | 175/718 (24%) | 1·32 | 1·32 (0·89 to 1·96) | 0·162 | 772 |
| Awareness of contraceptive products | 78/86 (91%) | 881/1,056 (83%) | 1·09 | 1·07 (1·00 to 1·15) | 0·063 | 1,138 |
| Awareness of where to obtain health services | 8/17 (47%) | 84/180 (47%) | 1·01 | 1·01 (0·62 to 1·65) | 0·967 | 195 |
| Aspirations index score (0-9) | 7·13 (0·16) | 6·95 (0·06) | 0·18 | 0·13 (-0·20 to 0·47) | 0·437 | 966 |
| Benefit 1 of modern contraception ^1^ | 69/78 (88%) | 760/881 (86%) | 1·03 | 1·02 (0·93 to 1·12) | 0·648 | 955 |
| Benefit 2 of modern contraception ^2^ | 67/77 (87%) | 732/870 (84%) | 1·03 | 1·03 (0·94 to 1·13) | 0·535 | 943 |
| Intention to use a method | 39/69 (57%) | 506/759 (67%) | 0·85 | 0·85 (0·67 to 1·06) | 0·129 | 824 |
| Attitudes index score (0-2) | 1·33 (0·08) | 1·4 (0·03) | -0·07 | -0·08 (-0·25 to 0·08) | 0·331 | 955 |
| Self-efficacy index score (0-4) | 2·65 (0·23) | 2·35 (0·09) | 0·30 | 0·28 (-0·20 to 0·76) | 0·246 | 736 |
| Descriptive norms index score (0-6) | 2·91 (0·21) | 3·09 (0·09) | -0·18 | -0·20 (-0·64 to 0·24) | 0·366 | 826 |
| Community acceptance index score (0-2) | 0·63 (0·11) | 0·47 (0·03) | 0·15 | 0·18 (-0·05 to 0·41) | 0·125 | 586 |
| Misconceptions about modern contraceptives index score (0-3) | 0·67 (0·12) | 0·72 (0·04) | -0·05 | -0·04 (-0·29 to 0·21) | 0·753 | 826 |
| Disadvantages of contraceptives index score (0-7) | 1·13 (0·17) | 1·3 (0·05) | -0·17 | -0·18 (-0·51 to 0·15) | 0·289 | 826 |
| **Oromia, Ethiopia** |  |  |  |  |  |  |
| mCPR | 177/217 (80%) | 388/637 (55%) | 1·47 | 2·09 (1·32 to 3·29) | 0·002 | 801 |
| Proportion of current modern contraceptive users using a LARC | 60/177 (37%) | 80/388 (21%) | 1·76 | 1·52 (0·75 to 3·08) | 0·240 | 482 |
| Use of a modern method in last 12 months | 184/217 (84%) | 411/634 (58%) | 1·45 | 1·77 (1·08 to 2·92) | 0·026 | 759 |
| Age at first birth | 16·91 (0·11) | 16·85 (0·09) | 0·06 | 0·07 (-0·16 to 0·30) | 0·542 | 652 |
| Birth in last 12 months | 78/285 (28%) | 202/861 (23%) | 1·21 | 1·74 (1·11 to 2·74) | 0·018 | 1,089 |
| Unmet need | 34/251 (14%) | 160/744 (23%) | 0·63 | 0·49 (0·26 to 0·94) | 0·033 | 845 |
| Awareness of contraceptive products | 279/285 (98%) | 766/861 (88%) | 1·12 | 3·33 (0·96 to 11·48) | 0·057 | 768 |
| Awareness of where to obtain health services | 27/28 (95%) | 141/150 (93%) | 1·02 | - |  |  |
| Aspirations index score (0-9) | 5·58 (0·09) | 5·44 (0·09) | 0·13 | 0·13 (-0·13 to 0·39) | 0·312 | 1,111 |
| Benefit 1 of modern contraception ^1^ | 278/279 (100%) | 727/749 (97%) | 1·03 | - |  |  |
| Benefit 2 of modern contraception ^2^ | 274/279 (98%) | 725/756 (95%) | 1·03 | 0·85 (0·17 to 4·21) | 0·830 | 397 |
| Intention to use a method | 29/37 (79%) | 150/210 (67%) | 1·18 | 0·36 (0·12 to 1·14) | 0·214 | 168 |
| Attitudes index score (0-2) | 1·77 (0·03) | 1·62 (0·05) | 0·15 | 0·08 (0·00 to 0·16) | 0·061 | 1,011 |
| Self-efficacy index score (0-4) | 3·86 (0·04) | 3·65 (0·07) | 0·21 | 0·01 (-0·14 to 0·16) | 0·871 | 1,050 |
| Descriptive norms index score (0-6) | 2·51 (0·12) | 2·24 (0·13) | 0·27 | 0·15 (-0·15 to 0·46) | 0·321 | 1,019 |
| Community acceptance index score (0-2) | 1·78 (0·05) | 1·5 (0·06) | 0·27 | 0·10 (-0·02 to 0·21) | 0·113 | 1,050 |
| Misconceptions about modern contraceptives index score (0-3) | 1·35 (0·11) | 1·24 (0·07) | 0·11 | 0·23 (-0·03 to 0·49) | 0·079 | 1,019 |
| Disadvantages of contraceptives index score (0-7) | 2·65 (0·35) | 1·57 (0·22) | 1·08 | 0·31 (-0·29 to 0·91) | 0·305 | 1,059 |
| **Mwanza, Tanzania** |  |  |  |  |  |  |
| mCPR | 167/309 (54%) | 338/906 (37%) | 1·45 | 1·63 (1·28 to 2·09) | <0·001 | 1,215 |
| Proportion of current modern contraceptive users using a LARC | 26/167 (16%) | 80/338 (24%) | 0·67 | 0·51 (0·25 to 1·04) | 0·064 | 532 |
| Use of a modern method in last 12 months | 181/309 (59%) | 371/535 (69%) | 0·84 | 1·71 (1·33 to 2·21) | <0·001 | 1,215 |
| Age at first birth | 16·92 (0·11) | 16·78 (0·06) | 0·14 | -0·04 (-0·11 to 0·02) | 0·187 | 421 |
| Birth in last 12 months | N/A | N/A | N/A | N/A | N/A | N/A |
| Unmet need | 100/354 (28%) | 408/1059 (39%) | 0·71 | 0·72 (0·54 to 0·98) | 0·035 | 1,413 |
| Awareness of contraceptive products | 1,162/1,192 (97%) | 3,631/3,849 (94%) | 1·52 | 2·01 (1·16 to 3·49) | 0·013 | 5,041 |
| Awareness of where to obtain health services | 94/132 (71%) | 282/494 (57%) | 1·25 | 1·81 (1·22 to 2·69) | 0·003 | 626 |
| Aspirations index score (0-9) | N/A | N/A | N/A | N/A | N/A | N/A |
| Benefit 1 of modern contraception ^1^ | 1,025/1,130 (91%) | 3,008/3,424 (87%) | 1·03 | 1·27 (0·99 to 1·62) | 0·051 | 3,839 |
| Benefit 2 of modern contraception ^2^ | 937/1,124 (81%) | 2,743/3,415 (76%) | 1·04 | 1·18 (0·95 to 1·46) | 0·134 | 3,840 |
| Intention to use a method | 129/170 (76%) | 486/662 (73%) | 1·03 | 1·08 (0·64 to 1·82) | 0·781 | 832 |
| Attitudes index score (0-2) | 1·42 (0·02) | 1·28 (0·01) | 0·04 | 0·00 (-0·01 to 0·13) | 0·388 | 3,947 |
| Self-efficacy index score (0-4) | 3·31 (0·05) | 3·12 (0·03) | 0·19 | 0·01 (0·00 to 0·02) | 0·010 | 1,219 |
| Descriptive norms index score (0-6) | 4·13 (0·089) | 3·95 (0·06) | 0·18 | -0·03 (-0·08 to 0·02) | 0·238 | 1,137 |
| Community acceptance index score (0-2) | N/A | N/A | N/A | N/A | N/A | N/A |
| Misconceptions about modern contraceptives index score (0-3) | 0·96 (0·03) | 0·86 (0·02) | 0·1 | 0·01 (-0·01 to 0·02) | 0·314 | 3,750 |
| Disadvantages of contraceptives index score (0-7) | N/A | N/A | N/A | N/A | N/A | N/A |
