# Supplementary material for: Effect and cost-effectiveness of human-centred design-based approaches to increase adolescent uptake of modern contraceptives in Nigeria, Ethiopia and Tanzania: Population-based, quasi-experimental studies
Source: PLOS Glob Public Health. 2023 Oct 18;3(10):e0002347. doi: 10.1371/journal.pgph.0002347 (PMC10584105; doi:10.1371/journal.pgph.0002347)
Supplement: Attachment — Submitted filename: Comments to the authors.pdf [file pgph.0002347.s008.pdf]

Report for "Effect and cost-effectiveness of human-centered design-based approaches to increase adolescent uptake of modern contraceptives in Nigeria, Ethiopia and Tanzania: population-based, quasi-experimental studies"

In this paper, the authors study the impacts Adolescents 360 (A360) program on the prevalence of modern contraceptive use among women aged 15-19. Specifically, they study the impacts of four family planning interventions implemented under the A360 iterative design approach in Nigeria, Ethiopia, and Tanzania. In Nigeria, these programs included "9ja Girls", which provided young girls with designated safe spaces and walk-in one-on-one counseling, and "Matasa Matan Arewa," which aimed to co-opt men through male interpersonal communicators. In Tanzania, the "Smart Start" program approached new couples and used general family planning discussions as an entry point to discuss contraceptives. In Ethiopia, the "Be Smart" program offered women life skills and entrepreneurship training with an opt-out family planning counseling.

The authors utilize data from cross-sectional ex-ante and ex-post households conducted in each program area. For the two programs conducted in Nigeria, the authors also surveyed designated comparison areas. In Nigeria, the authors use a difference-in-difference style analysis to estimate the impacts of the programs on modern contraceptive use. In the other settings, where there is no control group, the authors utilize a pre-post analysis. In none of the settings do the authors find evidence of an impact on contraceptive use. Finally, the authors extend their estimates to a cost analysis and find that each program's cost per DALY averted was well beyond the GDP per capita in each setting.

The authors should be congratulated on an impressive amount of work on an important question. Results such as those presented in this paper are important to disseminate because they are comprehensive to an organization's programming strategy and stand in contrast to the positive effects. With that said, I see several ways to improve this work. Below I outline what I hope will be helpful suggestions to improve the comprehensibility of the work and the credibility of the methods.

1. **Organization:** The authors attempt to comprehensively study the A360 program in this work. However, the interventions, settings, and methods are quite different, and space constraints mean that many important details have been relegated to supplementary information. While I recognize these interventions were pre-registered together, I suggest organizing these analyses into two companion papers. In the first, present the analysis of the Nigeria programs. In the other, group the Ethiopia and Tanzania interventions. This would allow for greater exposition of the interventions and internal continuity in methods (more on this later). I suspect the Nigeria paper in this scenario would be the stronger of the two works.
    a. I certainly sympathize with the challenge of describing interventions that have already been published as protocol papers. However, for the sake of internal completeness, I would avoid simply referring to the BMJ open paper for the reader to view the details of the intervention. I suggest moving Table S1 to the main text.
2. **Methods:** The work utilizes two separate empirical strategies, so I will discuss them separately.
    a. **Nigeria (diff-in-diff)**: I find this to be the more convincing of the analyses. However, it is unclear to me how the comparison groups were chosen. I suggest that the authors include a formal test of differences in Table S2 (and potentially move it to the main text). I suspect this analysis will show that the treatment and comparison areas are systematically different. The authors may wish to test the robustness of their estimates to propensity score weighting methods.

    i. I suspect the authors will also face criticism for their lack of discussion of the parallel trends assumption. They may attempt to utilize the Nigeria DHS to analyze trends between enumerated areas, albeit at a higher level.

b. **Ethiopia and Tanzania (Pre-Post Analysis):** With the surveys' pre/post analysis and cross-sectional nature, it is impossible to rule out the possibility that the results are driven by trends unrelated to the A360 program. I suggest that the authors present these results in a separate paper and frame them as descriptive and related to a process evaluation of the A360 program rather than an attempt to estimate the effects of the program.
c. **Common Issues:**
    i. Panel surveys would provide a more convincing analysis of the program. Recognizing that this was not possible, I think providing more description of the sampling protocol is warranted.
    ii. I don't believe the authors can make statements about the program's effectiveness with program exposure so low in each intervention-arm survey (less than 25 percent in each setting if I'm reading correctly). Indeed, in Table 2, the individual-level analysis provides suggestive evidence that the programs were very effective among women who received them. Instead, the low self-reported participation density may represent an implementation or scale issue or low demand for family planning services.
    iii. Relatedly, the individual analysis in Table 2 is subject to selection bias. In Nigeria, the authors may be able to include both intervention and control areas, allowing them to use residence in an intervention area as an instrumental variable for program participation. This may be a weak instrument given the low program uptake, but if not, this would give the authors a less biased estimate of the local average treatment effect.

Some more minor points:

1. In the companion protocol paper, the authors describe a dose-response analysis that I don't believe can be found in this work.
2. The authors conduct their analysis on complete cases. Are these results robust to other methods of dealing with incomplete information on covariates?
3. It is unclear to me that DALYs are the right unit to use when conducting the cost analysis. Instead, I may present the cost per percentage point increase in the mCPR.
