# Supplementary material for: Effect and cost-effectiveness of human-centred design-based approaches to increase adolescent uptake of modern contraceptives in Nigeria, Ethiopia and Tanzania: Population-based, quasi-experimental studies
Source: PLOS Glob Public Health. 2023 Oct 18;3(10):e0002347. doi: 10.1371/journal.pgph.0002347 (PMC10584105; doi:10.1371/journal.pgph.0002347)
Supplement: Attachment — Submitted filename: Response to reviewers.docx [file pgph.0002347.s009.docx]

**Reviewer #1:**

Report for “Effect and cost-effectiveness of human-centered design-based approaches to increase adolescent uptake of modern contraceptives in Nigeria, Ethiopia and Tanzania: population-based, quasiexperimental studies”

In this paper, the authors study the impacts Adolescents 360 (A360) program on the prevalence of modern contraceptive use among women aged 15-19. Specifically, they study the impacts of four family planning interventions implemented under the A360 iterative design approach in Nigeria, Ethiopia, and

Tanzania. In Nigeria, these programs included “9ja Girls”, which provided young girls with designated safe spaces and walk-in one-on-one counseling, and “Matasa Matan Arewa,” which aimed to co-opt men through male interpersonal communicators. In Tanzania, the “Smart Start” program approached new couples and used general family planning discussions as an entry point to discuss contraceptives. In Ethiopia, the “Be Smart” program offered women life skills and entrepreneurship training with an opt-out family planning counseling.

The authors utilize data from cross-sectional ex-ante and ex-post households conducted in each program area. For the two programs conducted in Nigeria, the authors also surveyed designated comparison areas. In Nigeria, the authors use a difference-in-difference style analysis to estimate the impacts of the programs on modern contraceptive use. In the other settings, where there is no control group, the authors utilize a pre-post analysis. In none of the settings do the authors find evidence of an impact on contraceptive use. Finally, the authors extend their estimates to a cost analysis and find that each program’s cost per DALY averted was well beyond the GDP per capita in each setting.

The authors should be congratulated on an impressive amount of work on an important question. Results such as those presented in this paper are important to disseminate because they are comprehensive to an organization’s programming strategy and stand in contrast to the positive effects. With that said, I see several ways to improve this work. Below I outline what I hope will be helpful suggestions to improve the comprehensibility of the work and the credibility of the methods.

1. **Organization:** The authors attempt to comprehensively study the A360 program in this work. However, the interventions, settings, and methods are quite different, and space constraints mean that many important details have been relegated to supplementary information. While I recognize these interventions were pre-registered together, I suggest organizing these analyses into two companion papers. In the first, present the analysis of the Nigeria programs. In the other, group the Ethiopia and Tanzania interventions. This would allow for greater exposition of the interventions and internal continuity in methods (more on this later). I suspect the Nigeria paper in this scenario would be the stronger of the two works.

**Authors :** We believe that it is important to keep the evaluation of the four settings together as all interventions followed the same participatory approach design. The Adolescent 360’s girl centred approach to contraceptive programming is being promoted as an approach <https://a360learninghub.org/about-us/> and not as individual interventions. Our evaluation was designed to capture the impact of this approach across four settings, i.e. four different interventions but designed using the same approach. The interventions and evaluation methodologies are clearly described in the paper, however, we have moved some additional text from the supplementary information to the main text to further clarify the methods (lines 72-78 ; 82-86 of page 4). We agree that the Nigerian evaluation has a stronger evaluation design than the Ethiopia and Tanzania impact evaluation, and we point this out in the discussion section (lines 360-361 of page 29).

a. I certainly sympathize with the challenge of describing interventions that have already been published as protocol papers. However, for the sake of internal completeness, I would avoid simply referring to the BMJ open paper for the reader to view the details of the intervention. I suggest moving Table S1 to the main text.

**Authors :** Table S1 was moved to the main text, as suggested (page 6).

2. **Methods:** The work utilizes two separate empirical strategies, so I will discuss them separately.
   a. **Nigeria (diff-in-diff)**: I find this to be the more convincing of the analyses. However, it is unclear to me how the comparison groups were chosen. I suggest that the authors include a formal test of differences in Table S2 (and potentially move it to the main text). I suspect this analysis will show that the treatment and comparison areas are systematically different. The authors may wish to test the robustness of their estimates to propensity score weighting methods.

**Authors :** We have added: 1) details on how comparison groups were chosen (lines 72-78 of page 4); a formal test of differences between comparison and intervention sites to Table S2, now called Table S1; and a point in the discussion (lines 367-370 of page 30). The demographics characteristics did not change much over time (Table S1) therefore we suspect that adjustment (either via regression or propensity score methods) will have minimal impact.

i. I suspect the authors will also face criticism for their lack of discussion of the parallel trends assumption. They may attempt to utilize the Nigeria DHS to analyze trends between enumerated areas, albeit at a higher level.

**Authors :** We have used HMIS data to explore the trends in the various enumeration areas, the analysis of this data is presented in File S1, section “Methods and results for the analysis of trends in modern contraceptive use prevalence”. We made this information clearer. We also added a discussion paragraph on this (lines 370-377 of page 30). These data reflect a broader age range than was included in the survey population and provided only weak evidence on the validity of the parallel trend assumption.

b. **Ethiopia and Tanzania (Pre-Post Analysis):** With the surveys' pre/post analysis and cross-sectional nature, it is impossible to rule out the possibility that the results are driven by trends unrelated to the A360 program. I suggest that the authors present these results in a separate paper and frame them as descriptive and related to a process evaluation of the A360 program rather than an attempt to estimate the effects of the program.

**Authors :** We agree that the Nigeria impact evaluation is stronger than the Ethiopia and Tanzania impact evaluation, and we have made this clearer in the discussion section. Since the Ethiopian and Tanzanian evaluations were designed to be impact evaluations, and the designs published as such on our Protocol paper ( Reference: <https://bmjopen.bmj.com/content/8/5/e021834.abstract> ) we do not think it is appropriate at this stage to change their description to a process evaluation. The study included a separate process evaluation in all countries ( Reference: <https://www.itad.com/wp-content/uploads/2022/06/A360-pages-for-report-2021-May22-1.pdf> ). We believe that it is important to keep the evaluation of the four settings together, for the arguments mentioned above. We are transparent about what we agree are important limitations of the study designs across all countries throughout the paper.

c. **Common Issues:**
   i. Panel surveys would provide a more convincing analysis of the program. Recognizing that this was not possible, I think providing more description of the sampling protocol is warranted.

**Authors :** Since the target population were adolescent girls aged between 15 to 19 years, we could not interview exactly the same girls in the two surveys, as some girls interviewed at baseline aged out and could no longer be included at endline. The same procedures and areas were visited at baseline and endline, but no attempt was made to trace individuals or households from baseline to endline. We have clarified this in the text (lines 82-86 of page 4).

ii. I don't believe the authors can make statements about the program's effectiveness with program exposure so low in each intervention-arm survey (less than 25 percent in each setting if I’m reading correctly). Indeed, in Table 2, the individual-level analysis provides suggestive evidence that the programs were very effective among women who received them. Instead, the low self-reported participation density may represent an implementation or scale issue or low demand for family planning services.

**Authors :** We have made our interpretation of the data clearer throughout the paper and in lines 452-457 of page 33 in the discussion. The study design set out to estimate the population level impact of the A360 programme in the target geographies under real world conditions, and, with limitations that are described in the paper, we interpret the data in line with this aim. We agree that this is different to understanding whether individuals who participated in aspects of the programme were impacted by this participation, and that a key link between these two questions is the population level coverage which as the reviewer points out was low. Again acknowledging the limitations, we additionally seek to cautiously interpret the results of the analyses that look at outcomes among those who self-report exposure to the programme compared to those who do not.

iii. Relatedly, the individual analysis in Table 2 is subject to selection bias. In Nigeria, the authors may be able to include both intervention and control areas, allowing them to use residence in an intervention area as an instrumental variable for program participation. This may be a weak instrument given the low program uptake, but if not, this would give the authors a less biased estimate of the local average treatment effect.

**Authors :** We acknowledge the interest of such an instrument. However, we also think that given the low program participation, and the lack of understanding for it being low, we believe that the instrument would unfortunately be weak indeed. We acknowledge potential biases in our analytic strategy and agree with the reviewer that the individual analysis may indeed be biased.

Some more minor points:

1. In the companion protocol paper, the authors describe a dose-response analysis that I don’t believe can be found in this work.

**Authors :** Initially, our aim was to create several levels of exposure, ex. “no exposure”, “some exposure” and “high exposure” to the program. Unfortunately, due to the low level of self-reported exposure, we were not able to quantify the effect of greater or lower participation in program activities, as initially planned. This would have allowed us to further explore the effect of A360 exposure on the outcomes of interest.

2. The authors conduct their analysis on complete cases. Are these results robust to other methods of dealing with incomplete information on covariates?

**Authors :** The proportion of missing data on demographic variables was very low – generally 0% and never more than 5% across the four sites. The amount of bias that could result from this level of missing data is not likely to be masking an intervention effect. The total number of individuals considered in each model is presented in the last column of S3 Table for Nasarawa and Ogun; the total number of primary sampling units used in each model is presented in the last column of S3 Table for Oromia and Mwanza.

3. It is unclear to me that DALYs are the right unit to use when conducting the cost analysis. Instead, I may present the cost per percentage point increase in the mCPR.

**Authors :** The cost per DALY averted gives a standard benchmark for comparison to other health interventions in a way we would not have if we were to use cost per percentage point mCPR increase. DALY is a standard measure of health impact and thus better suited to an economic evaluation of the type we carried out. mCPR as a rate is dependent on a denominator (number of eligible users) whose size may vary depending on the size of the study group. The health impact (and associated costs) of any effort to increase mCPR by one percentage point will thus vary depending on the size of that denominator.

**Reviewer #2**

This is an impressive and enormous project and your evaluation accordingly.

My suggestions to make the manuscript easier to read for those not as familiar with your acronyms and methods are detailed below.

**Intro or methods**

1. Please consider giving a more detailed account of how Covid19 affected your study areas and potentially the impact of interventions in the study other than survey methods. Consider this in your discussion.

**Authors :** We have added a paragraph on this in the discussion (lines 422-444 of pages 32 and 33).

2. P.5. Lines 100- It is to your considerable credit that you included a design effect in your sampling to take account of cluster, thus increasing the sizes you needed to reach. Many studies don't. Why not state this.

**Authors :** We described the design effect use, and stated it as a strength in the discussion (lines 110-111 of page 6).

3. Please explain that while 'modern contraception included many effective and not so effective methods, that you included a measure of LARC in your outcomes. It is important but is not clear to the reader if they don't read all your supplements.

**Authors :** LARC was measured due to the large global emphasis put on this type of methods to offer girls with access to the widest available contraceptive options. We added a sentence on this in the methods (lines 153-157 of page 16).

Results

4. You appeared to me to achieve your sample size in three out of four areas. I think you should make a more comprehensible description of your actual samples. I found Figure 1 complex and difficult to understand what you meant by base and endline samples that look as if you are fine, but below you state the actual number interviewed and some are quite small.

**Authors :** Response rates were high in all 4 settings at baseline, ranging between 69% in Tanzania at baseline, to 97% in Northern Nigeria. Similar results were found at endline. Please note that sample size calculations took into account the the fact that not all girls were sexually active, which means that, particularly for unmarried girls, the target sample size/ or the number of girls approached is larger than the number of girls interviewed.

5. Please label the purpose of each of your Supplementary files at the top please.

**Authors :** This was added (pages 42-43).

Discussion

6. P 21 line 337 - explain again what HCD is please.

**Authors :** An explanation of HCD was added to the paragraph (lines 383-387 of pages 30-31).

7. Ogun sample is very wealthy compared to much of the other areas. Would this have had any effect on the outcome?

**Authors :** If the background trend in mCPR depends on wealth then this could cause bias (even after adjusting). However, from Table S2, the intervention and control areas in Ogun are similar in terms of wealth.

Congratulations on a logistically very complex but important study.
