# Supplementary material for: Effect and cost-effectiveness of human-centred design-based approaches to increase adolescent uptake of modern contraceptives in Nigeria, Ethiopia and Tanzania: Population-based, quasi-experimental studies
Source: PLOS Glob Public Health. 2023 Oct 18;3(10):e0002347. doi: 10.1371/journal.pgph.0002347 (PMC10584105; doi:10.1371/journal.pgph.0002347)
Supplement: Attachment — Submitted filename: Response to reviewers.docx [file pgph.0002347.s010.docx]

**Journal Requirements:** Please include a complete copy of PLOS’ questionnaire on inclusivity in global research in your revised manuscript. Our policy for research in this area aims to improve transparency in the reporting of research performed outside of researchers’ own country or community. The policy applies to researchers who have travelled to a different country to conduct research, research with Indigenous populations or their lands, and research on cultural artefacts. The questionnaire can also be requested at the journal’s discretion for any other submissions, even if these conditions are not met. Please find more information on the policy and a link to download a blank copy of the questionnaire here: https://journals.plos.org/globalpublichealth/s/best-practices-in-research-reporting. Please upload a completed version of your questionnaire as Supporting Information when you resubmit your manuscript.

**Authors:** We have verified and completed the information in the questionnaire on inclusivity in global research.

**Journal Requirements:** Please review your reference list to ensure that it is complete and correct. If you have cited papers that have been retracted, please include the rationale for doing so in the manuscript text, or remove these references and replace them with relevant current references. Any changes to the reference list should be mentioned in the rebuttal letter that accompanies your revised manuscript. If you need to cite a retracted article, indicate the article’s retracted status in the References list and also include a citation and full reference for the retraction notice.

**Authors:** We have verified that the reference list is complete and correct.

**Additional Editor Comments:** This is an important evaluation of a flagship adolescent reproductive health program. Both the findings and reflections on methodological limitations encountered will be of great interest to health practitioners and policy makers engaged in or considering similar programs in other settings. In the spirit of open science, the inclusion of a detailed annex including intervention characteristics and documentation of analyses conducted is also appreciated and will serve as an example for other researchers.

It is interesting to note that A360 approaches are being scaled up in Ethiopia and Nigeria, and expanded to Kenya, despite evidence that it is not a cost-effective intervention package and is not designed to address social norms that remain a barrier to adolescent contraceptive use across program locations. I was somewhat surprised to read that the program is being expanded, and feel it merits further attention in the Discussion. Can the authors provide additional insights into the decision to scale-up? Were modifications to the intervention approach recommended based on evaluation results, and are these being adopted in expansion sites?

**Authors:** We approached the implementer (Population Services International, PSI) to request further insights into the rationale for the scale-up. They reported that the decision to scale up was driven by the governments of the implementing countries following their review of the cost-effectiveness studies presented in this manuscript and PSI’s internal evidence and learning. The intervention approach in the current phase of A360 has been adapted to strengthen components addressing social norms (reference here <https://gatesopenresearch.org/articles/6-14>). However, PSI’s spokesperson also noted that the intervention is unlikely to make substantial progress on creating a more supportive environment at a population level given the relatively short length of the project and the resources available. We have added the following text (in bold) to the last paragraph of the discussion:

*“Our results highlight the challenges associated with identifying cost-effective approaches to increase the voluntary use of modern contraceptives among adolescents. A360 is being scaled-up and modified in Ethiopia and Nigeria, and expanded to Kenya [44].* ***According to the A360 implementers (PSI), the decision to scale-up was driven largely by the governments of implementing countries.*** *We recommend increased efforts to address social norms that prevent girls from accessing or using contraceptives as well as additional evaluation of A360 among both married and unmarried adolescents.* ***Accordingly, A360’s second phase aims to have a more consistent and rigorous approach to improving the enabling environment [44]. The modified A360 approaches are going to be evaluated through new cost-effectiveness studies.”***
